# L-arginine and Vitamin D Adjunctive Therapies in Pulmonary Tuberculosis: A Randomised, Double-Blind, Placebo-Controlled Trial

**DOI:** 10.1371/journal.pone.0070032

**Published:** 2013-08-14

**Authors:** Anna P. Ralph, Govert Waramori, Gysje J. Pontororing, Enny Kenangalem, Andri Wiguna, Emiliana Tjitra, Sandjaja, Dina B. Lolong, Tsin W. Yeo, Mark D. Chatfield, Retno K. Soemanto, Ivan Bastian, Richard Lumb, Graeme P. Maguire, John Eisman, Ric N. Price, Peter S. Morris, Paul M. Kelly, Nicholas M. Anstey

**Affiliations:** 1 Global and Tropical Health Division, Menzies School of Health Research, Darwin, Northern Territory, Australia; 2 Department of Medicine, Royal Darwin Hospital, Darwin, Northern Territory, Australia; 3 Public Health and Malaria Control Department, PT Freeport Indonesia, Timika, Papua Province, Indonesia; 4 Menzies School of Health Research–National Institute of Health Research and Development Research Program, Timika, Papua Province, Indonesia; 5 District Health Authority, Timika, Papua Province, Indonesia; 6 International SOS, Timika, Papua Province, Indonesia; 7 National Institute of Health Research and Development, Jakarta, Indonesia; 8 Department of Microbiology, Faculty of Medicine, University of Indonesia, Jakarta, Indonesia; 9 Institute of Medical and Veterinary Pathology, Adelaide, South Australia, Australia; 10 School of Medicine and Dentistry, James Cook University, Cairns, Queensland, Australia; 11 Baker IDI Heart and Diabetes Institute, Alice Springs, Northern Territory, Australia; 12 Garvan Institute of Medical Research, Darlinghurst, New South Wales, Australia; 13 ACT Health, Canberra, Australian Capital Territory, Australia; Menzies School of Health Research, Australia

## Abstract

**Background:**

Vitamin D (vitD) and L-arginine have important antimycobacterial effects in humans. Adjunctive therapy with these agents has the potential to improve outcomes in active tuberculosis (TB).

**Methods:**

In a 4-arm randomised, double-blind, placebo-controlled factorial trial in adults with smear-positive pulmonary tuberculosis (PTB) in Timika, Indonesia, we tested the effect of oral adjunctive vitD 50,000 IU 4-weekly or matching placebo, and L-arginine 6.0 g daily or matching placebo, for 8 weeks, on proportions of participants with negative 4-week sputum culture, and on an 8-week clinical score (weight, FEV_1_, cough, sputum, haemoptysis). All participants with available endpoints were included in analyses according to the study arm to which they were originally assigned. Adults with new smear-positive PTB were eligible. The trial was registered at ClinicalTrials.gov NCT00677339.

**Results:**

200 participants were enrolled, less than the intended sample size: 50 received L-arginine + active vitD, 49 received L-arginine + placebo vit D, 51 received placebo L-arginine + active vitD and 50 received placebo L-arginine + placebo vitD. According to the factorial model, 99 people received arginine, 101 placebo arginine, 101 vitamin D, 99 placebo vitamin D. Results for the primary endpoints were available in 155 (4-week culture) and 167 (clinical score) participants. Sputum culture conversion was achieved by week 4 in 48/76 (63%) participants in the active L-arginine versus 48/79 (61%) in placebo L-arginine arms (risk difference −3%, 95% CI −19 to 13%), and in 44/75 (59%) in the active vitD versus 52/80 (65%) in the placebo vitD arms (risk difference 7%, 95% CI −9 to 22%). The mean clinical outcome score also did not differ between study arms. There were no effects of the interventions on adverse event rates including hypercalcaemia, or other secondary outcomes.

**Conclusion:**

Neither vitD nor L-arginine supplementation, at the doses administered and with the power attained, affected TB outcomes.

**Registry:**

ClinicalTrials.gov. Registry number: NCT00677339

## Introduction

Simple and inexpensive strategies to improve outcomes from tuberculosis (TB) are highly sought-after. Adjunctive immunotherapies have been acknowledged as a priority TB research area [1], and interest in the use of supplemental nutritional agents for TB has been longstanding [2]. Vitamin D, a micronutrient derived from dermal ultraviolet B (UVB) radiation and some dietary sources, and L-arginine, a conditionally-essential amino acid, have antimycobacterial properties directly or via downstream mediators, rendering them suitable as candidate adjunctive immunotherapies in TB [3].

Evidence of a pivotal role of activated vitamin D (1,25-dihydroxycholcalciferol, 1,25D) in TB arises from basic science [4], [5], clinical research [6] and observational studies [7]. Vitamin D (vitD) sufficiency has been hypothesised to decrease TB infection risk after exposure [8], limit progression from latent to active TB [9], or, as an adjunct to antimicrobial therapy, decrease the duration or improve the effectiveness of treatment [3], [10], [11]. 1,25D promotes mycobacterial killing in macrophages through production of antimicrobial peptide LL-37, after activation of macrophages via either toll-like receptor [4] or IFNγ pathways [5], and by promoting phagolysosyme fusion [12] and autophagy [5], [13], [14]. Apparent insufficiency or deficiency of vitD, as estimated from plasma 25-hydroxyvitamin D concentration (25(OH)D), is frequently reported in people with active TB [7], [14]. In a small prospective study of TB contacts, those with lowest vitD levels had the highest risk of progression to active TB [15]. Administration of vitamin D_2_ 2.5 mg to TB-exposed people enhanced their innate antimycobacterial responses (BCG-*lux* assay), compared with control subjects [16].

Two randomised controlled trials (RCT) of adjunctive vitD in 365 [11] and 126 [10] patients with PTB respectively found no benefits overall, but in one the vitD dose was low [11], and in the other using a higher dose, significantly improved outcomes were seen in the subgroup with the *tt* genotype of the *Taq*I vitD receptor polymorphism [10], and time to sputum microscopy conversion was improved in the vitD arm in per-protocol analysis [17]. A further RCT has reported high-dose vitD supplementation to be associated with improved non-microbiological outcomes among 132 TB patients compared with controls [18].

Although exposure to ultraviolet B (UVB) irradiation is the main determinant of vitD status, deficiency is well-documented at low (tropical) latitudes [19]–[25], since clothing, lifestyles and meteorological determinants (cloud cover/rainfall) may limit UVB exposure. Thus vitD remains relevant in high-TB burden settings, and studies of TB patients in the tropics consistently show lower serum 25(OH)D in TB patients than local healthy controls [14].

L-arginine is the sole biological precursor of nitric oxide (NO), a molecule with key immunological functions. L-arginine is converted to NO in macrophages by nitric oxide synthase 2 (NOS2). NO is capable of killing TB bacilli *in vitro* with a molar potency exceeding that of antibiotics [26]. Although the relative importance of NO and vitD pathways in murine and human macrophages is debated, there is firm evidence that human macrophages/monocytes produce NOS2 in response to *M.tb* infection, and that NO concentrations correlate with *M.tb* inhibition [3]. Pulmonary NO bioavailability is impaired in pulmonary TB in proportion to clinical severity and is associated with delayed mycobacterial clearance with treatment [27], [28]. Hypoargininemia, and consequently an impaired capacity to generate NO, can develop when arginine catabolism exceeds endogenous synthesis. Hypoargininemia has been demonstrated in TB [29]. Additional to NO-related immunological effects, L-arginine can influence antimycobacterial cell-mediated responses directly, being required for expression of the T cell receptor's CD3ζ component [29].

Two trials have addressed L-arginine in TB, testing L-arginine hydrochloride (1 g) [30] or arginine-rich food (peanuts) [31]. In the first (n = 120), faster sputum-microscopy clearance and cough resolution were reported in the supplemented arm, but only among HIV-negative participants [30]. The second study (n = 180) reported no significant benefits overall, but post-hoc analyses found higher cure rates in HIV-positive participants receiving the arginine-rich supplement [31].

Given the *in vitro* and *in vivo* data indicating antimycobacterial roles of vitD and L-arginine, encouraging but inconsistent findings from clinical trials to date, and the appeal of inexpensive nutritional agents as adjunctive therapies, we aimed to investigate whether vitD and L-arginine, given with standard TB treatment, would result in more rapid improvement in microbiological and clinical outcomes among adults with PTB. We also hypothesised that L-arginine administration would be associated with increased pulmonary NO production.

## Methods

The protocol for this trial and supporting CONSORT checklist are available as supporting information; see Checklist S1 and Protocol S1.

### Study setting

Timika, population ∼200,000, comprising approximately half Indigenous Papuans and half Non-Papuan Indonesians, is at latitude 3°S in southern Papua Province, Indonesia. Papua is relatively disadvantaged within Indonesia, including higher TB (318/100,000 in 2007 [32]) and HIV rates [33]. UV radiation is approximately constant year-round, UV radiation is approximately constant year-round, with annual rainfall, and hence cloud cover, being extremely high (approximately 3800 mL rain per annum) [34].

### Participants

Consecutive patients referred to Timika TB clinic with newly diagnosed with PTB (≥2 direct sputum specimens positive for acid fast bacilli [AFB]) were assessed for eligibility: sputum smear positive, >15 years, not pregnant, without hypercalcaemia (ionized calcium ≤1.32 mmol/L), not previously treated for TB, agreeing to stay in Timika for 6 months and providing written informed consent. Written informed consent was obtained after discussion in Indonesian or a relevant Papuan language aided by pictorial and written information.

### Ethics statement

The study was approved by the Approval was granted by the Human Research Ethics Committees of Menzies School of Health Research, Darwin, Australia (07/40) and the National Institute for Health Research and Development, Jakarta, Indonesia (LB.02.02/KE/438/2008).

### Trial design and interventions

This study was a double-blind factorial 2×2 design (4 arms containing 2 interventions or their matching placebos), chosen to maximise efficiency. Participants received directly-observed anti-TB therapy (weight-dosed rifampicin, isoniazid, pyrazinamide, ethambutol daily ×2 months, then rifampicin, isoniazid thrice weekly ×4 months). At TB treatment commencement, participants were randomised to one of: (A) supplementary active L-arginine (L-arginine hydrochloride, ‘Argimax®’) 6 g (6 tablets) daily for eight weeks, plus active cholecalciferol (vitamin D_3_, ‘Calciferol Strong®’) 50,000 IU (1250 mcg, 1 tablet) at baseline and on day 28; (B) active L-arginine plus placebo cholecalciferol (dosing regimen as above); (C) placebo L-arginine plus active cholecalciferol; (D) placebo L-arginine plus placebo cholecalciferol (see Methods). Argimax® and matching placebo were purchased from Hankintatukku Oy, Helsinki, Finland, and Calciferol Strong® and matching placebo from API Consumer Brands, New Zealand. We tested the contents of Argimax® tablets using high performance liquid chromatography (HPLC) and confirmed that the expected concentration of L-arginine was recoverable, with no significant change in L-arginine content after storage in tropical conditions for 16 weeks. Participants were followed for 24 weeks (weekly for 8 weeks then monthly).

### Randomisation and blinding

A block random allocation sequence stratified by ethnicity (Papuan/Non-Papuan) was generated (Stata 9.1) and remained concealed from all investigators throughout the study. Independent assistants prepared study medication packs, labelling them with a code corresponding to the randomisation sequence. Participants were assigned the next sequential code, and dispensed an opaque envelope containing the study medications. Active and placebo medications appeared identical.

### Outcomes and adverse events

Primary outcome measures were the proportion of participants with negative sputum culture on liquid medium at week 4, and a composite clinical severity score at week 8. The clinical score (Table 1) allocated points at week 8 for %change in weight and FEV_1_, cough (worse or same, improved, ceased), and presence/absence of sputum and haemoptysis. Secondary outcomes were safety (death, hospitalisation, hypercalcaemia); sputum smear conversion time (≥2 consecutive negative smears without a subsequent positive); change in 6-minute walk test, modified St George's Respiratory Questionnaire, chest radiograph severity score, %predicted forced expiratory volume in one second (FEV_1_); and primary end points stratified by HIV status and ethnicity.

**Table 1:** Composite clinical outcome score.*

Clinical parameter		Point assigned
**% Weight change**	Decrease	0
	<5% weight gain	2
	5.0–9.9% weight gain	4
	≥10% weight gain	6
**% FEV1 change**	≥10% fall in FEV1	0
	<10% fall or <10% improvement in FEV1	1
	≥10% FEV1 improvement	2
**Cough**	Worse or same	0
	Improved	1
	Ceased	2
**Sputum**	Present	0
	Absent	1
**Haemoptysis**	Present	0
	Absent	1
**Maximum score**		12

* The score was devised *pre hoc* by consensus opinion among the investigators. We assigned greatest significance to weight gain, due to its leading clinical importance in response to TB treatment [57]–[61], and its reliable objectivity. The selected cut-off of weight gain <5% was guided by previously-published results [57]. The lung function cut-offs were guided by knowledge of test repeatability (accuracy) and plausible improvements in FEV_1_ during the given time frame [38].

Serious adverse events (SAE) comprised death, hospitalisation or life-threatening conditions. Adverse events (AE) comprised new symptoms or hypercalcaemia. Ionised calcium concentration (*i*Ca^2+^) was measured using a point-of-care iSTAT® device at weeks 0, 2, 4, 8.

### Clinical and laboratory procedures

Postero-anterior chest radiographs performed at weeks 0, 8, 24 were reported blinded to randomisation arm according to a previously-validated x-ray score [35]. Pulmonary function was measured outdoors (for infection control purposes) using a handheld spirometer (MicroLoop®, MicroMedical, UK) with filtered one-way mouthpieces (Sure-Gard®).

Fractional exhaled NO (FE_NO_), which evaluates pulmonary NO production, was measured using a portable NiOX MINO® (Aerocrine, Sweden). This device is well-validated [36] and employs disposable, filtered mouthpieces without infection control risks. FE_NO_ measurements complied with ATS 2005 Guidelines [37] (inhalation of NO-free air through the mouthpiece, then exhalation for 10 seconds at 50±5 mL/s, with avoidance of prior eating/exercising). Regular quality controls measures were performed, described elsewhere [28].

The 6-minute walk test (6MWT) was assessed on an open air path beside the clinic. The St George's Respiratory Questionnaire (SGRQ), Indonesian translation (Professor P.W. Jones, St George's Hospital Medical School, London) and slightly modified for local conditions was used as previously in TB patients in Timika [38]. The minimum clinically important difference in SGRQ is considered to be approximately 4 units [39].

At the field laboratory, Ziehl-Neelsen (ZN) staining for AFB was performed on direct sputum specimens at weeks 0–8, then weeks 20 and 24. Duplicate samples were obtained at weeks 0, 4 and 8, batched at 4°C and dispatched unrefrigerated to the University of Indonesia's Faculty of Microbiology, Jakarta, for culture and drug susceptibility testing (DST). Transit time was approximately 3 days. Unrefrigerated transportation of fresh sputum samples has been shown to provide good *M.tb* recovery rates [40]. Sputum was decontaminated using 2% sodium hydroxide and 0.5% N-acetyl-cysteine, neutralised to pH7, concentrated by centrifugation at 3000×g for 15 minutes, and inoculated into a BACTEC® Mycobacterium Growth Indicator Tube (MGIT) 960 tube and two Lowenstein-Jensen tubes. DST was performed using the MGIT 960TB system for isoniazid, rifampicin, ethambutol, streptomycin and, in the instance of MDR-TB, ofloxacin, amikacin and kanamycin. Confirmation of MDR-TB or rifampicin-monoresistance was achieved with Hain GenoType® MTBDR*plus* assay.

Quantitative serum L-arginine concentration was to be tested using high performance liquid chromatography, 25(OH)D levels using isotope-dilution liquid chromatography-tandem mass spectrometry (LC-MS/MS), and 1,25(OH)2D3 and parathyroid hormone using radioimmunoassays. However, after study commencement, changes by the Indonesian Ministry of Health in the implementation of pre-existing materials transfer agreements pertaining to sample export from Indonesia, meant that it has not been possible to undertake these planned blood tests.

### Statistical methods

The sample size was calculated using the graphical method for 2×2 factorial designs [41]. At a 2-tailed significance level of 5%, a sample size of 444 (111 participants in each arm) would provide 82% power to demonstrate that each treatment results in a 20% reduction in the proportion culture positive at one month (from 60% to 40%), assuming losses to follow up of 10%. We estimated at least 60% of patients would be culture positive at 4 weeks, based on locally-identified bacillary burden [38], [40] and extent of cavitary disease [35], and published mean times to culture negativity in mostly drug-sensitive TB [42], [43]. According to recommendations for factorial trials, the sample size was inflated by the precautionary inclusion of an interaction coefficient of 0.5, in the event that any benefits of L-arginine and vitD might have been sub-additive. The likelihood of L-arginine/vitD interaction could not be confidently estimated since no prior clinical studies exist, and *in vitro* findings conflict: vitamin D was found to inhibit NOS2 expression in one study [44], yet upregulate *NOS2A* in another [45]; the latter study also found the suppressive effect of vitamin D on *M.tb* replication to be partially impaired if nitric oxide formation was inhibited. Hence we made a conservative assumption of a sub-additive effect, although acknowledge that synergism might be possible. The final power based on the obtained sample size was approximately 64% (see Discussion).

Analyses were conducted using Stata 12.1 (StataCorp. 2011, College Station, TX) according to a pre-specified plan. Outcomes were analysed according to the arm to which the participant was originally assigned. The first primary outcome (sputum culture at week 4) was tested using χ^2^-test; the second (composite clinical score) using Student's 2-sample T-tests. Fisher's Exact test was used for analyses stratified by HIV status. Multivariable logistic regression models were used to adjust for co-interventions, ethnicity, age, sex, HIV status, smoking status, presence of MDR-TB and adherence. FE_NO_ data were log-transformed or compared using Wilcoxon Rank-sum test. Logistic regression models were used to test for interactions between interventions. Multivariable logistic regression models were used for post-hoc analyses adjusting for baseline differences between study arms. In modified intention-to-treat analyses, participants with protocol violations or poor adherence were excluded. Kaplan-Meier survival analysis was used to examine sputum smear conversion time; patient subgroup analyses were performed by Cox regression (proportional-hazards) models and hazard ratios and 95% confidence intervals. The Data Safety Monitoring Committee undertook an interim safety analysis after 25% of participants had been enrolled and reviewed each SAE, to advise on the safety of study continuation.

## Results

**Figure 1:**
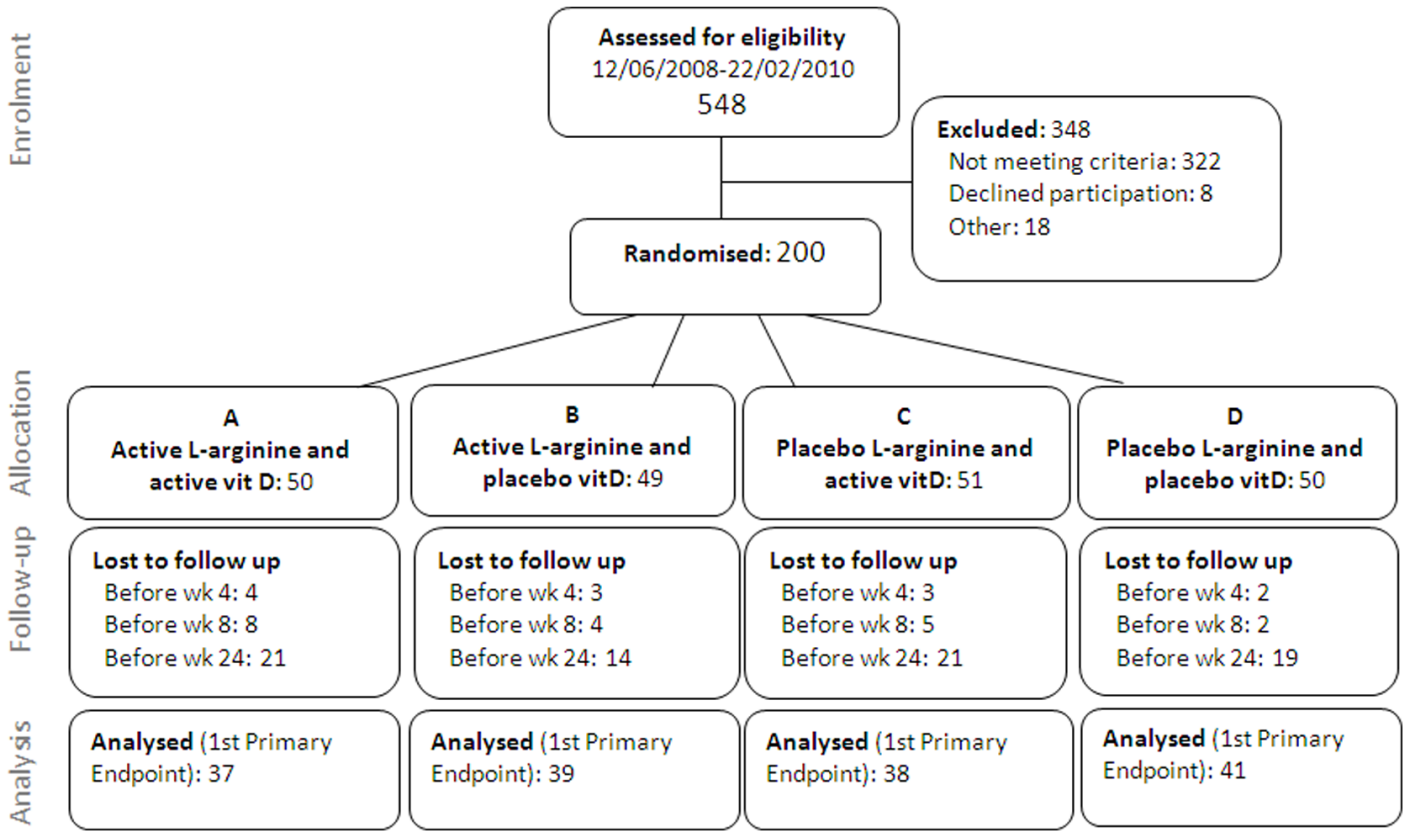
Trial summary.

Two hundred participants were enrolled June 2008-February 2010. New enrolments ceased on 22^nd^ February 2010 when 45% (200/444) of the planned sample size had been recruited because local circumstances at the field site prevented continuation of the trial. Study participants' baseline characteristics are shown in Table 2. By chance, there were differences in sex, HIV status and X-ray severity at baseline. Sputum culture results were available for 178 participants at enrolment and 155 at week 4. Reasons for missing results included: specimen lost in transit, contamination, power-outage in the laboratory, or participant loss to follow-up prior to week 4 (Figure 1).

**Table 2:** Baseline characteristics.

	All patients	L-arginine (Arms AB)	No L-arginine (Arms CD)	Vitamin D (Arms AC)	No Vitamin D (Arms BD)
**Number of study participants**	200	99	101	101	99
**Age in years: median (range)**	28 (15–73)	27 (15–65)	28 (15–73)	29 (15–65)	26(15–73)
**Papuan: no. (%)**	89 (44.5)	44	45	45	44
**Female: no. (%)** *	69 (34.5)	42	27	37	32
**HIV positive: no./no.tested (%)** †	19/145 (13.1%)	12 (17.4)	7 (9.2)	15 (21.4)	4 (5.3)
Papuan	15/71 (21.1)				
Non-Papuan	4/74 (5.4)				
**Weight (kg): mean(range)**	48.5 (26.3–74.0)	48.5 (26.3–74.0)	48.5 (36.0–63.5)	48.6 (33.0–74.0)	48.3 (26.3–63.5)
**BMI (kg/m^2^): mean (range)**	19.2 (12.0–32.5)	19.2 (12.0–32.5)	19.1 (15.2–24.5)	19.1 (13.3–32.5)	19.3 (12.0–26.3)
**Smoking status: no. (%)**					
Current	56 (28)	25	31	27	29
Ex-smoker	53 (27)	31	22	27	26
Never smoked	91 (46)	43	48	47	44
**Cough: no. (%)**	200 (100)				
Mild-Moderate	106 (53.0)	49	57	55	51
Severe	86 (43.0)	44	42	41	45
Very severe	8 (4.0	6	2	5	3
**Sputum: no. (%)**	199 (99.5)	98	101	100	99
**Haemoptysis: no. (%)**	63 (31.7)	31	32	30	33
**Diagnostic delay in months: median (range)**	2 (0.25–24)	2 (0.25–12)	2 (0.25–24)	2 (0.25–14)	2 (0.25–24)
**FVC (L): mean (range)**					
Female	1.62 (0.63–2.96)	1.54 (0.78–2.96)	1.74 (0.63–2.59)	1.63 (0.63–2.96)	1.61 (0.91–2.48)
Male	2.36 (0.89–4.02)	2.37 (0.84–4.02)	2.35 (1.1–4.45)	2.32 (0.84–4.02)	2.39 (1.01–4.45)
**% Predicted FEV1(L): mean %, 95% CI**	63.3 (60.6–66.0)	61.4 (57.4–65.3)	65.1 (61.4–68.9)	63.1 (59.1–67.0)	63.5 (59.7–67.2)
**Lung function impairment class: no.(%)**					
Normal lung function	47 (23.5)	19	28	26	21
Mild lung function impairment	26 (13.0)	15	11	9	17
Moderate	67 (33.5)	32	35	33	34
Severe	45 (22.5)	24	21	26	19
Very severe	15 (7.5)	9	6	7	8
**St George's Respiratory Questionnaire total score: median (range)**	40.7 (5.2–91.9)	41.7 (5.2–88.5)	36.5 (5.9–91.9)	40.0 (5.9–91.9)	40.7 (5.2–88.5)
**6-minute walk test: median (range)**					
Female	375 (20–490)	368 (20–490)	400 (80–468)	385 (80–459)	364 (20–490)
Male‡	425 (40–612)	400 (40–560)	440 (240–612)	425 (55–600)	425 (40–612)
**Chest X-ray: Cavity size: no.(%)**					
0	69 (44.0)	30 (38.5)	39 (49.4)	38 (46.9)	31 (40.8)
≤4 cm	53 (33.7)	29 (37.2)	24 (30.4)	23 (28.4)	30 (39.5)
>4 cm	35 (22.3)	19 (24.3)	16 (20.2)	20 (24.7)	15 (19.7)
**Chest X-ray: % lung affected: median (IQR)** *	40 (23–62)	48 (26–80)	35 (19–54)	42 (25–73)	36.5 (20.5–57.5)
**Chest X-ray score: median (IQR)** *	68 (34–94)	73 (43–109)	59 (22–86)	70 (36–94)	66.5 (29.5–91)
**Ionised calcium (mmol/L): mean**	1.21 (1.20–1.22)	1.20 (1.19–1.21)	1.22 (1.21–1.23)	1.21 (1.20–1.22)	1.21 (1.20–1.22)
**Sputum acid fast bacilli density: no.(%)**					
0	21 (10.5)	10	11	12	9
Scanty	34 (17.0)	17	17	18	16
1+	57 (28.5)	28	29	33	24
2+	50 (25.0)	27	23	20	30
3+	38 (19.0)	17	21	18	20
**Sputum culture at diagnosis**					
No growth	19 (9.5)	11	8	8	11
*M. tuberculosis* identified	164 (82.0)	80	84	86	78
Contaminated	4 (2.0)	3	1	2	2
No culture result	13 (6.5)	5	8	5	8
***M. tuberculosis*** ** susceptibility**					
Fully susceptible	126/149 (84.6	59/73 (80.8)	67/76 (88.2)	67/80 (83.8)	59/69 (85.5)
INH & RIF resistant (MDR-TB)	2 (1.3)	1	1	1	1

* p<0.05 for difference between arginine and no arginine arms;

† p<0.05 for difference between vitamin D and no vitamin D arms;

‡ p<0.05 for difference between arginine and no arginine arms (males only).

### Microbiological and clinical outcomes

At week 4, 62% (96/155) participants were culture negative overall, higher than predicted in our sample size calculation estimates. This proportion did not differ significantly between active and placebo medication arms (Table 3). In those who received L-arginine plus vitamin D, week 4 culture conversion occurred in 57% (21/37), compared to 61% (25/41) of participants who received neither (risk difference 4%, 95% CI −18 to 27). The effect of the interventions on primary outcomes did not significantly differ by HIV status or ethnicity. The clinical score at 8 weeks was available in 84% (167/200) of participants; this also did not significantly differ between arms (Table 3). We found no evidence of interaction between the interventions (week 4 culture conversion: p = 0.44; clinical score: p = 0.73), although the study was not powered to detect this. Analyses adjusting for co-variables or for baseline differences, and modified intention-to-treat analyses, excluding participants in whom protocol violations or medication adherence problems occurred, did not appreciably alter primary outcome results (data not shown).

**Table 3:** Primary Outcomes.

	All	L-arginine (Arms AB)	No L-arginine (Arms CD)	p	Vitamin D (Arms AC)	No Vitamin D (Arms BD)	p
**N**	200	99	101		101	99	
**Culture negative at week 4: no.(%)**	96/155 (61.9)	48/76 (63.2)	48/79 (60.8)*	0.76	44/75 (58.7)	52/80 (65.0)†	0.42
Stratified by HIV status							
HIV−	68/112 (60.7)	31/50 (62.0)	37/62 (59.7)	0.80	27/47 (57.5)	41/65 (63.1)	0.55
HIV+	11/19 (57.9)	6/12 (50.0)	5/7 (71.4)	0.63	9/15 (60.0)	2/4 (50)	0.57
Stratified by ethnicity							
Papuan	45/47 (60)	22/37 (59.5)	23/38 (60.5)	0.93	20/36 (55.6)	25/39 (64.1)	0.45
Non-Papuan	51/80 (63.8)	26/39 (66.7)	25/41 (61.0)	0.60	24/39 (61.5)	27/41 (65.9)	0.69
**Clinical score at week 8:**							
Number	168	80	88		81	87	
Mean (SD)	6.9 (1.9)	6.9 (1.9)	6.8 (2.0)	0.81	6.9 (2.0)	6.8 (1.9)	0.68
Stratified by HIV status							
HIV−	6.9 (6.6–7.3)	7.0	6.8	0.65	6.9	6.9	0.86
HIV+	6.7 (5.8–7.6)	6.6	6.9	0.79	6.7	6.5	0.81
Stratified by ethnicity							
Papuan	7.3 (6.9–7.8	7.5	7.2	0.55	7.7	7.1	0.09
Non-Papuan	6.5 (6.1–6.9)	6.5	6.5	0.83	6.3	6.7	0.38
**Composite clinical score components at week 8:**							
**Weight change: no.(%)**							
<5% weight gain	98 (54)	49 (57)	49 (52)	0.74	47 (53)	51 (55)	0.80
5.0–9.9% weight gain	48 (27)	22 (26)	26 (27)		23 (26)	25 (27)	
≥10% weight gain	35 (19)	15 (17)	20 (21)		19 (21)	16 (17)	
**FEV1 change: no.(%)**							
≥10% fall in FEV1	17 (10)	4 (5)	13 (14)	0.10	11 (13)	6 (7)	0.38
<10% fall or <10% improvement	94 (55)	44 (55)	50 (55)		43 (52)	51 (58)	
≥10% FEV1 improvement	60 (35)	32 (40)	28 (31)		29 (35)	31 (35)	
**Cough: no.(%)**							
Worse/same	15 (9)	6 (7)	9 (10)	0.21	6 (7)	9 (10)	0.42
Improved	146 (83)	69 (80)	77 (85)		75 (86)	71 (79)	
Ceased	16 (9)	11 (13)	5 (6)		6 (7)	10 (11)	
**Sputum present: no.(%)**	131 (72)	63 (73)	68 (72)	0.89	62 (70)	69 (75)	0.51
**Haemoptysis present: no.(%)**	3 (2)	2 (2)	1 (1)	0.61	1 (1)	2 (2)	1.00

	Arginine + vitD (Arm A)	Arginine + placebo vitD (Arm B)	Placebo arginine + vitD (Arm C)	Placebo arginine + placebo vitD (Arm D)
**N**	37	39	38	41
**Culture negative at week 4 according to factorial model: no.(%)**	21 (57)	27 (69)	23 (61)	25 (61)

* Risk difference arginine versus arginine-placebo −3%, 95% CI −19 to 13.

† Risk difference vitamin D versus vitaminD-placebo: 7%, 95% CI −9 to 22%.

**Figure 2:**
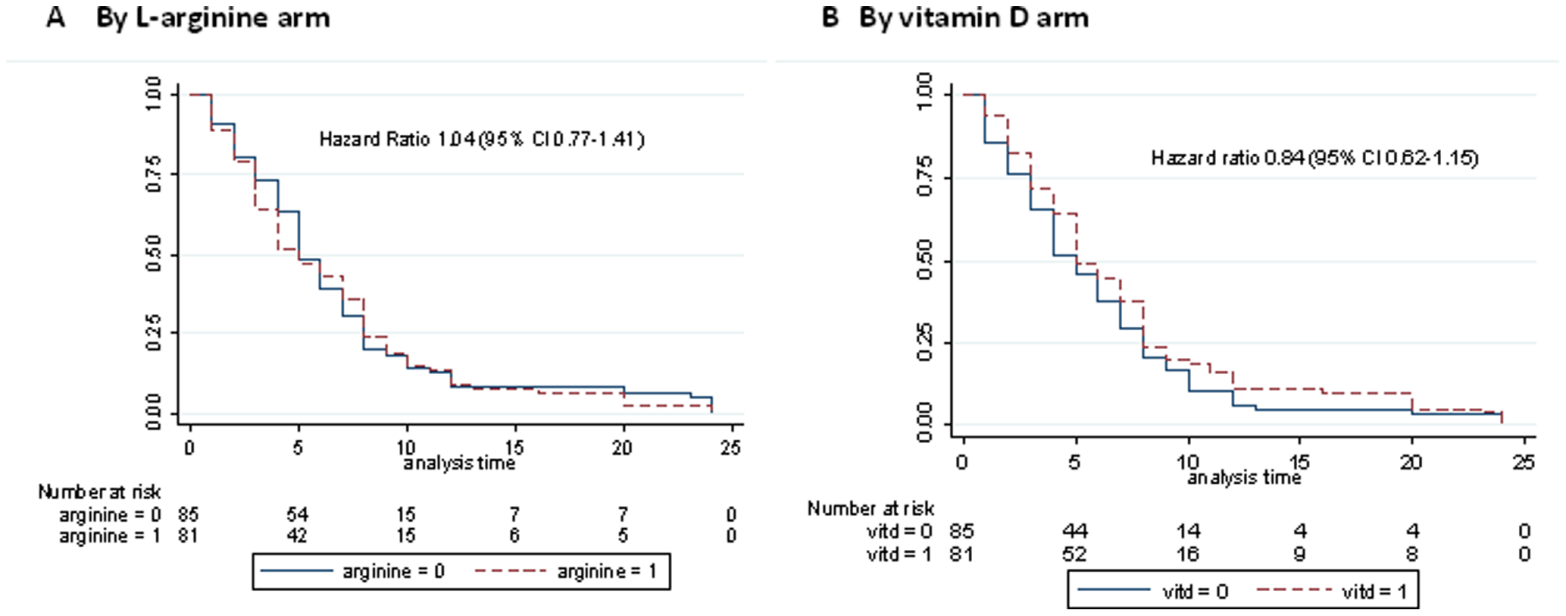
Time to sputum microscopy conversion. A. By L-arginine arm. B. By vitamin D arm.

**Figure 3:**
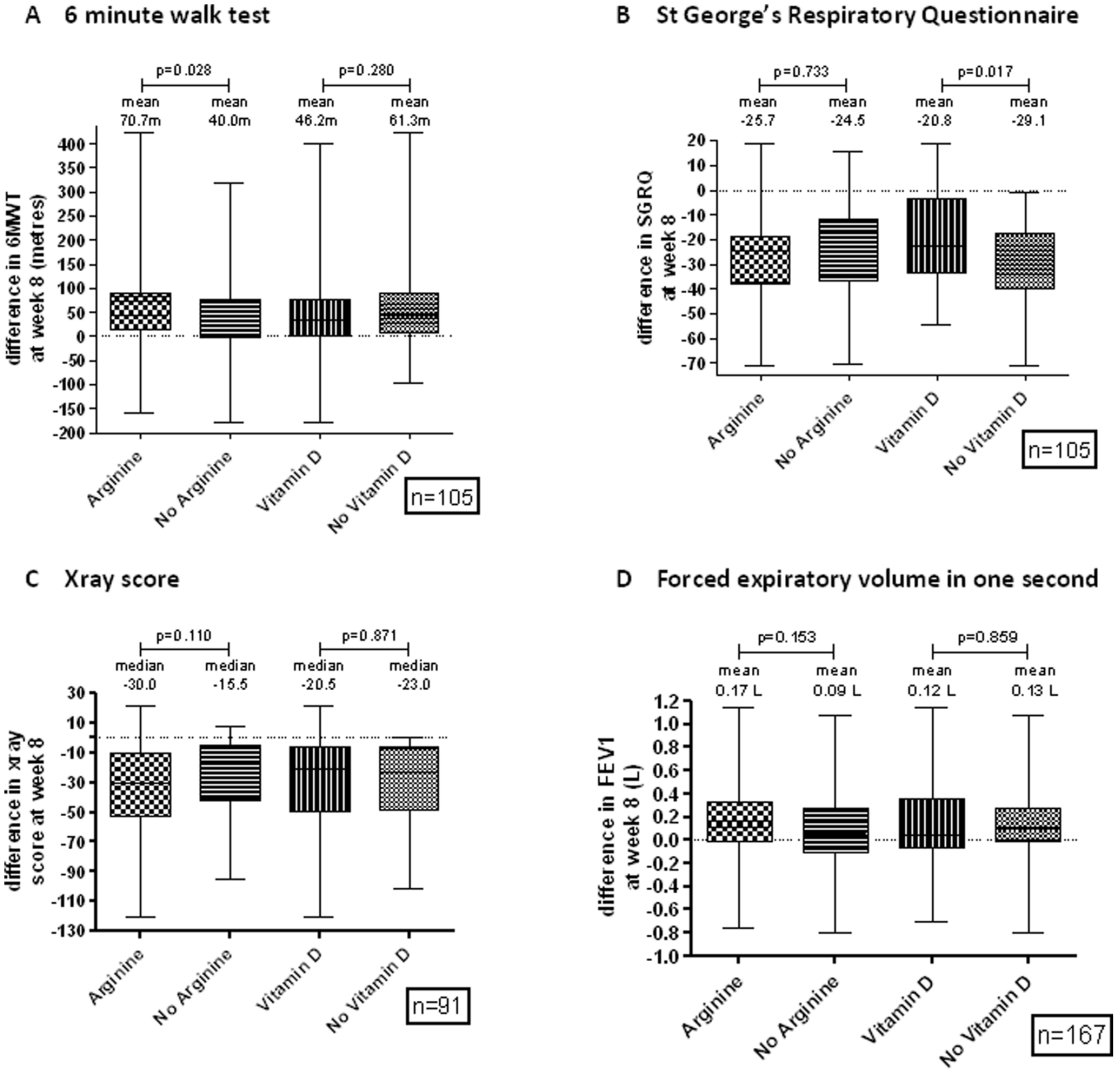
Differences in secondary outcome measures at 8 weeks. A. 6 minute walk test. B. St George's Respiratory Questionnaire. C. X-ray score. D. Forced expiratory volume in one second.

Regarding secondary outcomes, proportions of participants culture-negative at week 8 (Table 4), and time to sputum microscopy clearance (Figure 2), did not differ among study arms. A greater increase in 6MWT occurred in participants in L-arginine vs. L-arginine-placebo groups (Figure 3A), but this may be explained by the baseline difference between these groups in 6MWT (Table 2). A greater fall in SGRQ by 8.3 units occurred in participants who did not receive vitD compared with those who did (Figure 3B). In a post-hoc analysis, week 8 culture-conversion was lower in the vitD than the vitD-placebo arm in HIV-negative participants (p = 0.05), Table 4.

**Table 4:** Secondary Outcomes.

	All	Arginine	No arginine	p	Vitamin D	No vitamin D	p
**N**	200	99	101		101	99	
**Culture negative at week 8: no. (%)**	97/123 (78.9)	44/56 (79)	53/67 (79)	0.94	44/59 (75)	53/64 (83)	0.26
**HIV−**	69/91 (76)	27/36 (75)	42/55 (76)	0.88	24/37 (65)	45/54 (83)	0.05
**HIV+**	13/14 (93)	8/9 (89)	5/5 (100)	1.00	11/12 (92)	2/2 (100)	1.00
**Time to smear negativity: Median weeks (IQR)**	5 (2–8)	4 (2–8)	5 (2–8)	0.85	5 (2–8)	4 (2–8)	0.45
**Serious adverse events:** No. (%)	7 (3.5)	4	3	0.72	4	3	1.00
**Death** *	2 (1.0)	1	1		2	0	
**Hospitalisation** †	5 (2.5)	3	2		2	3	
**Adverse events during weeks 1–8**							
**Hypercalcaemia: No. (%)**	29 (14.5)	12	17	0.34	15	14	0.88
Mild (1.33–1.39 mmol/L)	26 (13.0)	12	14		13	13	
Mod (1.40–1.49 mmol/L)	3 (1.5)	0	3		1	2	
Severe (≥1.50 mmol/L)	0 (0.0)	0	0		0	0	
**Nausea: no. (%)**	66 (33.0)	33	34	0.84	36	30	0.42
**Vomiting: no. (%)**	35 (18)	21	14	0.20	18	17	1.00
**Central nervous system symptom** (headache,dizziness,delirium):no.(%)	107 (55)	51	56	0.52	49	58	0.37
**Itch: no. (%)**	108 (55)	43	44	0.94	41	46	0.30
**Rash: no. (%)**	48 (25)	21	27	0.34	27	21	0.43
**Arthralgia: no. (%)**	118 (61)	59	59	0.93	60	58	0.88

* Deaths: respiratory failure from progressive PTB in an HIV+ 21-year-old male (1 case); stroke complicated by aspiration pneumonia in an HIV+ 60-year-old male (1 case).

† Hospitalisations: pleural effusion complicating MDR-TB (1 case), glucose/electrolyte management issues in diabetics (2 cases), pneumothorax in a malnourished (BMI 12.0 kg/m2) HIV+ female (1 case), and vomiting with dehydration (1 case).

### Adverse events

AE rates but rates did not differ between study arms (Table 5). Hypercalcaemia occurred in 29 people (15%) during weeks 1–8. Most instances were mild; all were asymptomatic. Hypercalcaemia rates were similar in vitD (15%) and vitD-placebo arms (14%); mean *i*Ca^2+^ concentration did not differ between study arms. A small increase in mean *i*Ca^2+^ was observed among all study participants between enrolment (1.21 mmol/L) and week 2 (1.24), p<0.001. SAE occurred in 7 participants (5 hospitalisations, 2 deaths), of whom three had received L-arginine, three vitD, and one both (Table 5). The Data Safety Monitoring Committee deemed these events to be ‘unlikely to be related’ to study medications in 6 instances and ‘unrelated’ in 1 instance.

**Table 5:** Protocol violations and adherence.

	All patients	L-arginine	No L-arginine	Vitamin D	No Vitamin D
**Number of participants**	200	99	101	101	99
**Protocol violations: n (%)**	13 (6.5%)				
Switched study arm with other participant	2	1	1	1	1
Study medications labelled incorrectly	1	1	0	0	1
Met exclusion criterion (had prior TB)	1	1	0	0	1
Smear and culture negative on enrolment sputum specimen*	9	5	4	4	5
**Adherence**					
Full adherence: n (%)	155 (77.5)	76	79	79	76
Missed 1–3 study medication doses: n (%)	17 (8.5)	8	9	7	10
Missed >3 study medication doses: n (%)	28 (14.0)	15	13	15	13
**Second vitamin D/vitamin D placebo dose** †					
Given at week 4	181 (90.5)	87	94	90	91
Given before week 4	2 (1.0)	2	0	2	0
Given after week 4 (wk4–7)	8 (4.0)	3	5	5	3
Never given	9 (4.5)	7	2	4	5
**Losses to follow up: cumulative no.(%)**					
Prior to week 4	12 (6.0)	7 (7.1)	5 (5.0)	7 (6.9)	5 (5.1)
Prior to week 8	19 (9.5)	12 (12.1)	7 (6.9)	13 (12.9)	6 (6.1)
Prior to week 24	75 (37.5)	35 (35.4)	40 (39.6)	42 (41.6)	33 (33.3)

* All participants had been reported AFB+ by the field laboratory on ≥2 pre-enrolment sputum specimens.

† The usual reason for vitD or vitD placebo to be withheld/deferred was hypercalcaemia.

### Effect of arginine supplementation on pulmonary production of nitric oxide

**Figure 4:**
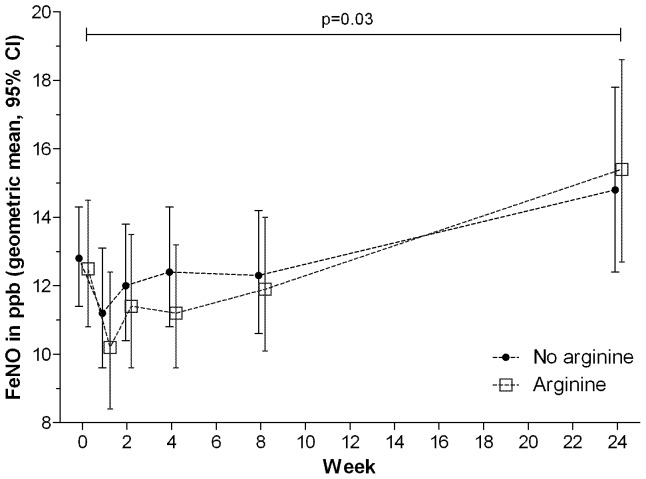
Fractional exhaled nitric oxide over time in L-arginine and L-arginine-placebo study arms.

**Figure 5:**
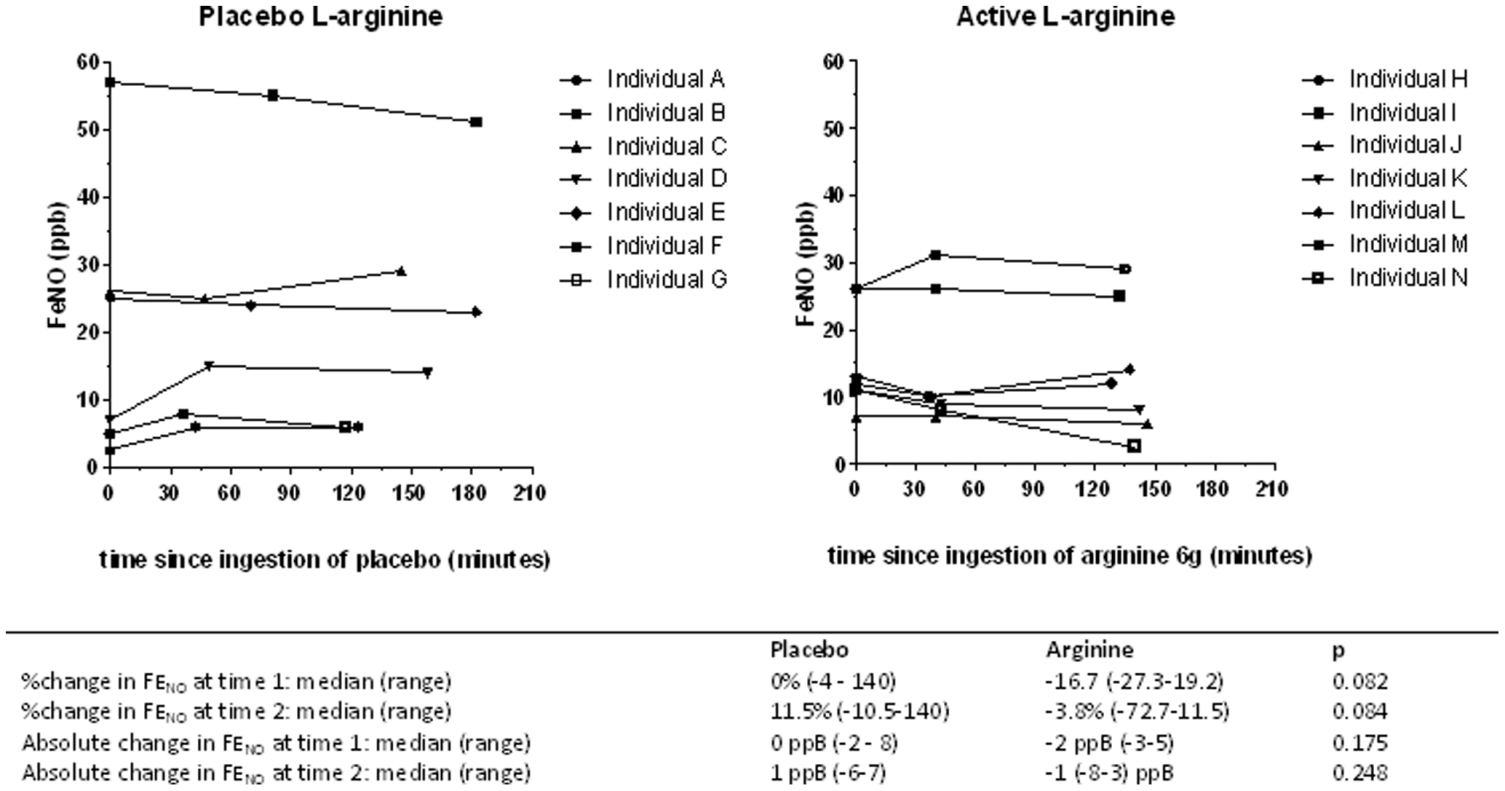
Fractional exhaled nitric oxide before and after ingestion of L-arginine hydrochloride 6.0 g or matching placebo. A. Placebo L-arginine. B. L-arginine.

Participants who received active L-arginine achieved neither higher median FE_NO_, nor greater incremental FE_NO_ change, than those receiving L-arginine-placebo, although in all participants, low initial FE_NO_ concentrations normalised by treatment completion (Figure 4) and [28]. In a subset of participants in whom serial FE_NO_ pharmacodynamic measures were performed blinded before and up to 3 hours after ingestion of 6.0 g L-arginine or L-arginine placebo (the estimated half-life of oral L-arginine being 1.5–2.0 hours) [46], we detected no overall rise in FE_NO_ after active L-arginine administration (median ΔFE_NO_ −2 and −1ppB at first and second time points respectively), nor difference compared with those administered placebo (Figure 5).

## Discussion

We report the first study to evaluate L-arginine with vitD as adjunctive TB therapies. At the doses evaluated, we could not demonstrate that these agents alone or in combination were associated with benefits. Estimates of effect sizes were imprecise due to the attained sample size, but our study nevertheless contributes importantly to the total number of TB patients in whom these nutritional adjunctive therapies have now been trialled. These results in our Asian setting are largely supported by the findings from recent studies individually investigating arginine in Africa [30], [31] or vitD in Africa [11] and the UK [10].

We found no interaction between interventions, although our study was insufficiently powered to exclude an interaction. Re-evaluating the sample size for interaction coefficient = 0, our study performed as a 2-arm RCT with the primary endpoint available in 155 people would have 64% power (2-tailed α = 0.05) to show the 20% difference in week 4 culture status which we sought. For both interventions, the estimated 95% CI of the risk difference indicates that an improvement in sputum culture conversion as large as 20% is unlikely. The absence of blood concentrations of 25(OH)D, L-arginine and other parameters, while leaving mechanistic explanations of results open to speculation, does not change overall interpretations of trial findings. A further potential limitation of the study is that loss of mycobacterial viability, due to transit times and potential over-decontamination in the laboratory, could have occurred, thus producing falsely-negative culture results. This would be unlikely to affect randomisation arms differentially, hence would not have biased results, but could have further decreased the sensitivity to detect a true difference between arms. Our study provides a pragmatic assessment of the value, or lack thereof, of vitD &/or L-arginine in high TB-burden tropical settings where such blood tests are generally unavailable.

The largely negative findings among vitD RCTs in active TB to date contrast with results of observational [7] and immunological [4], [5] studies. Reasons may include that ‘vitamin D deficiency’ reported in disease states is non-causal; that supplementation may be beneficial but requires RCTs with greater power or dosing; that the sigmoid dose-response curve for vitD means that only a small band of people towards the centre of the curve will experience substantial effects from a given supplementary dose [47]; or that host determinants of response to vitD (e.g. vitD receptor polymorphisms) require greater consideration [10]. Serum 25(OH)D has been shown to fall during development of TB immune restoration inflammatory syndrome, in inverse proportion to serum cytokines, suggesting that low 25(OH)D occurs in response to immunological activation [48]. Low 25(OH)D frequently observed in TB, which can recover over time without supplementation [11], [49], may thus be a consequence rather than a risk factor for TB.

In a previous vitD RCT [11], 365 PTB patients in Guinea-Bissau were administered 100,000 IU vitD or placebo at 0, 5, 8 months; serum 25(OH)D achieved was not higher in the supplemented than the placebo arm, and their study, as ours, was somewhat underpowered. Despite lack of benefits, further investigation therefore remained warranted. In the UK, Martineau *et al* [10] used a substantially larger early cumulative vitD dose (100,000 IU at 0, 14, 28, 42 days) in a more vitD-deficient population. While the beneficial effect on the primary outcome was not statistically significant overall, *Taq*I vitD receptor genotype status modified the response, such that culture conversion was significantly faster in those with *Taq*I tt genotype randomised to vitD. More recent analyses from this study, restricted to 95 patients in per-protocol analysis, did demonstrate significantly accelerated sputum smear conversion and immunological impacts from supplemental vitamin D [17]. Based on safety concerns when devising our study, and plausible baseline [25(OH)D] in low-latitude Papua, the vitD dose we selected was closer to that used by Wesje *et al* [11]; sub-therapeutic dosing could thus partly explain our negative findings. Because serum 25(OH)D concentration cannot be measured routinely in most TB-endemic settings, any vitamin D intervention for large-scale programmatic roll-out would need to comprise a dose suitable in individuals with a range of baseline vitamin D levels. A consistent result is that vitD has not caused hypercalcaemia in TB, contrasting with previous concerns [50]. More recently, 2 doses of supplementary vitD 600,000 IU administered intramuscularly to 199 Pakistani PTB patients reportedly resulted in significantly greater weight gain (1.14 kg) and greater radiological improvements compared with people who received placebo, but there was no impact on sputum smear clearance rates, and adverse events were not reported [18].

Culture conversion rates appeared lower at weeks 4 and 8 in people randomised to vitD (more so in the HIV- subgroup), and improvement in quality of life (SGRQ score) was lower (Figure 3b), but confidence intervals were wide and HIV+ people were over-represented in the vitD arm. These findings are hypothesis-generating. They are readily explained by the play of chance, arising from having performed many comparisons. Harm has not been attributed to vitD in TB elsewhere [10], [11], [18].

We hypothesised that L-arginine 6 g would increase pulmonary NO production and thereby enhance culture conversion, but this was not observed. In other studies, L-arginine 6 g significantly raised FE_NO_ when given orally to volunteers [51], and intravenously in malaria [52]. However in PTB, L-arginine may be more readily degraded by arginases to ornithine (hence unavailable for conversion to NO), due to the predominance of immune responses favouring arginase production, such as macrophage type 2 (M2) responses. M2 phenotypes are thought to characterise advanced TB [27], [28], increased arginase production is recognised in TB [29], [53], and arginase over-production has been hypothesised as a *M.tb*-induced immune-evasion strategy [54]. Additionally, levels of circulating asymmetric dimethylarginine (ADMA), an endogenous NOS inhibitor, might limit production of NO from L-arginine in TB. Future studies of L-arginine metabolism in TB will be able to address these hypotheses.

In conclusion, neither oral vitamin D nor L-arginine in the doses tested had discernible effects on microbiological or clinical outcomes of PTB. The sample size means that small beneficial effects cannot be excluded. Considered with other recently-published findings, it appears that future studies of vitD in active TB might require higher doses, and targeted participant selection on the basis of serum 25(OH)D and, potentially, host genetic determinants of vitD metabolism. Investigation of supplementary vitD may be more worthwhile in people with latent rather than active TB [55]. L-arginine at higher doses might be capable of measurably increasing pulmonary NO bioavailability, and thereby improving macrophage antimycobacterial activity. However, higher oral L-arginine doses have gastrointestinal adverse effects and dosing (more tablets, more times daily) becomes unwieldy. Testing of other modes of L-arginine delivery (e.g. via inhalation [56]), or of alternative agents increasing NO-bioavailability, and clarification of the immunological mechanisms underpinning vitD and arginine/NO metabolism, merit further investigation.

## Supporting Information

Protocol S1 **Trial protocol.**
(PDF)

Checklist S1 **Supporting CONSORT checklist.**
(DOC)
