# Supplementary material for: L-arginine and Vitamin D Adjunctive Therapies in Pulmonary Tuberculosis: A Randomised, Double-Blind, Placebo-Controlled Trial
Source: PLoS One. 2013 Aug 14;8(8):e70032. doi: 10.1371/journal.pone.0070032 (PMC3743888; doi:10.1371/journal.pone.0070032)
Supplement: Protocol S1 — Trial protocol. (PDF) [file pone.0070032.s001.pdf]

NATIONAL INSTITUTE OF HEALTH RESEARCH
& DEVELOPMENT / MENZIES SCHOOL OF
HEALTH RESEARCH
HEALTH RESEARCH COLLABORATION,
TIMIKA RESEARCH FACILITY

ARGININE AND VITAMIN D IN PULMONARY TUBERCULOSIS

(‘ADAPT’) STUDY PROTOCOL

VERSION 3

FEBRUARY 2008

Incorporating:

- ARGININE & VITAMIN D ADJUVANT THERAPY IN PULMONARY TB

and related sub-studies:

- ECONOMIC ANALYSIS
- EXTRA-PULMONARY TB AND HIV-TB SURVEILLANCE
- DETERMINATION OF NORMAL REFERENCE RANGES
- RAPID DIAGNOSTIC TEST EVALUATION

Investigators:

- **Dr Sandjaja,** PhD, Senior Researcher, Food and Nutrition Research and Development Center, National Institute for Health Research & Development (NIHRD), Bogor, Indonesia: Indonesian PI
- **Prof Nick Anstey** MBBS FRACP PhD, Infectious Diseases Physician and Head, International Health Division, Menzies School of Health Research (MSHR) Darwin: Australian Co-PI
- **A/Prof Paul Kelly,** MBBS, DTM&H, PhD, FAFPHM, Honorary Principal Research Fellow, MSHR & Director, Masters of Applied Epidemiology Program, National Centre for Epidemiology & Population Health, The Australian National University (ANU), Canberra, ACT: Australian Co-PI
- **Drg. Franciscus Thio**, MPPM, Health Services Manager, District Ministry of Health (DinKes), Timika, Papua, Indonesia and PhD Scholar, ANU
- **Dr Anna Ralph**, MBBS, MPH, DTM&H, FRACP, Infectious Diseases Physician and PhD Scholar, ANU and Honorary Research Fellow, MSHR, Australia
- **Dr Dina Bisara Lolong,** MD, MA, Clinical Researcher**,** Biomedical and Pharmaceutical Research and Development Centre, NIHRD, Jakarta
- **Dr Enny Kenangalem** MD, Research Clinician, NIHRD-MSHR Research Program, Timika and Dinas Kesehatan, Timika, Papua, Indonesia
- **Ms. Kristina Retnoningtyas Palupi**, SSi, Laboratory Scientist, NIHRD
- **Dr Emiliana Tjitra,** MD, PhD. Senior researcher, NIHRD
- **Dr Firdy Permana**, MD, B.Med.Sc, M.Sc.PH, Public Health Coordinator, Public Health Malaria Control, Timika, Papua, Indonesia
- **Dr Andri Wiguna,** MD, Clinician, Tuberculosis Clinic, Timika, Papua, Indonesia
- **Dr Enny Malonda**, MD, Tuberculosis clinician, Rumah Sakit Mitra Masyarakat (RSMM), Timika, Papua, Indonesia
- **Bapak Govert Waramori**, Research Nurse, Malaria Control and Public Health Department, PTFI, Timika
- **A/Prof Graham Maguire,** MBBS, PhD, FRACP, Respiratory Physician and Honorary Research Fellow, MSHR
- **A/Prof Peter Morris**, MBBS, PhD, FRACP, Clinical Trials Specialist and Research Fellow, MSHR
- **Dr Ivan Bastian,** MBBS, PhD, FRCPA, Microbiologist, WHO Supranational Reference laboratory, Institute for Medical & Veterinary Science (IMVS), Adelaide, Australia.
- **Dr Pasi Penttinen**, MD, Medical Advisor, Public Health Malaria Control, Timika, Papua, Indonesia.
- **Dr Ric Price**, MBChB, FRCP, PhD, Infectious Diseases Specialist, MSHR

Consultants:

- **Dr Carmelia Basri,** MD, MPH, Director, National Tuberculosis Control Program, Ministry of Health, Jakarta (all aspects of TB Program delivery)
- **Mr Richard Lumb,** BSc, TB Laboratory Scientist, WHO Supranational Reference laboratory, IMVS,IMVS, Adelaide (TB culture and sensitivity)
- **Prof Niels Becker,** BSc, MSc, PhD. Professor of Biostatisics, National Centre for Epidemiology & Population Health, ANU, Canberra, ACT, Australia. (statistical methods).

- **Dr Cheryl Salome,** PhD, Leader, Airway Physiology Group, Woolcock Institute of Medical Research, University of Sydney (exhaled NO measurements and quality control)
- **Prof John Eisman,** BSc (Med), MBBS, FRACP, PhD. Director, Bone and Mineral Research Program, Garvan Institute of Medical Research, Sydney Australia (monitoring effects of Vitamin D)
- **Dr Tonia Woodberry, BSci, PhD,** Laboratory scientist, MSHR (PBMC)
- **Dr Indri Rooslamiati, BPharm,** Laboratory scientist, NIHRD (PK/PD)
- **Prof Stephen Duffull,** MPharm, PhD, Professor of Pharmacy, University of Otago, Dunedin, New Zealand (PK/PD)
- **Dr Tsin Yeo,** MD, PhD Candidate, NIHRD-MSHR Research Collaboration (PK/PD)

Data Safety Monitoring Committee (DSMC):

- **Dr Paulus Sugiarto** MD**,** Chair of Safety monitoring committee. Clinical Director, RSMM, Timika
- **Dwi Hapsari Tjandrarini, Biostatistician, NIHRD, Jakarta.**
- **Dr Louise Maple-Brown,** MBBS FRACP PhD:. Senior Lecturer and Specialist, Internal Medicine and Endocrinology, MSHR
- **Dr Peter Caley**, BSc, PhD, Statistician, ANU.

TABLE OF CONTENTS

INVESTIGATORS: II

GLOSSARY VII

EXECUTIVE SUMMARY 1

1 INTRODUCTION 3

1.1 BACKGROUND 3

1.1.1 Setting and Memorandum of Understanding between NIHRD, RSMM and MSHR 3

1.1.2 Tuberculosis Research outputs from the Timika NIHRD-MSHR Study Site to date 3

1.1.3 Laboratory and Pulmonary Equipment on site 4

1.1.4 Data ownership, management and analysis 4

1.1.5 Postgraduate Training Opportunities 4

1.1.6 Professional Training Opportunities 4

1.1.7 Anticipated Benefits to local medical staff and the wider community 5

1.1.8 Ongoing development of the study protocol 5

1.2 SCIENTIFIC BACKGROUND 6

1.2.1 TB Research Priorities 6

1.2.2 Mechanisms of immune protection against TB in humans 6

1.2.3 Role of NO in mycobacterial killing in TB 7

1.2.4 Vitamin D as an anti-mycobacterial immune mediator 8

1.2.5 Do macrophages kill MTB by predominantly NO- or Vit D-mediated mechanisms? Do the L-arginine-NO and Vitamin D-cathelicidin pathways interact? 10

1.3 PRELIMINARY STUDIES RELATED TO THIS PROPOSAL 11

2 TRIAL HYPOTHESES AND OBJECTIVES 13

2.1 HYPOTHESES 13

2.2 AIMS 13

3 TRIAL METHODS 13

3.1 SETTING 13

3.2 STUDY POPULATION 13

3.2.1 Inclusion criteria 14

3.2.2 Exclusion Criteria 14

3.3 INTERVENTIONS 14

3.3.1 Informed consent 14

3.3.2 Enrolment Procedure 14

3.3.3 Study Medications 15

3.3.4 Participant Evaluations 16

3.3.5 Follow up 17

3.4 TIME LINE 18

4 TRIAL OUTCOME MEASURES 18

4.1 PRIMARY OUTCOME MEASURES 18

4.2 SECONDARY OUTCOME MEASURES 18

5 STATISTICAL ANALYSES 19

5.1 SAMPLE SIZE 19

5.2 OUTCOME ANALYSIS 20

6 ASSESSMENT OF SAFETY 20

6.1 INTERVENTION PROCEDURE 20

6.2 TB MEDICATION SIDE EFFECTS 20

6.3 STOPPING RULES 20

7 RANDOMISATION AND BLINDING 21

8 ETHICAL CONSIDERATIONS ... 21
8.1 RANDOMISED CONTROLLED TRIAL DESIGN ... 21
8.2 POTENTIAL FOR HARM ARISING FROM STUDY MEDICATIONS ... 22
8.3 DISINCENTIVE DUE TO REQUIREMENTS OF STUDY PARTICIPANTS ... 22
8.4 POTENTIAL FOR HARM ARISING FROM BASELINE AND FOLLOW UP MEASUREMENTS ... 22
8.5 FEASIBILITY OF CONTINUATION AFTER STUDY COMPLETION ... 22
8.6 CONFIDENTIALITY AND DATA STORAGE ... 22
9 QUALITY CONTROL AND QUALITY ASSURANCE ... 23
9.1 ELIGIBILITY CRITERIA ... 23
9.2 INFORMED CONSENT PROCESS, INCLUDING REFUSAL RATE AND WITHDRAWAL RATE ... 23
9.3 RANDOMISATION PROCESS ... 23
9.4 ACCURACY OF DATA COLLECTION AND DATA ENTRY ... 23
9.5 ADVERSE EVENTS RELATED TO THE STUDY OR TO TB TREATMENT ... 23
9.6 DATA AND SAFETY MONITORING COMMITTEE ... 23
10 DATA HANDLING AND RECORD KEEPING ... 24
11 FINANCING ... 24
12 PUBLICATION POLICY ... 25
13 APPENDICES A: DETAILS OF STUDY METHODOLOGY ... 28
APPENDIX 1: BASELINE DATA COLLECTION FORM ... 28
APPENDIX 2: WEEKLY DATA COLLECTION FORM ... 35
APPENDIX 3: ADAPT STUDY PARTICIPANT / GUARDIAN INFORMATION SHEET (ENGLISH) ... 42
APPENDIX 3: ADAPT STUDY PARTICIPANT / GUARDIAN INFORMATION SHEET (INDONESIAN) ... 44
APPENDIX 4: ADAPT STUDY PARTICIPANT / GUARDIAN CONSENT FORM (ENGLISH) ... 46
APPENDIX 4: ADAPT STUDY PARTICIPANT / GUARDIAN CONSENT FORM (INDONESIAN) ... 48
APPENDIX 5: RECOGNITION OF ADVERSE EVENTS ... 50
APPENDIX 6: ADVERSE EVENTS MANAGEMENT ... 53
APPENDIX 7: HEALTHY (CONTROL) SUBJECTS ... 62
APPENDIX 8: CONTROL SUBJECT INFORMATION AND CONSENT SHEET (ENGLISH) ... 63
APPENDIX 8: CONTROL SUBJECT INFORMATION AND CONSENT SHEET (INDONESIAN) ... 65
APPENDIX 9: CONTROL SUBJECT DATA COLLECTION FORM ... 67
APPENDIX 10: EXTRAPULMONARY TB SURVEY AND DATA COLLECTION SHEET ... 70
APPENDIX 11: HIV-TB CO-INFECTION ... 71
APPENDIX 11: LUNG FUNCTION TESTING ... 76
APPENDIX 12: SIX MINUTE WALK TEST METHODOLOGY ... 76
APPENDIX 13: EXHALED NITRIC OXIDE MEASUREMENT ... 77
APPENDIX 14: SPUTUM SPECIMEN HANDLING AND PROCESSING ... 78
APPENDIX 15: BLOOD AND URINE HANDLING AND PROCESSING ... 81
APPENDIX 16: ST GEORGE RESPIRATORY QUESTIONNAIRE (ENGLISH VERSION) ... 84
APPENDIX 17: COST-EFFECTIVENESS ANALYSIS ... 87
APPENDIX 18: INVESTIGATOR TEAM ... 89
14 APPENDICES B: MATERIALS TRANSFER ... 90
REFERENCES ... 94

TABLES

Table 1: Baseline and Follow up measurements 17
Table 2: Proposed timeline for ADAPT 18
Table 3: Funding Requirements 24
Table 4: Funding Sources 24
Table 5: Researcher Responsibilities and Publication Plan Proposal 27
Table 6: Summary of Sputum testing to be performed for ADAPT 78
Table 7: Summary of Blood and Urine testing to be performed for ADAPT 81

FIGURES

Figure 1: Diagrammatic Representation of Proposed Tuberculosis Research Programme in Timika 2
Figure 2: Respiratory function and disability in 115 Timika TB patients followed for 12 months........ 12

GLOSSARY

6MWT	6 minute walk test
6MWWD	6 minute weight.walk distance
ADAPT	Arginine and Vitamin D Adjuvant treatment in pulmonary tuberculosis
ANU	Australian National University
ASHM	Australian Society for HIV Medicine
BAL	Bronco-alveolar lavage
BTA	Sputum smear positive
CHATA	Community Health & Tuberculosis Australia (now Australian Respiratory Council)
DLCO	Diffusing capacity of carbon monoxide
DOT	Directly Observed Treatment
DOTS	Directly Observed Treatment, Short-course
DSMC	Data Safety Monitoring Committee
E	Ethambutol
FEV1	Forced expiratory volume in 1 minute
GMP	Good Manufacturing Practice
H	Isoniazid
HIV	Human Immunodeficiency Virus
IFN-γ	Interferon gamma
IMVS	Institute for Medical & Veterinary Science
IUATLD	International Union Against Tuberculosis and Lung Disease
MDR	Multiple drug resistance
MGIT	Mycobacterium Growth Indicator Tube
MIRU	Micro-satellite Interstitial Repetitive Unit typing
MSHR	Menzies School of Health Research (Darwin, Australia)
MTB	*Mycobacterium tuberculosis*
NCEPH	National Centre for Epidemiology & Population Health (Australia)
NHMRC	National Health & Medical Research Council (Australia)
NIHRD	National Institute of Health Research & Development (Indonesia)
NO	Nitric oxide
NOS	Nitric Oxide synthase
NTP	National TB Control Program (Indonesia)
PCR	Polymerase chain reaction
PTB	Pulmonary TB
PTFI	PT Freeport Indonesia
R	Rifampicin
RA	Research assistant
RCT	Randomised, controlled trial
RSMM	Rumah Sakit Mitra Mayarakat (Community hospital in Timika)
S	Streptomycin
SAE	Serious adverse events
TB	Tuberculosis
TNF	Tumour necrosis factor
WHO	World Health Organization
Z	Pyrazinamide

EXECUTIVE SUMMARY

Described herein is our proposed program of related work in the field of tuberculosis research in Timika, Papua Province, Indonesia.

TB, caused by *Mycobacterium tuberculosis* (MTB) is a disease of major public health significance to Indonesia. Our goal is to achieve better treatment outcomes in pulmonary tuberculosis (PTB) through simple and cost-effective interventions. Improvement in TB treatment resulting from successful adjuvant therapy has the potential to decrease duration of infectivity, disability, and duration of antimicrobial treatment. Each would have major clinical and public health benefits. Two major pathways by which human macrophages kill MTB are the arginine-nitric oxide (NO) and Vitamin D pathways. Both NO and activated vitamin D are potent mycobactericidal agents *in vitro.* We therefore propose a clinical RCT to investigate the *in vivo* potency of L-arginine and vitamin D, the substrates for these two major candidate human mycobactericidal pathways, in clearing mycobacteria from sputum and improving clinical outcomes.

A summary of the proposed studies is shown in diagrammatic form in Figure 1. The main study is a randomised controlled trial: 'Arginine or Vitamin D Adjuvant Therapy in Pulmonary Tuberculosis' - the ADAPT Study. In this study we will enrol 444 consecutive newly diagnosed sputum smear positive pulmonary TB patients from two sites in Timika, Papua Province, Indonesia. Patients will be commenced on standard TB treatment and in addition, those who agree will be randomised into four groups (arginine/placebo, vitamin D/placebo, arginine/vitamin D or placebo) and followed for six months. A series of clinical, haematological, microbiological and pulmonary function measurements will be made to assess treatment outcome. The primary outcome measures of sputum culture at 1 month and pulmonary function 2 months after commencing treatment. Related studies include:

1. Economic analysis;
2. Survey of extrapulmonary TB;
3. Evaluation of the burden of TB-HIV co-infection;
4. Evaluation of a rapid diagnostic TB antigen test, and
5. Evaluation of healthy non-TB infected control subjects in order to gain normal reference range values for the RCT outcome measures.

The proposed research program will include significant capacity building opportunities, including 2 PhDs, 1 Masters and a range of professional training opportunities in Indonesia and Australia.

Figure 1: Diagrammatic Representation of Proposed Tuberculosis Research Programme in Timika

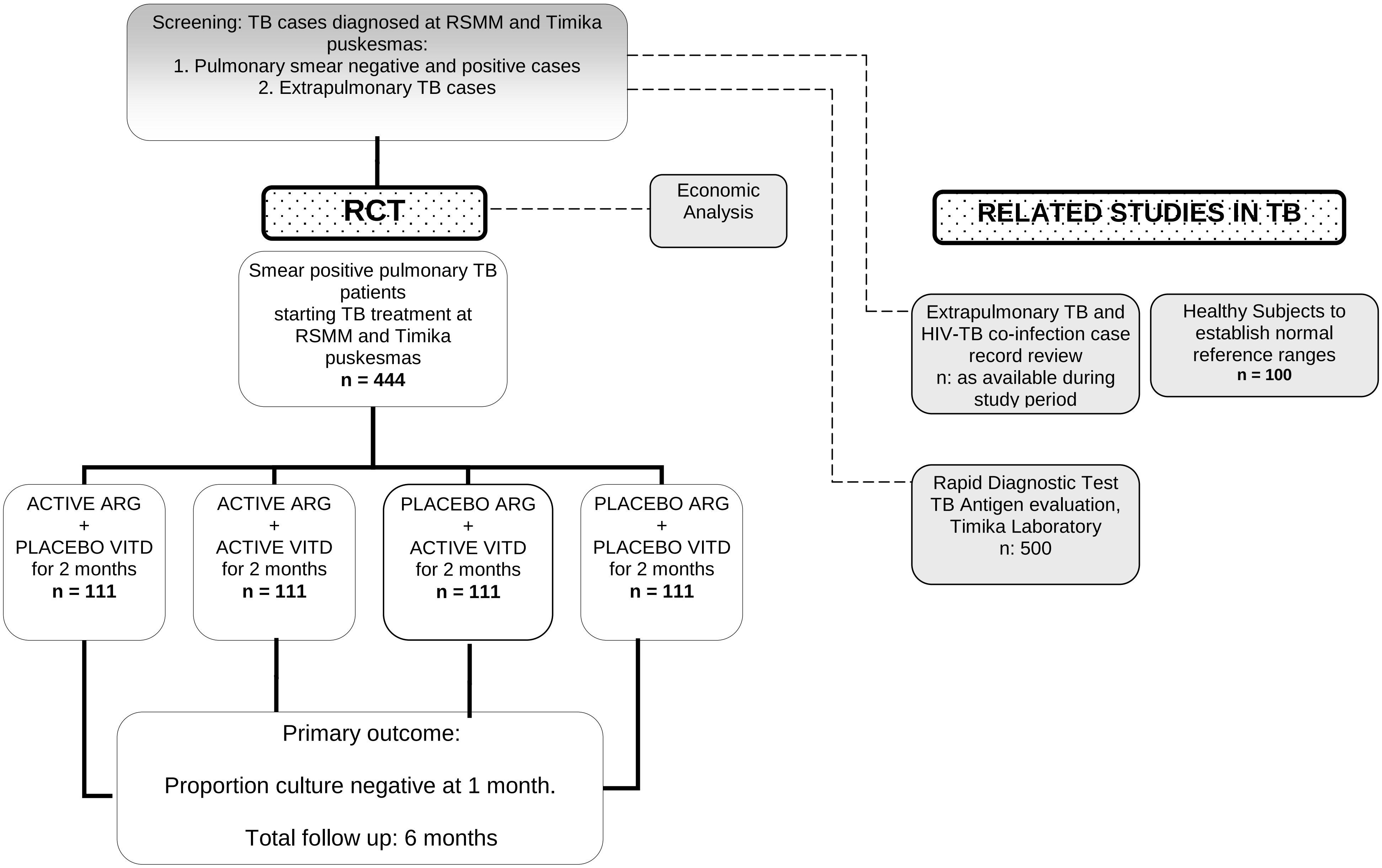

1 INTRODUCTION

1.1 Background

1.1.1 Setting and Memorandum of Understanding between NIHRD, RSMM and MSHR

The study will be based at Rumah Sakit Mitra Masyarakat (RSMM) and at the TB control program (NTP) at Timika Puskesmas, Papua province. It builds on existing research collaborations and Memoranda of Understanding (MOU) between NIHRD, RSMM, Timika District Health Department and MSHR. When the Government to Government agreement for health research cooperation was signed in 1996, and the NIHRD-MSHR MOU in 1998, the agreements specified two priority diseases of public health importance to Indonesia which should form the basis of the collaboration: malaria and tuberculosis (TB). While malaria has been the major focus of our collaborative efforts to date, we have undertaken a number of small studies in TB (see 1.1.2). We are now proposing to extend our initial TB studies in keeping with the MSHR-NIHRD agreement calling for an additional focus on TB-related studies.

1.1.2 Tuberculosis Research outputs from the Timika NIHRD-MSHR Study Site to date

We have undertaken a small series of TB studies at the NIHRD-MSHR study site in Timika as part of a collaboration among personnel from Timika Pusksesmas, Dinas Kesehatan, RSMM, NIHRD, MSHR and Public Health and Malaria Control, PTFI. Whilst these have been small projects, with limited funds, they have achieved good outputs including:

- Funding for Bapak Harun from NIHRD to travel to Adelaide (via MSHR) to undertake TB diagnostic training at the WHO Supranational Reference Laboratory, and supply of reagents and protocols to continue this work at NIHRD;
- Funding for a Papuan co-investigator, Bapak Govert Waramori, to enroll in the Masters of Public Health at MSHR;
- Capacity building in Timika including multiple seminars and on-the-job training in clinical, laboratory and public health interventions to improve TB control in Timika District;
- Two quality assurance visits from the WHO Supranational TB Reference Laboratory with subsequent improvements in TB laboratory performance in Timika Puskesmas and RSMM;
- Information on multi-drug resistant TB (MDR-TB) for the Indonesian National TB Control Program to fulfill their reporting requirements to WHO, and to inform the national drug resistance survey currently underway in several other sites in Indonesia; and
- Five peer reviewed papers (4 published, 1 under review), including 2 papers first authored by Indonesian co-investigators (Dr Muhamed Ardian, Timika Dinas Kesehatan doctor and Dr Tjandra Handojo, staff of the NIHRD-MSHR research collaboration in Timika), and all co-authored with Indonesian investigators.

1.1.3 Laboratory and Pulmonary Equipment on site

A major strength of this study is our existing research base at the proposed field site, established with support from the Wellcome Trust-NHMRC to NIHRD-MSHR. A well-equipped research laboratory, clinical research ward and accommodation block for visiting researchers has been established on the Timika Hospital campus. The NIHRD-MSHR Timika Laboratory has on-site equipment (including the NIOX NO analyser, pulmonary function testing equipment, refrigerated centrifuges, bio-safety cabinet, -70°C freezers, microscopes and a Coulter counter). The Timika Puskesmas has a well stocked and well maintained laboratory (safety cabinet, microscopy facilities, reagents for acid fast staining of sputum samples) and a radiological facility on-site. Well trained technicians are also employed at the Puskesmas. NIHRD-MSHR has on-site staff trained in all the proposed procedures. The research team in Timika is very familiar with RCTs, with randomisation of over 1200 patients in local malaria trials to date (1-4).

1.1.4 Data ownership, management and analysis

The NIHRD and Menzies School of Health Research will have co-ownership of the data, consistent with the Memorandum of Understanding and with previous practice of other collaborative studies between our two institutions.

1.1.5 Postgraduate Training Opportunities

Postgraduate training positions generated by this research include:

1. Drg Frans Thio, Dinas Kesehatan on secondment for PhD studies at ANU, scholarship funded by an AusAID Australian Leadership Award
2. Dr Anna Ralph, Infectious Diseases Specialist, PhD studies at ANU, scholarship funded by the National Health & Medial Research Council
3. Bapak Govert Waramori, Research Nurse, Head of Training and Research, Malaria Control and Public Health, Timika and studying MPH at MSHR/Charles Darwin University

1.1.6 Professional Training Opportunities

Important professional training opportunities which will be made possible through this research collaboration include:

1. Ms Kristina Retnoningtyas Palupi: Laboratory training at the WHO Supranational Reference Laboratory in Adelaide in culture, antibiotic sensitivity testing & molecular typing of Timika sputum samples. In 2008, there is also the possibility of training in Sydney followed by on-site supervision for the laboratory scientist who will be carrying out research evaluating a new TB diagnostic tool.
2. Dr Dina Bisara Lolong: Clinical training in the area of HIV-TB management during supervisory site visits to Timika. Applications will be made for funding to attend the annual 2-day ASHM-IDI Clinical Training in HIV Course in Yogjakarta.

There will continue to be training opportunities in Timika and Darwin for members of the research team, subject to funding. These include clinical, radiological, pulmonary function testing (PFT) and public health training. For example, portable PFT equipment has been brought to RSMM, and two RSMM staff members have been trained in its use by MSHR staff.

1.1.7 Anticipated Benefits to local medical staff and the wider community

Preliminary discussions regarding TB treatment in Timika, undertaken in planning this study, have revealed that medical staff at RSMM and the TB Clinic have particular concerns about poor adherence to treatment by patients, loss to follow up, HIV-TB co-infection, and the emergence of drug-resistant TB. The structures and resources associated with this proposed study will help to address these concerns: in particular, two dedicated TB Research Assistant positions will be created. Anticipated benefits of the study, building on those listed in 1.1.2, are summarised as follows:

- Support for medical staff in implementing the National TB Protocol (NTP):
    - Two dedicated nurses to work as TB Research Assistants
    - Access to research vehicle to assist in follow up of treatment defaulters
    - In-services by NTP and Research staff to provide continuing professional development for local medical and nursing staff on the topic of TB, including highlighting HIV testing and treatment in TB
- Quality assurance practices including examination of all sputum smears at the WHO Supranational TB Reference Laboratory
- TB culture and sensitivity testing to be performed at Supranational TB Reference Laboratory as previously (during 2003-4 studies) to:
    - Provide confirmation of TB diagnosis or otherwise (e.g. rates of non-tuberculous mycobacterial pulmonary infections)
    - Complement the national drug resistance survey currently underway in several other sites in Indonesia by providing ongoing information to NTP and WHO on multi-drug resistant TB (MDR-TB) rates
    - Allow individual patient medication regimens to be tailored if resistant isolate identified
- Measures of Vitamin D status of people with TB and healthy controls will provide important information about vitamin D deficiency rates, recognised globally as an emerging health problem
- Potential benefits related to study intervention: if L-arginine and / or vitamin D are found to be beneficial as we and other researchers propose, this will have major positive impacts on TB outcomes for patients enrolled in the study who receive active intervention. If shown to be beneficial, wider roll-out of these treatments can then be undertaken.

1.1.8 Ongoing development of the study protocol

This study protocol is a dynamic collaboration between NIHRD and MSHR. Significant changes to an earlier draft were implemented in response to recommendations made by Drs Sadjaja and Dina Bisara Lolong, including:

- Change from a 3-arm design to a 4-arm factorial design
- Improved attention to HIV-TB co-infection

Further recommendations will be incorporated where possible.

1.2 Scientific Background

Tuberculosis is the leading cause of death from a curable infectious disease(5). There are an estimated 1.7 billion people (a third of the world's population) infected with TB, 9 million new cases of TB disease each year and an estimated 2 million deaths annually(6). In the Asia-Pacific region, TB represents a major cause of mortality and morbidity and TB treatment consumes a high proportion of health budgets. In 2004, 59% of all TB cases notified to WHO occurred in the Asia-Pacific Region(6). In Indonesia, the TB incidence rate is 245 per 100,000 population per year with a death rate amongst TB patients of 19%(6). This represents 539,000 cases of TB and 100,000 deaths from the disease each year. As most of these cases occur in people in the productive years of life, TB has a major economic impact(7, 8). In response to a resurgence in the disease, WHO declared TB a "global emergency" in 1993(9) and subsequently launched a comprehensive Stop TB Strategy, which includes the promotion of high-quality DOTS (directly observed therapy short-course)(10). Although very effective in achieving microbiological cure if compliance with 6-8 months' therapy is achieved, a major limitation of DOTS is this need for supervised therapy for such a prolonged period. Default from therapy is common(11). Moreover, antimicrobial therapy alone does not prevent the significant residual impairment of lung function at the completion of therapy shown in our longitudinal studies in Timika (see 'preliminary data').

1.2.1 TB Research Priorities

To improve TB treatment outcomes, the UNICEF/UNDP/World Bank/WHO Special Programme on Research and Training in Tropical Diseases has identified six research areas of priority importance(12). Our proposed study specifically targets two of these priorities: i) *Development of new drug and treatment regimens* and ii) *Immunopathogenesis studies including the assessment of adjuvant immunotherapy*. The search for new TB drugs has prioritized the discovery of agents with activity against latent, non-dividing bacilli. Latent bacilli become indifferent to antibiotics, i.e. phenotypically antibiotic resistant(13). Adjuvant immunotherapies which might prevent the switch from actively replicating to dormant forms, which might reverse the dormant state, or which in their own right might provide mycobacteriacidal activity against dormant forms, would be eagerly welcomed. Improvements in TB treatment resulting from successful adjuvant therapy has the potential to improve TB treatment by decreasing the duration of infectivity, duration of illness and disability, residual impairment in lung function and the duration of antimicrobial treatment. Each would have major clinical and public health benefits.

1.2.2 Mechanisms of immune protection against TB in humans

The nature of the immune response to *Mycobacterium tuberculosis* in humans has been extensively reviewed(14, 15). Although complex, the adaptive immune response is characterised by T-cell populations capable of recognising mycobacterial antigens. The clearest demonstration of the importance of T cell function in the protective immune response to TB is the markedly increased risk of reactivation and progressive TB in patients with HIV infection and those on immunosuppressant therapy. Key Th1 cytokines such as IFN-γ activate macrophages to kill most, but not always all, tubercule bacilli, which in murine models is through a predominantly NO-mediated mechanism(16). In human macrophages there is evidence for both NO-mediated killing but also killing via Vitamin-D mediated pathways (see below). Both L-arginine and vitamin D have been proposed as adjuvant TB treatments in humans. L-arginine is the critical substrate for NO synthesis by inducible nitric oxide

synthase (NOS2) in macrophages. Arginine and 25(OH)D_3 are taken up by macrophages and converted to the potent mycobacteriocidal molecules, NO and 1,25$(OH)_2D_3$ respectively.

1.2.3 Role of NO in mycobacterial killing in TB

As reviewed by Nathan(16), "NO is the only molecule known to be produced by mammalian cells that can kill tubercle bacilli *in vitro* with a molar potency comparable to chemotherapy". Other in vitro/rodent evidence for the importance of NO in protection from disease include the following: i) Activated mouse macrophages expressing NOS2 kill MTB in vitro, an effect that is ablated by NOS2 inhibitors; ii) MTB-infected NOS2-/- knockout mice develop fulminant disease; iii) Infected mice treated with NOS2 inhibitors also develop fulminant TB(17, 18). and iv) NOS2 is expressed in infected mouse lung where bacterial growth is minimal. It is clearly not ethical to administer NOS2 inhibitors to infected humans. Evidence for a functionally important role for NOS2/NO in human TB is less well-defined, but is accumulating. Bronchoalveolar lavage (BAL) macrophages obtained from TB patients (but not healthy controls) express significant NOS2(19). This 1996 finding, and our own findings that year of NOS2 expression by peripheral blood monocytes in malaria-exposed children(20), were among the first studies to clearly demonstrate that human macrophages/monocytes are capable of high level NOS2 expression *in vivo*, notwithstanding the difficulties demonstrating such expression *in vitro*. In lung resection studies, NOS2 and nitrotyrosine are expressed in macrophages within granulomas and areas of pneumonitis, suggesting that they may be functionally important in TB(21). Wang et al. showed that pulmonary NO production measured by exhaled NO was significantly higher in TB patients than in controls(22). Importantly, NO production from alveolar macrophages derived from BAL was significantly correlated with exhaled NO in these patients, supporting the approach we will use in this study to use exhaled NO as a non-invasive measure of pulmonary NO production in TB(22).

1.2.3.1 Role of NO in attenuating lung destruction in TB

In TB disease there is evidence that NO and IFN-γ may mediate disease-protection not only from mycobacterial killing but also by anti-inflammatory effects and reduced immunopathology/lung destruction(17, 18, 23). In infected NOS2-/- knockout mice and infected mice treated with NOS2 inhibitors, larger and more destructive lung lesions are seen in early infection, before a significant increase in mycobacterial numbers are seen.(24) NO down-regulates lymphocyte activation, induces apoptosis of activated lymphocytes, and inhibits adherence and transmigration of monocytes, all of which are thought to contribute to granulomatous inflammation and to caseating necrosis, lung destruction and fibrosis. NO also down-regulates TNF production(25), a mediator of caseating necrosis of lung tissue and weight loss in TB. Alveolar macrophage NO production is significantly higher in patients with milder disease than those with more extensive TB, consistent with a disease-protective role for NO(22).

1.2.3.2 Adjuvant L-Arginine treatment in human TB

In the only human RCT to date, Schon et al. showed that low dose L-arginine supplementation (1g daily) in Ethiopian TB patients was safe and well-tolerated(26). There was no significant response in the group with HIV co-infection. In a subgroup analysis of those without HIV infection, there was a modest but significant rise in plasma levels of arginine, a significant improvement in resolution of cough and a non-significant trend to more rapid weight gain and sputum smear conversion in the arginine group. Although arginine was safe and promising, limitations of this study include the small

sample size and post-hoc analysis. Our trial in Timika will occur in a population with a high incidence of TB and a low-prevalence of HIV. It will overcome the deficiencies of the earlier trial by using larger patient numbers, an *a priori* analytical plan and a higher dose of arginine (6g/day), which has been used safely and effectively in clinical trials in other disease states(27, 28). The Ethiopian study(26) did not measure exhaled NO, and could not correlate improvement with pulmonary NO production. In our trial we will determine the relationship between the treatment-induced increases in plasma L-arginine/pulmonary NO and each of the clinical, radiological and physiological measures of TB outcome. We have demonstrated that exhaled NO can be reliably measured in illness at our field site in Timika (see below).

1.2.3.3 NO-independent importance of L-arginine in T cell function.

Over the last 5 years, a crucial NO-independent link between L-arginine and T cell function has emerged. CD3ζ is an essential component of the T cell receptor (TCR), specialized antigen recognition molecules expressed on the surface of all CD4 and CD8 T lymphocytes that initiate signal cascades leading to T cell activation, proliferation and cytokine secretion, including IFN-γ required for macrophage activation and mycobacterial killing. Recent studies have shown that peripheral blood T cells from patients with pulmonary TB have decreased CD3ζ expression which normalises with successful treatment at 4 and 9 months(29). T cells infiltrating MTB-infected tissue also express less CD3ζ(30). Depletion of L-arginine *in vitro* (but not other amino acids) causes reduced CD3ζ expression, impaired T cell signalling and diminished proliferation(31-33). Addition of excess L-arginine *ex vivo* leads to CD3ζ re-expression and recovery of T cell proliferation.(34) These data and the elevated levels of arginase (which metabolises L-arginine) found in peripheral blood monuclear cell lysates from TB patients(29), suggest that reduced L-arginine causes impaired CD3ζ expression and cellular unresponsiveness. Our preliminary data from hypoargininemic patients with falciparum malaria shows significant impairment in CD3ζ expression by activated CD8 T cells compared to arginine-replete controls. To date, there have been no clinical studies in any disease state testing whether L-arginine supplementation can reverse CD3ζ-associated *in-vivo* T cell dysfunction. A novel component of our proposal will determine whether L-arginine therapy can accelerate the restoration of T cell-CD3ζ expression and mycobacterial cellular immune responses and determine the association with clinical recovery.

1.2.4 Vitamin D as an anti-mycobacterial immune mediator

The importance of vitamin D_3 in human host resistance to TB has been suspected since the late-nineteenth century(35-38). Now, expanding recognition of the association between vitamin D deficiency and TB risk, and the pleiotropic immunomodulatory functions of vitamin D_3 (summarised below) provide theoretical bases for this supposition. Vitamin D_3 is generated in the skin on exposure to UVB light and is metabolised in the liver to 25-hydroxyvitamin D_3. This circulating metabolite is converted by the enzyme 1α-hydroxylase to the biologically active steroid hormone, 1,25-dihydroxyvitaminD_3 (1,25$(OH)_2D_3$, synonymous hereafter with Vitamin D_3). Its actions are mediated by ligation with the nuclear vitamin D receptor (VDR), resulting in genomic responses, or alternatively, via more recently identified rapid-response receptors which may be associated with the plasma membrane(39).

1.2.4.1 Vitamin D suppresses MTB growth in cultured human cells

Vitamin D_3 has been shown to exhibit inhibitory effects on MTB in cultured human monocytes(40, 41)

and human macrophages cell lines(42, 43). While this action appeared in one study to be mediated by NO upregulation(43), subsequent studies have not replicated this vitamin D_3–NO interplay. Peripheral blood mononuclear cells from TB-infected people were found by Chang et al. to produce vitamin D_3 more readily, but release less NO than control cells(44). Sly et al. found that Vitamin D_3-induced antimycbacterial activity in human macrophages was not due to NO production but rather, was due to phosphatidylinositol-3-kinase (PI3-K)-mediated superoxide production(41).

1.2.4.2 Vitamin D reverses MTB-induced phagocyte maturation arrest

Antimycobacterial activity of vitamin D_3 is further demonstrated by studies of phagolysosome function. An important mechanism of MTB immune evasion is inhibition of phagocyte maturation. Killing of phagocytosed organisms is usually achieved through a calcium-dependent fusion process between phagosomes and toxic acid-containing lysosomes(45). By inhibiting the fusion step, through inhibition of sphingosine kinase(45), MTB is able to achieve latency in a protected niche. Hmama and colleagues have demonstrated that MTB-induced phagocyte maturation arrest in a human macrophage model is able to be reversed by vitamin D_3, via a PI3-K pathway(46). This finding, and those of Sly et al., implicate PI3-K as an important mediator of the antimycobacterial action of vitamin D_3, and provide an example of non-genomic (VDR independent) signaling of vitamin D_3(41).

1.2.4.3 TLR activation triggers vitamin D-mediated MTB immune response; adequate responses require serum vitamin D sufficiency

In their seminal 2006 *Science* paper, Liu et al. demonstrated that TLR1/2 activation of human macrophages (an MTB-induced process in vivo) upregulated the expression of both the vitamin D receptor and 1α-hydroxylase genes, resulting in increased production of the antimicrobial peptide cathelicidin, with subsequent intracellular killing of MTB(47). Thus a second mechanism of vitamin D_3–mediated mycobacterial killing, in this instance utilizing genomic VDR-mediated responses, has been identified.

The researchers went on to demonstrate that successful induction of antimycobacterial activity through cathelicidin production was reliant upon replete serum vitamin D levels. Serum from ethnic groups (African Americans) at higher risk for TB had lower levels of 25(OH)D_3, and their serum was less efficient in supporting cathelicidin mRNA induction; supplementation of this serum with 25(OH)D_3 restored TLR-induction of cathelicidin mRNA. The findings provide an immunological basis for differential susceptibility among human populations to mycobacterial infection; namely, that vitamin D deficiency appears to portend higher risk of TB disease.

1.2.4.4 Epidemiological and clinical evidence for the importance of vitamin D in TB outcomes

Prior to the antibiotic era, nutritional supplementation (including vitamin D-rich cod liver oil) and sunlight exposure were centrepieces of therapy for TB(48-50). Finsen was awarded the Nobel Prize for Medicine in 1903 for demonstrating the use of light exposure (which acts by upregulating vitamin D production) to treat lupus vulgaris (cutaneous tuberculosis)(51). Deficiency of vitamin D has been demonstrated in people with TB(52), and in ethnic groups recognised to be at higher risk for TB(47), including darkly-pigmented migrants to cooler climates(53). who have elevated risk of development of

active TB post-migration. Vitamin D receptor polymorphisms have been found to be under-represented in pulmonary TB patients(54) (and in lepromatous leprosy)(55), suggesting the importance of vitamin D-mediated pathways in determining TB susceptibility, although meta-analysis of VDR polymorphisms has been inconclusive(56).

Martineau and colleagues(57) identified and reviewed three RCTs and ten prospective case series in which vitamin D was administered to patients with TB; most were conducted in the 1950s, were identified as being of poor quality, and used vitamin D_2. were identified as being of poor quality, and used Ergocalciferol (vitamin D_2), which is known to be less efficacious than cholecalciferol (D_3)(58). Only one study investigated the impact of vitamin D supplementation on TB outcome, and although concluding that vitamin D was beneficial, there were no statistically significant differences in clinical response(59). A more recent trial of vitamin D supplementation in Indonesian pulmonary TB patients appeared to be safe, and suggested more rapid sputum clearance and radiological improvement(60), but the trial was small and the process of randomisation was not described. A larger study with rigorous randomisation is required, such as we propose. Liu et al. and Martineau et al. advocate trials of vitamin D supplementation in patients with TB(47), particularly those with vitamin D insufficiency. A study conducted in Surabaya found that three quarters of patients with TB, and the same proportion of control subjects without TB, had serum vitamin D levels of less than 21 ng/mL (=80 nmol/L)(61), consistent with vitamin D deficiency according to latest recommended reference ranges(62). A recent position statement on vitamin D supplementation emphasised that even in countries with apparent high ambient UV light, vitamin D remains a common and under-diagnosed problem(63).

1.2.5 Do macrophages kill MTB by predominantly NO- or Vit D-mediated mechanisms? Do the L-arginine-NO and Vitamin D-cathelicidin pathways interact?

A major debate is ongoing about the fundamental biology of the mechanisms by which human macrophages kill intracellular pathogens such as MTB(47). While rodent models have identified NO as the central mechanism for mycobacterial killing by macrophages and protection from tissue destruction, Liu et al. have postulated that these NO-mediated mechanisms are more important in nocturnal animals such as mice than in diurnal animals such as humans who are able to synthesise vitamin D_3 in the skin on exposure to UV light. Nathan has argued that the limitations of current *in vitro* and *ex vivo* models for inducing human iNOS and testing its role in mycobacterial killing mean we cannot exclude a major role for NO in humans(64). We have shown that notwithstanding the difficulties in inducing NOS2 in vitro, human monocytes/macrophages are capable of high level NOS2 expression in vivo, and that NO production/PBMC NOS2 expression is associated with protection from infectious disease in humans(20).

There are evident intersections between these two immunological pathways. Firstly, CYP27B1, the gene which encodes 1α-hydroxylase (the enzyme which converts 25-hydroxyvitamin D_3 to the biologically active form of vitamin D_3, 1,25-dihydroxyvitamin D_3) gene, requires an extracellular source of L-arginine for full expression, and is upregulated by NO in an avian macrophage cell line(65-67). Secondly, the antimycobacterial effect of vitamin D may be mediated in part by NO.(43, 68) The inhibitory effect of Vitamin D_3 on MTB in cultured human macrophage cell lines was initially thought to be mediated by NO upregulation, as demonstrated by Rockett and colleagues(43), but subsequent studies did not replicate this finding, as described in Section 1.2.4.1. However more recent work by Martineau and colleagues gives support to Rockett's finding that vitamin D_3, when added to MTB-infected cultured human peripheral blood mononuclear cells, did upregulate NOS2a expression, but the effect was small in comparison with the strong upregulating impact of vitamin D_3 on the antimicrobial

peptide cathelicidin(68), which is emerging as an important mediator of anti-TB immunity in humans(47).

Those on both sides of this deabte acknowledge the difficulty of answering this question with current *in vitro* tools(47, 64). Our clinical trial is designed to determine the potential utility of L-arginine and Vitamin D as adjuvant therapies. An additional benefit of our study will be the comparison of the *in vivo* potency of these two major candidate immune pathways of mycobacterial killing in humans in clearing mycobacteria from sputum and ameliorating pathology. As well as the potential for clinical and public health benefit, this study will contribute to the understanding of the biological mechanisms of disease protection in human TB relative to mouse models.

1.3 Preliminary Studies Related to this Proposal

The NIHRD-MSHR research facility in Timika is well placed to undertake the 'Arginine and Vitamin D Adjuvant Therapy in Pulmonary Tuberculosis' (ADAPT) Study. ADAPT is a logical extension of our current field studies in pulmonary TB and the role of adjuvant L-arginine therapy in infectious diseases. We have completed a study of 115 patients with pulmonary TB in Timika and examined the risk factors for impaired lung function. We have been able to follow a high proportion of patients to six months, demonstrating the feasibility of longitudinal studies of TB patients in Timika(69). Preliminary data from the initial Timika TB studies show that TB patients in Timika have severe disease at diagnosis (as measured by sputum positivity, Xray changes, lung function tests and functional measurements), low rates of HIV co-infection (4%) and MDR-TB (2%) high rates (41%) of malnutrition according to WHO criteria (BMI<18.5kg/m^2) and a default rate of less than 10% (69).

Whilst TB patients show signs of clinical, radiological and anthropometric improvement after two months of treatment, respiratory function remains impaired in a significant number of patients, with over 45% having significant pulmonary restriction at 6 months despite high rates of microbiological cure (Figure 2). Additionally, 31% of patients remain in the malnourished range at 2 months. Adjunctive therapy which accelerates mycobacterial killing, modifies the immunological response to TB and/or facilitates a more rapid return to normal weight in TB patients would be of enormous benefit in this population.

Preliminary results from our trials of arginine supplementation in patients with malaria in Timika have demonstrated substantial increases in exhaled NO: 6g supplementation led to a mean increase of 55%. The immunohistopathological changes associated with TB suggest that NOS2 expression in TB is at least as high as in malaria. Therefore, if the malaria result is replicated in our TB study, 6g of L-arginine would be expected to increase exhaled NO by at least 55%.

Figure 2: Respiratory function and disability in 115 Timika TB patients followed for 12 months.

Top panel: Spirometry: FEV1: forced expiratory volume in 1 second. **Bottom panel:** respiratory disability (modified S George Respiratory Questionnaire score. Score >10 is considered abnormal)

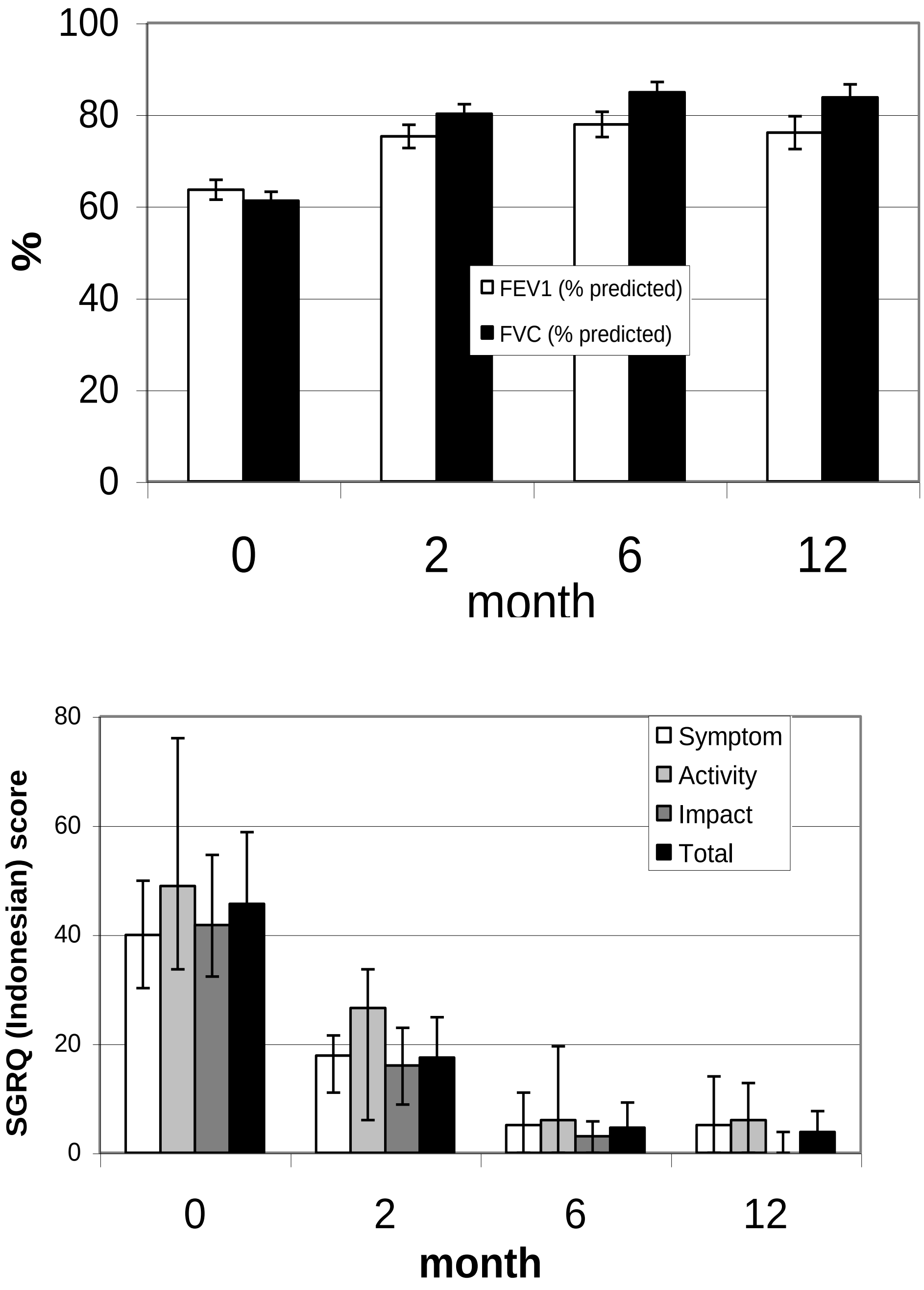

2 TRIAL HYPOTHESES AND OBJECTIVES

2.1 Hypotheses

1) L-arginine supplementation in pulmonary TB will be safe, will increase plasma arginine concentrations, will enhance pulmonary production of nitric oxide (NO) (a key arginine-dependent immunomodulator and downstream immune mediator of mycobacterial killing) and will improve the rapidity and magnitude of the microbiological and clinical response. Baseline pulmonary NO production will be elevated in pulmonary TB but inversely associated with disease severity. Both baseline and post-treatment increments in exhaled NO will be associated with rapidity and magnitude of the treatment response.

2) Supplementation with vitamin D, the metabolite of which (1,25-dihydroxyvitamin D_3) has anti-mycobacterial activity, will be safe, will increase plasma Vitamin D concentrations, and will improve the rapidity and magnitude of the treatment response in human PTB.

2.2 Aims

1) Determine whether supplementation with L-arginine and / or vitamin D is safe and results in more rapid improvement in clinical, mycobacterial, immunological, radiological, physiological and functional measures of treatment outcome. We will randomise patients with pulmonary TB to receive, in addition to standard TB therapy, adjuvant arginine, vitamin D, arginine and vitamin D, or placebo. We will relate serial measurements of plasma concentrations of L-arginine and vitamin D, and immunological responses (pulmonary NO production, T cell function and phenotype) to measures of treatment outcome [mycobacterial (sputum smear clearance and culture conversion), physiological (spirometry), clinical (symptoms and weight), radiological (chest Xray) and functional (six-minute walk test, modified St George Respiratory Questionnaire)].

2) Determine whether expired NO is inversely related to disease severity at presentation. Baseline and serial measures of NO production will be correlated with disease severity and the magnitude and rapidity of clinical response.

3 TRIAL METHODS

3.1 Setting

The study will be based at the Timika TB Clinic and community health centre (Puskesmas) and the community hospital, Rumah Sakit Mitra Masyarakat (RSMM), Timika, Papua Province, Indonesia.

3.2 Study Population

Patients diagnosed with pulmonary TB at RSMM, the Timika TB clinic or the Timika Puskesmas will be eligible to enrol in the study. All patients with suspected TB will be diagnosed in the usual way according to the NTP guidelines. This will include three sputum smears examined for acid fast bacilli and a chest Xray. During the study period, surveillance and evaluation of extrapulmonary TB cases will

also take place (Appendix 10).

3.2.1 Inclusion criteria

Adults 15 years of age or older will be recruited if they have sputum smear positive pulmonary TB; have never received more that one month of anti-TB treatment in the past (that is, only new cases of TB will be included); agree to continue treatment in Timika for the full six month course of treatment; are not pregnant, and consent to enrol in the study.

3.2.2 Exclusion Criteria

Children less than 15 years of age, TB patients who have previously received treatment for TB for more than one month, patients unwilling to continue treatment in Timika for the full course, pregnant patients and those who are not willing to enrol in the study, will be excluded. Patients will be excluded from the study if hypercalcaemia (ionized calcium >1.32 mmol/L) is identified at baseline.

3.3 Interventions

3.3.1 Informed consent

All patients will receive a detailed explanation regarding the reason for the study and what it involves, and regarding the voluntary nature of involvement in the study. Each eligible patient will be provided with verbal and written information in Bahasa Indonesia, or verbally using a Papuan translator if required.

Only patients who understand the study and provide informed consent will be enrolled. It will be explicitly stated that involvement is voluntary, participants are able to say no, and if they do initially consent, they can leave the study at any time they wish without prejudicing their treatment in any way. On withdrawing from the study, the researchers will provide ongoing positive encouragement and support for the participants to continue her/his TB treatment as usual, according to the National TB Protocol.

3.3.2 Enrolment Procedure

All patients with confirmed smear positive pulmonary TB will be provided by the Research Assistant (RA) with verbal and written information in Bahasa Indonesia (see Patient Information Sheet, Appendix 3) regarding ADAPT, and informed consent will be obtained prior to patient enrolment. For participants who cannot read the information sheet and consent form, the study will be carefully and fully explained using a Papuan translator if required. Consenting participants will then receive their randomisation assignation. To achieve this, an envelope will be opened containing the code (Study Identification (ID) Number) assigning the patient to an unidentified (blinded) study arm. The Study ID Number will match a numbered Study Drug Packet containing the study drugs (see also Section 7).

Before any medications are administered, the baseline blood sample will be collected (see below) for immediate calcium results, along with the evaluations listed below (section 3.3.3.1). The first supervised dose of TB medication plus study drugs will then be administered. The patient name, time

of randomisation, the randomisation number, and whether or not they were excluded from the vitamin D/placebo arm will all be noted.

All patients will be required to attend the clinic daily during the intensive phase and twice a month for the continuation phase to receive their anti-TB medications, study medications, routine examinations and the study examinations as outlined below (Follow up, section 3.3.5).

3.3.3 Study Medications

3.3.3.1 Medication administration

At enrolment, patients will be randomly assigned to one of three groups:

1) Group 1: supplementary **L-arginine** 6g daily for eight weeks plus **placebo vitamin D_3** once every four weeks for two doses (0 and 4 weeks);
2) Group 2: supplementary **L-arginine** 6g daily for eight weeks plus **vitamin D_3** (cholecalciferol) 50 000 IU (1250mcg) once every four weeks for two doses (0 and 4 weeks);
3) Group 3: **placebo L-arginine** daily for eight weeks plus **vitamin D_3** (cholecalciferol) 50 000 IU (1250mcg) once every four weeks for two doses (0 and 4 weeks);
4) Group 4: **placebo L-arginine** daily for eight weeks plus **placebo vitamin D_3** every four weeks for two doses (0 and 4 weeks);

Study drugs will be administered separately from the pre-packaged TB medications, provided in standard Indonesian NTP 'Combipacks' (fixed dose combination therapy for TB).

3.3.3.2 L-Arginine

L-arginine hydrochloride powder is commercially available as a GMP standard nutritional supplement (Nutricia Hong Kong). It will be compounded into a 6g dose at RSMM pharmacy. A matching placebo from the same manufacturer with identical appearance, colour, size, and taste will also be prepared.

The study medication will then be packaged and numbered according to the randomisation sequence. Clearly marked Study Drug Packs will be stored in a cool, dry place in the medication cupboards of the Timika TB Clinic and RSMM.

The choice of 6g daily is based on clinical experience with this dose, safety profile at this and higher doses, and the need to adhere to a once daily regimen for TB patients to avoid dosing errors (all other medications are dosed once daily), and to optimise adherence.

3.3.3.3 Vitamin D_3 (Cholecalciferol)

We will use high dose vitamin D_3 (Calciferol Strong, 50 000 IU cholecalciferol; PSM Healthcare, Auckland, NZ), a formulation that allows monthly oral administration. This will be given orally at baseline and again at 4 and 8 weeks. Matching placebo will similarly be obtained. This is similar to but lower dose than standard stoss therapy (intermittent high dose depot vitamin D dosing) used for restoration of vitamin D deficiency(70). The large volume of distribution of vitamin D (since it is fat soluble) necessitates such large doses before any change in plasma vitamin D level is detectable.

3.3.3.4 Cessation of Study Medications

If medications are thought to be causing unacceptable symptoms, the treating doctor will determine the likely pharmacological cause (either of the two study drugs, or any of the four TB drugs) according to the strategy outlined in Appendix 5. If symptoms are determined to be due to arginine, this will be immediately ceased, and reintroduced at a lower dose as outlined in Appendix 5. If symptoms are due to vitamin D/hypercalcaemia, appropriate hypercalcaemia management will be instituted and any further scheduled dose of vitamin D will be cancelled. Any such events will be recorded on the 'Adverse drug events' sheet (Appendix 6) and notified immediately to the study investigators.

3.3.4 Participant Evaluations

3.3.4.1 Clinical, Radiological, Physiological and Functional Evaluations

1) *Height*

Standing height in centimetres will be measured without shoes to the nearest centimetre using a stadiometer, ensuring that the tragus is level with the eyes.

2) *Weight*

Weight in kilograms will be measured without shoes to the nearest 0.1kg using an adult balance.

3) *Body Mass Index (BMI)*

BMI will be calculated in the standard way:

$$BMI = \frac{weight(kg)}{height(m)^2}$$

4) *Questionnaire*

A questionnaire to obtain demographic and clinical information will be completed (see TB Baseline Data Collection form, Appendix 1)

5) *Chest radiograph*

A postero-anterior chest x ray will be performed as per standard clinical protocols at the TB clinic and the RSMM, whether or not the patient is enrolled in the study. Chest Xrays will be evaluated by two study investigators using a previously validated scoring system. The percentage of abnormal lung will be assessed and averaged. The presence or absence of cavitation will also be recorded.

6) *Lung Function*

Respiratory function will be measured as outlined in Appendix 12 using the hand held spirometer (MicroLoop™, Micro Medical, UK) used in our previous studies, which will be calibrated daily. Each patient will perform at least three maximum effort expirations until volumes vary less than 200mls, with the highest values for FVC and FEV_1 used as measures of lung function. Dr Graeme Maguire will supervise the QA/QC of this test.

7) *Exhaled nitric oxide*

Exhaled NO will be used to measure pulmonary NO production at the TB Puskesmas and RSMM, using the online NIOX Mino™ (Aerocrine, Sweden), as detailed in Appendix 13.

.

8) *Modified St George Respiratory Questionnaire (MSGRQ)*

The SGRQ (see Appendix 17) is a valid self-complete measure of health status for patients with lung disease. An overall score and individual domain scores for symptoms, activity and impacts on daily life, are obtained. Patients will be requested to complete the questionnaire without assistance

where possible in order to obtain objective responses, but with assistance from clinic staff if required.

9) 6 minute walk test (6MWT)

The 6 minute walk test will be performed in keeping with standard ATS guidelines as described in Appendix 13. The six minute weight.walk distance (6MWWD), calculated by multiplying this distance in kilometres by the subject's weight in kilograms, will be calculated.

10) Sputum collection

Sputum collections will be obtained at baseline from each TB patient in accordance with NTP standard protocols (minimum of 2 positive smears) prior to enrolment in the study. An additional sputum specimen will be obtained on the day of entry into the study. The study sputum specimen containers will be distinct from standard sterile specimen jars used at the clinic or hospital, and will be labelled with the patient's details (name, study ID number, age, sex), date of collection and laboratory number. A microscopy slide will be prepared from this specimen and examined using standard Zeihl Neelsen method, labelled using a diamond-tipped pen as above and stored at the NIHRD-MSHR Research Building for later QA. Sputum specimens will be divided to provide material for smear examination, antigen testing, culture, susceptibility testing and MIRU typing as detailed in Appendix 15.

11) Blood collection

Twenty mL venous blood (a safe amount) will be collected from participants at enrolment and at follow up weeks 2, 4, 8 and 24 as described in Appendix 15.

12) Urine Collection

A midstream urine specimen will be collected into a sterile specimen container for TB antigen testing as detailed in Appendix 15.

3.3.5 Follow up

Usual follow-up according to the NTP includes monthly weight, repeat chest x ray and sputum at 2 and 6 months, and recording of symptoms, adherence and side-effects. Study participants in addition will receive evaluations as indicated in the table below. Default tracing will comprise three attempts to trace participants who miss two consecutive scheduled clinic appointments, which is standard practice in Timika.

Table 1: Baseline and Follow up measurements

test	week of TB treatment												
	0	1	2	3	4	5	6	7	8	12	16	20	24
weight (weekly)	■	■	■	■	■	■	■	■	■	■	■	■	■
symptoms (weekly)	■	■	■	■	■	■	■	■	■	■	■	■	■
sputum microscopy*	■	■	■	■	■	■	■	■	■				■
sputum culture	■				■				■				
Chest Xray (routine 0,2,6 mths)	■								■				■
expired NO	■		■		■				■				■
spirometry	■				■				■				■
6 minute walk test	■				■				■				■
Respiratory Questionaire (SGRQ)	■				■				■				■
blood													
HIV	■												
25(OH)D3 1,25(OH)2D3 & L-arginine	■		■		■				■				■
iSTAT (including iCa2+, Hb)	■		■		■				■				
T cell CD3 ζ and function	■		■		■								■

Notes:
1. Grey cells indicate that the test will be performed during this week (week 0 = diagnosis, other weeks are follow-up during TB treatment).
2. Routine tests/data collection as part of normal clinical practice in Timika TB clinic/RSMM: weight, symptoms, sputum microscopy (at week 0,8 and 24), chest Xray, HIV
3. Study-related tests/data collection: sputum microscopy (at week 1-7), sputum culture, expired NO, spirometry, 6 minute walk test, respiratory questionnaire and blood tests (vit D, arginine, Ca++ and T-cell function).

3.4 Time line

Table 2: Proposed timeline for ADAPT

Period	Tasks
March 2008-July 09	Recruitment of patients in Timika, lab work commences.
Jul 2009- Oct 2010	Patient follow-up and laboratory work continues.
Oct 2010 – Dec 2010	Completion of laboratory work in Darwin and data analysis
January 2011	Dissemination of results: Timika workshop, report to MoH and publications.

4 TRIAL OUTCOME MEASURES

4.1 Primary outcome measures

1. Proportion of pulmonary TB patients who are culture negative at 1 month
2. Difference in improvement in composite clinical endpoint comprising weight, cough clearance and FEV_1 at 2 months.

4.2 Secondary outcome measures

1. Safety.
2. Difference in improvement in percent predicted FEV_1 at 2 and 6 months.
3. Change in plasma L-arginine and $25(OH)D_3$ concentrations.
4. Weight gain.
5. Cough clearance.

6. Sputum smear conversion time.
7. Functional improvement including Six minute walk test and quality of life assessment using modified St George Respiratory Questionnaire.
8. Immunological improvement (exhaled NO, T cell CD3ζ expression and T cell function).
9. Radiological improvement (percentage lung involvement on CXR at 2 months).
10. Percentage obstructive and/or restrictive lung disease at 6 months.
11. Death, clinical failure and default independently, and 'death *or* clinical failure *or* default'.
12. Primary end points stratified by HIV status.
13. Primary end points stratified by baseline vitamin D and L-arginine status.
14. Primary end points stratified by ethnicity (Papuan and non-Papuan patients).

Use of mycobacterial culture as a primary outcome measure overcomes the potential problem of continued smear positivity of non-viable organisms. Culture at one month will capture any early microbiological effect of adjuvant therapy. As we have shown in previous work in Timika, FEV_1 at 2 months is predictive of longer term deficits in lung function that may be modified by early adjuvant therapy. We will also determine the relationship between baseline and serial measurements of pulmonary NO production, serum L-arginine and 25(OH)D_3 concentrations, and clinical, radiological, microbiological and functional measures of disease severity.

5 STATISTICAL ANALYSES

5.1 Sample size

Sample size has been calculated using the graphical method for 2x2 factorial designs informed by the Fleiss equation, described by Byth and Gebski (71). Making a conservative assumption of sub-additive interaction between the 2 interventions (interaction coefficient of 0.5), then at a level of significance of 5%, a sample size of 444 (111 participants in each arm) will provide 82% power to demonstrate that each treatment results in a 20% reduction in the proportion culture positive at one month (from 60% to 40%), assuming loss to follow up of 10%. As there have been no trials of co-administration of vitamin D and L-arginine, the characteristics of potential immunological interactions can be extrapolated only from *in vitro* macrophage studies, in which findings conflict: vitamin D was found to inhibit iNOS expression in one study(44); yet upregulate *NOS2A* in another (68); the latter study also found the suppressive effect of vitamin D on MTB replication to be partially impaired if NO formation was inhibited. Including a sub-additive interaction term in the sample size calculation provides a margin a caution; if there is minimal or no interaction between the two interventions, our power will be greater. A factorial design rather than a three-arm study allows us to investigate whether if is safe and effective to co-administer the two interventions. Acknowledging the possibility of statistical interaction between the terms, we have an *a priori* plan to analyse primary and secondary outcomes according to subgroups receiving single interventions as well as according to the overall factorial model (71).

Mean time to culture negativity in mostly drug sensitive TB is 32 days (New York) (72) -57 days (Turkey) (73). Based on bacillary burden and extent of cavitatory disease, we estimate a minimum of 60% of Timika patients will be culture positive at 1 month. Our power will be increased if this proportion is higher. This sample size will also be powered to detect a 9% absolute difference in the mean improvement in percent predicted FEV1, a component of the composite endpoint, at 6 months. Mean baseline percent predicted FEV1 in Timika is 63.6% predicted (SD 22) rising to 77.9% predicted (SD 23) at 6 months. Each month, at least 25 eligible smear positive pulmonary TB patients are diagnosed and treated at the Timika study site. If 90% agree to be enrolled, recruitment will take <2yrs to complete.

5.2 Outcome Analysis

The primary analysis of all outcomes will be by intention to treat. All participants in the study will contribute outcomes for analysis whenever possible. Each participant's success or failure for the primary outcome will be determined by an independent observer who will be blinded to the intervention. Analyses will use Intercooled Stata 9.2 (Stata Corp, College Station, Texas, USA). 95% confidence intervals and/or p values <0.05 will be employed to signify statistical significance.

As per recent clinical journal requirements, ADAPT will be registered with the protocol registration system at Clinicaltrials.gov.

6 ASSESSMENT OF SAFETY

6.1 Intervention Procedure

Potential adverse effects of L-arginine and vitamin D will be carefully monitored. Adverse effects from oral L-arginine are uncommon.(27) Use of up to 21g daily was not associated with adverse events in two studies(28, 74). Oral L-arginine 7g three times daily for 4 weeks was associated with diarrhoea in 3 of 27 patients, but this resolved on dose reduction to 7g twice daily(75). Minor gastrointestinal discomfort has been reported in up to 3% of patients(27, 76). We do not expect that this will be a problem but will monitor our study patients carefully for this adverse effect and treat accordingly.

Although much less common than in other granulomatous diseases, vitamin D supplementation in TB has the potential for hypercalcaemia from extra-renal $1,25(OH)_2D_3$ production(77). Based on our previous experience with vitamin D supplementation (Eisman, personal communication), and extensive review of the literature(60, 78-81), the risk of symptomatic hypercalcaemia as a complication of vitamin D supplementation at the dose proposed in this study(63) in patients with pulmonary TB is anticipated to be very low. Ionised calcium will be measured, using portable i-STAT cartridges in Timika, at 0, 2, 4 and 8 weeks. In the unlikely event of hypercalcaemia developing, the literature indicates that the most likely time of onset would be 2-4 weeks post commencement of anti-TB therapy. Patients found to have elevated $i\text{Ca}^{2+}$ on follow up will have their Vit D/placebo dose withheld.

6.2 TB Medication side effects

All patients with pulmonary TB in the study will be treated with anti-tuberculous agents according to the standard NTP protocol. This comprises daily weight-adjusted doses of Rifampicin (R), Isoniazid (H), Pyrazinamide (Z) and Ethambutol (E). Medications are taken under direct observation by health care workers during the intensive phase. Treating doctors will comply with NTP guidelines with regards to standard monitoring for TB drug side effects.

6.3 Stopping rules

Adverse events defined as serious will be i) death and ii) ionised hypercalemia >1.6 mmol/L or hypercalcaemia requiring hospitalisation. All serious adverse events (SAE) will be reported to the chair of the DSMC. It is expected that death may occur during the 6 month treatment and follow up period in up to 5% of patients enrolled in the study related to TB itself (eg advanced/disseminated disease) or co-morbidities (eg AIDS). An interim analysis will be performed after 25 and 50% of participants have

been followed to 2 months, or as requested by the DSMC in the event of SAE related to study drugs. The DSMC will be able to recommend that enrolment into the study ceases if they believe there is evidence that individuals are being harmed through their participation in the study. Early termination of the study (or of one of the study arms) may also be recommended if there is strong evidence of benefit or harm associated with either of the interventions at the interim analysis ($p<0.01$) or if there is strong evidence that further enrolment will not provide any useful information.

7 RANDOMISATION AND BLINDING

A random allocation sequence will be computer-generated (Intercooled Stata 9.2) and concealed from all investigators throughout the study. The random assignment to intervention group will be provided by the MSHR statistician. Block randomisation will be used to maintain similar numbers of participants in the intervention and control groups and to minimise the potential influence of time of enrolment. Allocation will be stratified by place of TB diagnosis (hospital or clinic) and ethnicity (Papuan/non-Papuan), the latter because of the different Vitamin D status of these two groups (as per preliminary data). Allocation concealment will conform to the revised CONSORT guidelines which take account of the logistically challenging environment at the study site (82). We have successfully used this method in over 1,200 malaria patients (three RCTs) in Timika (1, 2, 4). The random allocation will be in sealed, opaque envelopes in two boxes (Papuan and non-Papuan) in each study location. Prior to opening the envelope, the investigator will complete the randomisation form to check if the patient is eligible for enrolment, obtain informed consent and complete the enrolment questionnaire.

Placebo will be compounded to be indistinguishable by appearance, smell and taste from the matching active medication. All study personnel and participants will be blinded to treatment assignment for the duration of the study. Only the study statistician and the Data Safety Monitoring Committee will see any unblinded data and these individuals will not have contact with study participants. The randomisation code will be kept at MSHR by an independent person unrelated to the study. The randomisation sequence will not be broken until primary outcomes have been measured and agreed by the study investigators.

8 ETHICAL CONSIDERATIONS

8.1 Randomised controlled trial design

The perceived withholding of a potentially beneficial medication to half the enrolled study participants may arise as an ethical consideration. To alleviate these concerns, we will explain in advance very clearly to staff, community leaders and patients: (a) the necessity for the blinded, randomised, placebo-controlled approach, and (b) the fact that there is as yet no evidence of benefit of L-arginine and/or vitamin D supplementation in TB, and hence those receiving placebo will not necessarily be disadvantaged. The verbal and written information provided by local research assistants and interpreters will explain the fact that half the patients enrolled will not get the active study drug. Patients will be reminded that they can withdraw from the study at any time without compromising their care or relationship with clinic staff. We have successfully employed blinded RCT design in the local population in large malaria trials to date, with patients willing to participate in such a study design.

All patients, including those who decline to participate in the study, will continue to receive all aspects of standard TB care. They may in fact experience improved care due to greater attention being given to

the importance of TB medication adherence, quality control aspects of the study which will spill over into general practice, and the presence of extra staff (i.e. research assistants and study investigators) being able to provide operational and medical assistance at the clinic.

8.2 Potential for harm arising from study medications

Although anticipated to be unlikely, potential medication side effects will be monitored as detailed in 6.1, 6.2 and Appendices 5 and 6.

8.3 Disincentive due to requirements of study participants

Impacts on participants include the extra medications (active study drug or placebo) and the extra investigations required. The baseline evaluations (standard TB clinic questionnaire, collection of sputum sample, provision of information for informed consent, anthropometric measures, chest radiograph, 6 minute walk, eNO, lung function, modified St George Respiratory Questionnaire) are estimated to take approximately 1 hour per patient. Further appointments of similar duration will be required at 4, 8 and 24 weeks when some of these measures are repeated, but all other follow up visits will be much shorter. It is already routine according to NTP protocols for TB patients undergoing treatment to have a baseline questionnaire, clinical assessment, 3 sputum specimens and chest x ray, then monthly weight, repeat chest x ray and sputum collections at 2 and 6 months, and regular recording of symptoms, adherence and side-effects. The additional time required at the clinic could potentially serve as a disincentive for patients to attend for TB treatment. These extra requirements will be explained at the time of enrolment. In addition, we will be careful to question patients about whether this is the case, offer flexibility regarding time and day of the week on which they can attend, ensure efficiency in carrying out the investigations, and ensure that those who skip some measures, or withdraw altogether, continue to receive their TB therapy.

8.4 Potential for harm arising from baseline and follow up measurements

Measurements will be performed in the purpose-built open-air clinic annex. The filters used with both the spirometry and eNO will prevent transmission of TB. Standard occupational health and safety standards for staff will be upheld, including safe practices in biohazard disposal.

8.5 Feasibility of continuation after study completion

Vitamin D and L-arginine are relatively cheap and readily available and therefore, if shown to be beneficial, would be able to be utilised as adjuvant treatments after completion of the trial at the study site, more widely in Indonesia and potentially in similar settings in other countries.

8.6 Confidentiality and Data storage

All data collected from individuals will be stored confidentially on password-protected computers accessible only to the named investigators, data entry clerks and named research students. Paper records will be stored in locked filing cabinets at the NIHRD-MSHR research building in Timika. We appreciate the risk of loss of data due to computer failure or power blackout and as per our current studies, data will be backed up at least weekly, sent to and stored at the collaborating institutions.

9 QUALITY CONTROL AND QUALITY ASSURANCE

The NIHRD and Menzies School of Health Research will have co-ownership of the data, consistent with the Memorandum of Understanding and with previous practice of other collaborative studies between our two institutions. The research team will be working on the basis of this protocol and the NTP manual. The team from NIHRD and MSHR/ANU will evaluate and assess the work to ensure a high standard procedure applies to this study and ensure the collection of high quality data.

9.1 Eligibility criteria

Dr Anna Ralph will perform weekly checks on all enrolment forms to ensure that the correct diagnostic criteria have been applied and that only patients eligible for inclusion have been included. These will be cross-checked by Dr Dina (NHIRD).

9.2 Informed consent process, including refusal rate and withdrawal rate

The local investigators Dr Firdy and Bapak Govert Waramori will perform weekly checks on all consent forms to ensure that the correct procedures have been followed and that informed consent has been obtained in the manner set out in this protocol.

9.3 Randomisation process

Local investigators will perform weekly checks to ensure that patients have been randomised in the correct manner set out in this protocol. The principal investigators will carry out spot checks on eligibility and the informed consent and randomisation processes during regular visits to Timika.

9.4 Accuracy of data collection and data entry

All data will be double entered and discrepant data will be checked with primary sources. Dr Firdy and Bapak Govert Waramori will carry out spot checks of data collection forms and check the accuracy of information directly from the patient.

9.5 Adverse events related to the study or to TB treatment

Research team members at the TB clinic and at RSMM will report all potential adverse events to the treating doctor and the local investigators (Dr Firdy and Bapak Govert Waramori) immediately. Presumed TB medication side effects will be reported to the treating physician as soon as possible, the strategy outlined in Appendix 5 should be followed, and if necessary, an Adverse Drug Reaction form completed by the treating doctor (Appendix 6). These will be reviewed by Dr Ralph and Dr Dina. Any serious adverse effects will be reported immediately to the principal investigators and the chair of the Data and Safety Monitoring Committee.

9.6 Data and Safety Monitoring Committee

A Data and Safety Monitoring Committee has Indonesian and Australian membership consisting of experienced researchers and health practitioners independent of the research team (NIHRD nominee,

Dr Louise Maple-Brown, Specialist Physician and Endocrinologist, MSHR, Dr Peter Caley, Statistician, ANU and Dr Paulus Sugiarto, Clinical Director, RSMM Hospital, Timika). They will review the progress of the study and give advice to the research team on two occasions during the study. They will have the power to immediately discontinue the study if severe adverse events attributable to the study medications occur.

10 DATA HANDLING AND RECORD KEEPING

Data will be held in Timika for the duration of the trial, paper in a locked filing cabinet and electronic data in a password-protected regularly backed-up file. Back-up copies of data will be regularly sent as required by the relevant ethical committees and for safe-keeping in electronic format to investigators directly involved in this research at NIHRD (Jakarta), MSHR (Darwin) and ANU (Canberra).

The field data collected will be held by the principal investigators for 10 years after publication, in accordance with international best practice requirements and then destroyed.

11 FINANCING

Financing has been secured (subject to ethical approval) from the following sources indicated in Table 4. Funding bodies have indicated that funds need to be spent by the dates indicated. In particular, major in-kind contributions have been committed by the out-going MALKON director, with the expectation of use of funds commencing prior to his departure in June 2008. Pending applications are listed, and further funding applications will be made as appropriate during subsequent years of the TB project.

Table 3: Funding Requirements

**Equipment**	**Cost $ AUD**
e.g. Niox Mino® Unit, Sensorpack and NO cylinder; lung function testing accessories.	15 600
**Study medications**	
Active and placebo L-arginine and vitamin D	11 392
**On-site Specimen Collection And Processing (Timika)**	
Blood, sputum	13 052
**Off-site Specimen Collection And Processing (Australia)**	
e.g. PBMC, 1,25(OH)2D3, PTH, amino acids, sputum	Costs partly absorbed by NHMRC program grant
**Travel and local study site wages**	From 2008 ARC submission
**Study Participant Incentives (travel costs, snack)**	$120 000

Table 4: Funding Sources

Funding Source		Purpose	Time frame for use	Value $ AUD
**1. FUNDING SECURED**				
Australian Respiratory Council 2006 grant		Medications, Sputum, consumables	April 2008	40 000
In-kind support from existing resources at field site (NHMRC Project grant)		Materials		unspecified
Major in-kind contributions pledged by out-going MALKON director.		Materials, travel, in-kind support for clinical staff wages	Pre-June 2008	unspecified
PhD student scholarships and bursaries				
1	*Anna Ralph*			
	Australian National University: Fieldwork Funding Supplement and NCEPH grant	Travel / Materials	2008-2010	11 750
	Endeavour Fellowship	Travel, living costs at Research centre in Timika	2008	20 000
	RACP Covance Fellowship	Material costs	2008	20 000
2	*Frans Thio*			
	AusAID scholarship	Materials and travel	2007-2011	70 000 *****
	Australian National University			To be advised
	Timika local health office			To be advised
**2. APPLICATION / ASSESSMENT PENDING**				
Australian Respiratory Council 2008 grant		Short-listed for 2008 award. To be officially notified February 2008		51 000
National Health and Medical Research Council		Application to be submitted March 2008		To be advised

* includes PhD scholarship and tuition fee

12 PUBLICATION POLICY

The study results will be published by the investigators with acknowledgement of the funding bodies. All co-investigators will be offered authorship according to their involvement in the study and

conforming with standard international definitions for authorship (83). An accurate list of publications to arise from the research cannot be clearly anticipated until research is underway. Possible outputs with identification of assigned researcher and key authors are listed below (Table 5). During the course of the project, there may be changes, which will be agreed upon by mutual agreement of NIHRD & MSHR, to both the list of investigators involved (e.g. in the event of transfer of investigator elsewhere, or requirement to seek further expertise from statisticians or other consultants), and to publications arising.

Research assistants and clinicians at the study site whose work is integral to the research will have the opportunity to be included as authors on publications as appropriate based on international standards of contribution (as in previous publications to date arising from the Timika NIHRD-MSRH research collaboration). NIHRD and MSHR endorse the internationally recognised requirement that first authorship requires leadership of the work involved in each paper (ie considerable ownership and leadership with regard to study design, analysis, writing the paper and major intellectual input to the paper). Co-authorship will apply for all who significantly contributed to the work, in keeping with international standards of authorship (i.e. input to study design and/or data collection and/or analysis as well as a substantial intellectual input to the paper).

Table 5: Researcher Responsibilities and Publication Plan Proposal

Study		Publication topic<br>Each listed topic does not necessarily represent a single publication but may be split into separate manuscripts or amalgamated with others depending on results obtained	Researcher Responsible		Planned main author
			NIHRD	MSHR	
ADAPT	RCT	Validation of a composite clinical endpoint for an assessment of TB severity	Dina<br>Lamria	Anna<br>Paul<br>Nick	Anna
		Correlation of Arg/vit D/NO /other parameters with markers of TB severity: baseline and longitudinal.			
		RCT outcomes and relationships			
		Trends in drug resistance in Papua	Dina		Dina /Paul/ WHO Ref Lab
		TB genotype and ethnicity	Palupi<br>Dina		Palupi /Dina
		T-cell function in TB	Indri	Tonia	Tonia
	Economics	Cost effectiveness of vit D & arg	n/a	Frans	Frans
	Nutrition	Nutritional status of TB patients	Sandjaja & colleagues	Paul	Sandjaja
		Other nutritional topics to be confirmed			
	PK/PD	Arginine pharmacokinetics / pharmacodynamics (embedded into pre-existing study design)	Indri	Steve Duffall	Anna/Indri
EPTB		Diagnosis, ethnicity, treatment outcome & disability	Lamria<br>Dina	Anna<br>Paul<br>Nick	Lumria / Dina
Controls		Vit D/Arg normal values in Papua by ethnicity, nutritional intake & UV	Luxi<br>Sandjaja	Anna<br>Paul<br>Nick	Luxi/Sandjaja
Rapid diagnostic test		Test characteristics on field testing in Papua	Palupi	Anna<br>Paul<br>Nick	Palupi / TBC*
HIV/TB		Descriptive epidemiology including manifestations of HIV-TB (e.g. proportion with EPTB)	Dina	Anna<br>Paul<br>Nick	Dina
		Factors associated with HIV-TB related morbidity			
		Other topics to be developed			
Economic		Active case finding in a high burden country	n/a**	Paul	Frans
		Economic burden of TB in Papua			Frans
		Cost-effectiveness of active vs passive case finding			Frans
Other		Enhanced public/private partnership & standards of TB care in Timika TB control	Dina	Paul	Timika clinician

*TBC: to be confirmed. Negotiation with Proteome Diagnostic company will be required regarding authorship

**n/a = not applicable. Dr Frans is an Indonesian PhD scholar based at ANU.

13 APPENDICES A: Details of study methodology

Appendix A comprises Appendices 1 to 18.

Appendix 1: BASELINE DATA COLLECTION FORM

(inserted hereafter)

ADAPT **Code TAD- |__|__|__|__|**

FORMULIR PENGUMPULAN DATA DASAR

Baseline data collection form / Module P1

Bagain A : Nama Peserta
Section A: Study Participant name

1. Tanggal hari: **|__|__|/|__|__|/20__|__|**
Date today

2. Nama [pertama]: ______________________________________
First Name

3. Nama [keluarga]: ______________________________________
Surname

			Code	
			Papuan	Bukan Papuan
**4. Tempat:** Enrolment Site	TB Clinic	☐1	TAD-1000s	TAD-2000s
	RSMM	☐2	TAD-3000s	TAD-4000s
	Puskesmas	☐3	TAD-5000s	TAD-6000s
	Other	☐4	TAD-7000s	TAD-8000s

Bagain B : Data Pewawancara
Section B: Interviewer's details

5. Nama pewawancara : ______________________________________
Data collector

6. Waktu muliai wawancara : _____/_____
Time interview commenced hr min

7. Waktu selesai wawancara : _____/_____
Time interview ended hr min

Tolong tuliskan no. kartu TB dari respondent yang bersangkutan disetiap halaman formulir ini ***Please write the code number on the participant's TB card and on every page of the data collection form***

PENDAHULUAN

ADAPT **Code TAD- |__|__|__|__|**

Bagain C: Kriteria Inklusi dan Eksklusi

Section C: Inclusion and Exclusion Criteria

8. BTA positif TB paru: Ya ☐1 Tidak ☐2
Smear positive pulmonary TB

9. TB Baru: Ya ☐1 Tidak ☐2
Never treated for TB before

10. Bersedia untuk menjalani pengobatan di Timika selama 6 bulan: Ya ☐1 Tidak ☐2
Agree to continue treatment in Timika for 6 months

11. Umur > 15 th: Ya ☐1 Tidak ☐2
Age >15yrs

12. Persetujuan Consent untuk berpartisipasi dalam penelitian ini: Ya ☐1 Tidak ☐2
Consent given

Bila menjawab "tidak" untuk pertanyaan no. 1-5, pasien tidak bisa dimasukkan dalam penelitian ini.
*If **'No'** to any questions 1 to 5, PATIENT IS NOT ELIGIBLE*

13. Apakah dalam keadaan hamil ? Ya ☐1 Tidak ☐2
Pregnant?

14. Ionized calcium > 1.32 mmol/L? Ya ☐1 Tidak ☐2

Bila pasien menjawab "Ya" untuk pertanyaan no. 6-7, pasien tidak bisa diikutkan dalam penelitian ini.
*If **'Yes'** to question 6-7, PATIENT IS NOT ELIGIBLE*

PENDAHULUAN

ADAPT **Code TAD- |__|__|__|__|**

Bagain D: Informasi dasar peserta
Section D: Respondent Baseline Information

15. Tanggal kelahiran: **|__|__| / |__|__| / 19|__|__|**
Date of birth: dd/mm/yyyy

16. Umur: ____tahun____bulan
Age yr mo

17. Alamat:____
Address

18. Telepon: **|__|__|__|__|__|__|__|__|__|__|**
Phone

19. Jenis Kelamin: Laki ☐1 Perempuan ☐2
Sex: M1,F2

20. Nama Kepala Rumah Tangga:________________________________
Name of head of household

21. Kode rumah tangga: **|__|__|__|__|__|__|__|**
Household code

22. Suku: Papuan ☐1 Bukan Papuan ☐2
Ethnicity

22a. Bila Papuan:
Amungme ☐1
Dani ☐2
Moni ☐3
Damal ☐4
Ekari ☐5
Kamoro ☐6
Nduga ☐7
Other ☐8

22b. Daerah asal:
Origin: h'land1 / l'land2
Daerah pegunungan ☐1
Daerah dataran ☐2

23. Pendidikan terakhir:
Education: (Primary1, High2, Further3)
Sekolah Dasar ☐1
SMP/SMA ☐2
Akademi/Universitas ☐3

24. Riwayat merokok?
Merokok ☐1
Pernah merokok tetapi sudah berhenti ☐2

PENDAHULUAN

ADAPT **Code TAD- |__|__|__|__|**

Smoker? Tidak pernah merokok ☐3

24a. Jika merokok, cigs/day? Berapa banyak rokok yang diisap per hari ? ________ / hari

________ bulan

25. Sudah berapa lama sakit seperti ini?
How long have you have this sickness?

<u>Bagian E</u> Keadaan Perilaku Pengobatan

Section E: Employment and Treatment-seeking behaviour

26. Apakah pekerjaan yang sedang anda kerjakan:
What work would you have been doing:

Bekerja untuk mendapatkan upah *Working for a wage* ☐1
Bekerja di pertanian *Working on a farm* ☐2
Bekerja di rumah *Working at home* ☐3
Pergi ke sekolah *Going to school* ☐4
Berlibur *Leisure time* ☐5
Tidak ada *Nothing* **Jika "Tidak Ada", lanjutkan ke pertanyaan nomor 29** ☐6
Lain-lain, sebutkan *Other, specify* ____________________ ☐7

27. Jika bekerja untuk mendapatkan upah, berapa anda dibayar untuk satu hari bekerja?
How much are you normally paid for one day's work?
Rp._______________

28. Dalam 1 bulan terakhir berapa jumlah hari anda tidak mampu mengerjakan seluruh aktivitas rutin (bekerja / sekolah)?
How many days ware you unable to perform all of your usual activities (work/school)? ________________
hari

29. Dalam 1 bulan terakhir berapa jumlah hari anda hanya mampu mengerjakan sebagian aktivitas rutin (bekerja / sekolah)?
How many days ware you able to perform some of your usual activities (work/school)? ________________
hari

30. Apakah anda mencari pengobatan? Ja ☐1 Tidak ☐2
Did you seek treatment? Yes / No

30a. Jika "Tidak", apa alasannya:
If "No", what is the reason:

PENDAHULUAN

ADAPT **Code TAD- |__|__|__|__|**

Tidak punya biaya untuk berkunjung *No expense to pay a visit* ☐1
Tidak ada sarana transportasi *No transportation* ☐2
Tidak ada biaya transportasi *No transportation expense* ☐3
Obat dan peralatan tidak memadai *Drug and equipments are not be adequate* ☐4
Ketrampilan petugas tidak memadai *Skill worker is not be adequate* ☐5
Pernah mendapatkan pelayanan yang buruk *Have got bad services* ☐6
Penyakit tidak terlalu berat *Disease do not too severe* ☐7
Lainnya, sebutkan *Other, specify* ____________________ ☐8

31. Dimana anda menerima pengobatan dan bagaimana urutannya? Beri nomor sesuai urutan konsultasi
Where did you receive treatment and in what order? Put number in order of consultation

No	**Uraian** *Description*	**Beri nomor sesuai urutan konsultasi** *Put number in order of consultation*	**Berapa kali pasien pergi** *How many times did patient go to*
1	**Pengobatan Tradisional di rumah** *Home traditional remedy*		
2	**Pengobat tradisional** *Traditional healer*		
3	**Obat di rumah atau dari tetangga** *Drugs from home or neighbour*		
4	**Klinik / Dokter Swasta** *Private Clinic / Doctor*		
5	**Apotik** *Pharmacy*		
6	**Toko Obat** *Shop*		
7	**Puskesmas / Pustu** *Public health center*		
8	**Klinik TB** *PHMC Clinic*		
9	**Rumah Sakit** *Hospital*		

Bagain F : Sakit Lain-lain

32. HIV test: Positif ☐1 Negitif ☐2

32a. Bila positif: tanggal diagnosis ___ | ___ | ______
Approximate diagnosis date

32b. Apakah anda minum obat untuk HIV?
Taking medication for HIV?
Ya ☐1 Tidak ☐2

32c. Obat apa? D4T ☐1
What medicines? 3TC ☐2

PENDAHULUAN

ADAPT **Code TAD- |__|__|__|__|**

AZT ☐3
Nevirapine ☐4
Efavirenz ☐5
Other ☐6

33. Are you taking any other medications?

Ya ☐1
Tidak ☐2

33a. Jika ja, obat apa yang digunakan?
What type of medicine?

____

PENDAHULUAN

Appendix 2: WEEKLY DATA COLLECTION FORM

PENDAHULUAN

ADAPT **Code TAD- |__|__|__|__|**

FORMULIR PENGUMPULAN DATA MINGGUAN

Weekly data collection form

Lembar 1 : Data Peserta
Page 1: Participant Identification

1. Nama [pertama]: ______________________________
First Name

2. Nama [keluarga]: ______________________________
surname

3. Alamat baru bila ada perubahan: ______________________________
New Address if changed

4. Tanggal hari: ____ | ____ | 20_____
Date today

5. Minggu pengobatan: (Minggu pendaftaraan = 0; minggu pengobatan 0 – 24) ☐☐
Week of treatment (range: 0-24. Enrolment Week = 0)

Bagain B : Data Pewawancara
Section B: Interviewer's details

6. Nama pengumpul data : ______________________________
Data collector

7. Waktu muliai wawancara : _____/_____
Time interview commenced hr min

8. Waktu selesai wawancara : _____/_____
Time interview ended hr min

MINGGUAN

<u>Bagain C</u>: Tabel Pemeriksaan Mingguan

Section 3: Table of Weekly Testing Required

'X' pada kolom abu-abu berarti pemeriksaan tidak perlu dilakukan dalam minggu tersebut. Pemeriksaan perlu dilakukan pada kolom yang kosong. Lingkari kolom 'pengobatan minggu ke … ' , contoh : minggu 0 = minggu pertama

'X' in grey box means test not required that week. Circle which week of treatment the patient is up to (Week 0 = first week).

**Pengobatan minggu ke …**	**Tanyakan gejala-gejala**	**Kirim dahak untuk pemeriksaan mikro**	**Kirim dahak untuk kultur**	**Ambil darah**	**Periksa NO**	**Periksa Fungsi Paru**	**Tes jalan 6 menit**	**Kuesioner St George**	**Foto Rontgen dada**
Which week?	Ask about symptoms	Send sputum for micro	Send sputum for culture	Collect blood	Meaasure exhaled NO	Do lung function tests	Do 6 minute walk test	Do SGRQ	Do Chest x-ray
0									
1			X	X	X	X	X	X	X
2			X			X	X	X	X
3			X	X	X	X	X	X	X
4									X
5			X	X	X	X	X	X	X
6			X	X	X	X	X	X	X
7			X	X	X	X	X	X	X
8									
12		X	X	X	X	X	X	X	X
16		X	X	X	X	X	X	X	X
20		X	X	X	X	X	X	X	X
24			X						

MINGGUAN

ADAPT **Code : TAD- |__|__|__|__|**

Tolong tuliskan no. kartu TB dari respondent yang bersangkutan disetiap halaman formulir ini

Bagain 4 : Petunjuk Langkah Demi Langkah Pengkajian Mingguan

Section 4: Step-by-step guide to weekly assessments

Instrucsi para pengumpul data

9. Jam berapa makan terakhir ? ____________ **Jam yang lalu**
How long ago did patient last eat? (hrs)

Berikan dosis pengobatan TB dengan supervisi
Give supervised TB medication dose

10. Selama minggu yang lalu, berapa kali minum obat TB?
During the last week, how many doses of TB tablets taken?

- ☐0 0
- ☐1 1
- ☐2 2
- ☐3 3
- ☐4 4
- ☐5 5
- ☐6 6
- ☐7 7
- ☐8 **Don't know**

11. Waktu pengambilan darah ? (dicatat juga di tabung) ____:____ jam
What time was blood taken (write time on tube also)

Minggu 0,2,4,8,24 : ambil darah
Weeks 0,2,4,8,24: take blood

12. Catat hasil pemeriksaan darah iSTAT:
Write iSTAT blood results

Ionised calcium ____________ mmol/L

Creatinine ____________ mmol/L

Haemoglobin ____________ g/dL

13. Jika ada hasil pemeriksaan darah yang relevan:
If any other relevant / abnormal blood tests are available, write results here:

Ingat !! penyimpanan sampel darah
Keep remainder of blood for storage
**** record any adverse events***

Uric acid ____________ mg/dL

ALT ____________ u/L

Other ____________

Other ____________

Other ____________

MINGGUAN

ADAPT **Code : TAD- |__|__|__|__|**

Tolong tuliskan no. kartu TB dari respondent yang bersangkutan disetiap halaman formulir ini

---

14. BTA
AFB

☐1 0
☐2 Scanty
☐3 1+
☐4 2+
☐5 3+
☐6 4+

Setiap minggu sampai minggu ke 8, dan minggu ke 24, Pemeriksaan dahak mikroskopis
Every week until week 8, then week 24, collect sputum for microscopy and indicate result

15. eNO ____:____ jam __________ ppb

Minggu 0 , 2 ,4, 8, 24: Periksa NO dan tulis hasil

16. Selama minggu lalu, berapa kali minum obat sirup
During the last week, how many doses of study medication were taken?

☐0 0
☐1 1
☐2 2
☐3 3
☐4 4
☐5 5
☐6 6
☐7 7
☐8 **Don't know**

Berikan makanan ringan kepada pasien
Give patient a snack
Berikan pengobatan studi (obat sirup setiap minggu mulai minggu 0 – 8, obat tablet hanya pada minggu 0 dan 4)
Give study medications (arg every week, D only week 0 and 4)

17. Berat badan __________ kg

18. Height __________ m

Omit if already done in previous week and not growing

19. Tes jalan 6 menit __________ m
6-min walk test

MINGGU 0,4,8,24: Lakukan tes jalan 6 menit dan catat jarak tempuh jalan:
Weeks 0,4,8,24 : Do 6-min walk test

20. Tes fungsi paru
Lung function tests

Lakukan tes fungsi paru dan catat hasil

FEV1 __________ L/s

FVC __________ L

21. Kuisioner Respiratori St George Ya ☐1 Tidak ☐2
SGRQ done yes / no

MINGGU 0,4,8,24: Interview and isi kuisioner Respiratori St George

22. Foto rontgen Ya ☐1 Tidak ☐2

MINGGU 0,8, 24: CXR

MINGGUAN

ADAPT **Code : TAD- |__|__|__|__|**

Tolong tuliskan no. kartu TB dari respondent yang bersangkutan disetiap halaman formulir ini

---

23. Symptoms

SETIAP MINGGU : Tanyakan tanda-tanda yang nampak
Symptoms (every week)
**** record any adverse events***

**Batuk** Cough	Ya ☐1	Tidak ☐2	
**- Jika ada batuk:** If cough present:	☐1	Ringan	Mild
	☐2	Sedang	Moderate
	☐3	Parah	Severe
	☐4	Parah sekali	Very severe
**- Apakah ada perubahan?** Has it changed?	☐1	Memburuk	Worse
	☐2	Sama	Same
	☐3	Lebih baik	Better
	☐4	Hilang	Gone
**- Apakah ada lendir?** Sputum?	Ya ☐1	Tidak ☐2	
**- Apakah batuk darah?** Haemoptysis?	Ya ☐1	Tidak ☐2	
**Demam** Fever	Ya ☐1	Tidak ☐2	
**Lemas** Malaise	Ya ☐1	Tidak ☐2	
**Bingung** Confusion	Ya ☐1	Tidak ☐2	
**Kembung** Bloating	Ya ☐1	Tidak ☐2	
**Konstipasi** Constipation	Ya ☐1	Tidak ☐2	
**Mual** Nausea	Ya ☐1	Tidak ☐2	
**Muntah** Vomiting	Ya ☐1	Tidak ☐2	
**Diare** Diarrhoea	Ya ☐1	Tidak ☐2	
**Sakit kepala** Headache	Ya ☐1	Tidak ☐2	
**Jaundice**	Ya ☐1	Tidak ☐2	
**Kemerahan** Rash	Ya ☐1	Tidak ☐2	
**Rasa terbakar, mati rasa di tangan & kaki** Burning, numbness in feet or hands	Ya ☐1	Tidak ☐2	

MINGGUAN

ADAPT **Code : TAD- |__|__|__|__|**

Tolong tuliskan no. kartu TB dari respondent yang bersangkutan disetiap halaman formulir ini

---

**Gg. Pendengaran**<br>Deafness	Ya ☐1	Tidak ☐2
**Gangguan penglihatan**<br>Poor vision, colour blind	Ya ☐1	Tidak ☐2
**Gangguan persendian**<br>Joint pain/ swelling	Ya ☐1	Tidak ☐2
**Berat badan turun**<br>Weight loss	Ya ☐1	Tidak ☐2
**Lain-lain, sebutkan** ___________________<br>Other, specify	Ya ☐1	Tidak ☐2
**Lain-lain, sebutkan** ___________________<br>Other, specify	Ya ☐1	Tidak ☐2

Bagain 5 : Pendapatan, Biaya Perawatan Kesehatan Dan Biaya Perjalanan

Part 5: Income, Health care and travel costs

24. Haruskah anda mengeluarkan uang untuk datang ke klinik pada hari ini ? Ya ☐1 Tidak ☐2
Did you spend money to come to the clinic / hopsital today?

Jika ya:

**24a. Transportasi**<br>Transport	____________	**Rp**
**24b. Makanan & minuman**<br>Food or drink	____________	**Rp**
**24c. Waktu kerja terpotong**<br>Time off work	____________	**Rp**
**24d. Biayan pengobatan**<br>Medical expenses	____________	**Rp**

25. Berapa jarak rumah /tempat tinggal anda dari klinik? ____________ **km**
How far is your home from the clnic / hospital?

26. Bagaimana anda bisa sampai di klinik ini ?
How did you get to the clinic / hospital?

☐1 **Jalan kaki Kendaraan** Walk
☐2 **pribadi** Private car
☐3 **Angkutan umum** Public transport
☐4 **Lain-lain** other

27. Berapa lama waktu yang dibutuhkan untuk sampai di klinik? ____________ **jam**

28. Berapa lama waktu yang dibutuhkan untuk pemeriksaan (termasuk menunggu untuk diperiksa, pemeriksaan dokter dan pemeriksaan lain , menunggu obat)? ____________ **jam**

Apakah anda didampingi oleh seseorang ke klinik ? Ya ☐1 Tidak ☐2

28a. Jika “ya” berapa jumlah uang yang diperoleh pendamping untuk satu jam? __________ Rp

Please remember to write time interview ended on cover page

MINGGUAN

Appendix 3: ADAPT Study Participant / Guardian information sheet (English)

ADAPT Study Participant / Guardian Information sheet

Arginine and Vitamin D Adjuvant Therapies in Pulmonary Tuberculosis (ADAPT)
Research study at Timika Puskesmas and RS Mitra Masyarakat

Study Participant / Guardian information sheet
Can extra medicine help make TB better more quickly?

<u>This is for you to keep</u>

Tuberculosis (TB) is a major cause of sickness in Indonesia. It can cause cough, weight loss and other problems. People with TB need to take antibiotics for a long time, usually 6 months, to be cured. We want to find out if new extra treatments taken with the antibiotics might help patients with TB to get better faster. We are asking people with lung TB to take part in this study. If you agree, we will ask you to take extra tablets for 2 months in addition to the usual TB antibiotics (which you take for 6 months). We will also ask you to do some extra lung and blood tests today, and again in 2, 4, 8 and 24 weeks.

The body needs nutrition, such as protein and vitamins, to stay healthy and help fight off infection. There is a substance all people need called **arginine** which is found in foods like nuts, but also comes in a tablet. Also, an important vitamin is **Vitamin D** which comes from sunshine through the skin, and also from some foods, and can be taken as a tablet too. Some doctors think that extra arginine and vitamin D may be good for people with TB, but there is no clear answer to this yet.

You can help us try to answer this important question. If you agree to be part of this study, you will be given some extra tablets. Some people will receive tablets that have the real arginine or vitamin D, and some people will be given tablets that contain nothing except powder (‘placebo’ tablet). You, your doctor and the study investigators are not allowed to know until the end which you are taking, to make sure the results are not biased. If you are taking the real arginine and / or vitamin D, they should not cause you any harm. It is very uncommon, but some people get stomach upset from arginine, so we will be checking for this and advise to take tablets after food. Vitamin D can rarely make the calcium in blood high, so we will be checking for this in your blood tests. If you have high calcium already, or get high calcium later, we will stop the extra medications.

Whether or not you agree to participate in this study, you will receive the standard TB antibiotics that the health centre usually uses to treat people with tuberculosis. As part of normal care, the TB clinic will ask you to provide a sputum sample, have your height and weight measured, get a chest x ray and have blood taken (including an HIV test) and you will be asked questions about your health.

If you say yes to the study, then when the blood is taken, we will collect an additional 20 ml of blood (4 teaspoons). This will include testing for HIV if you agree, which is standard care for all people with TB. Then we will also ask you to do breathing tests, a walking test for 6 minutes, and answer some more questions about your breathing. When your blood test results are ready, you will get the extra medications. For the lung tests, we will ask you to breathe into the mouthpiece of a machine called a spirometer and a NIOX machine. This does not hurt or cause any harm, but tells us how your lungs are working. As part of

routine care by the TB clinic, every week you will have a check up to see how you are feeling and check your weight. If you are in the study, you will also be asked to do the following tests: sputum test every week; NIOX lung test and blood test in 2, 4, 8 and 24 weeks; chest x ray, spirometer lung test, walk test and questionnaire in 4, 8 and 24 weeks.

Taking part in this study is voluntary; it does not cost you any money. All information collected is confidential. Results will be given to your doctor if they can help the doctor to treat your illness. No personal identification will be revealed to persons outside the study. You do not have to participate if you don't want to, this will not affect your medical treatment, If you decide to participate, you can withdraw from this study at any time for any reason and still receive standard treatment for tuberculosis by the staff at the health centre.

If you have any questions about the study you can telephone Dr Paulus Sugiarto at RS Mitra Masyarakat Hospital on 901-301 881. If you have any concerns or complaints about the conduct of the study, please contact Dr Sandjaja, Chair of the NIHRD Ethics Committee in Jakarta on 081319304040 or 6202104261088, or The Secretary, Human Research Ethics Committee of the NT Department of Health & Community services & Menzies School of Health Research in Australia on 61-8-8922 7922.

Appendix 3: ADAPT Study Participant / Guardian information sheet (Indonesian)

Surat Informasi Peserta Studi ADAPT / Wali

Pengobatan Tambahan Arginin dan Vitamin D terhadap Tuberculosis (TB) Paru-Paru

(ADAPT : Arginine and Vitamin D Adjuvant Therapies in Pulmonary Tuberculosis)

Penyelidikan di Puskesmas Timika dan RS Mitra Masyrarakat

Surat Informasi Peserta / Wali

Apakah pengobatan tambahan dapat membantu menyembuhkan TB secara lebih cepat?

Salinan ini bagi Anda

TB adalah sebab utama penyakit di Indonesia. Batuk, keturunan berat badan, dan gejala lain dapat disebabkan TB. Penderita memerlukan diobati agak lama dengan antbiotika, biasanya selama 6 bulan, sampai menjadi sembuh. Kami ingin mengetahui kalau pengobatan tambahan baru, yang berjalan bersama-sama dengan pengobatan antibiotika tersebut, dapat membantu penderita TB menjadi sembuh secara lebih cepat. Penderita TB paru-paru diundang mengikuti penyelidikan ini. Jika Anda setuju, kami meminta Anda berobat tablet tambahan selama 2 bulan serta antibiotika TB biasa (yang selama 6 bulan). Anda juga diminta mengikuti pemeriksaan tambahan hari ini terhadap paru-paru dan darah, dan juga sesudah 2, 4, 8 dan 24 minggu lagi.

Badan kita memerlukan bahan gizi, misalnya protein dan vitamin, untuk tetap sehat dan membantu dalam pelawanan infeksi. Ada bahan diperlukan semua orang yang disebut **arginin**. Bahan itu terdapat di makanan seperti kacang, dan juga terdapat dalam tablet. Juga, **vitamin D** adalah vitamin penting yang terdapat sebagai hasil perkenaan sinar matahari dengan kulit, dan juga terdapat di beberapa jenis makanan, serta dapat diobati dalam bentuk tablet. Beberapa dokter berpikir mungkin arginin dan vitamin D tambahan dapat memanfaati penderita TB, tetapi belum ada jawaban yang jelas.

Anda bisa membantu kita menjawab pertanyaan yang penting itu. Jika Anda setuju dengan menjadi terlibat dalam studi ini, akan Anda diberi tablet tambahan. Sebagian orang akan diberi tablet yang kosong, hanya berisi bubuk (tablet *placebo*). Anda, dokter Anda dan penyelidik-penyelidik studi ini tidak diberitahu tablet mana yang Anda terima sampai pada saat terakhir, untuk memastikan hasil studi ini tidak mempunyai prasangka. Jika Anda diobati arginin atau vitamin D yang betul, kemungkinan tablet itu membahayakan Anda sangat kecil. Jarang sekali arginin dapat menyebabkan sakit perut. Oleh karena itu kami akan mengawasinya, dan Anda dinasehatkan supaya mengambil tablet sesudah makan. Jarang sekali juga, vitamin D dapat menaikkan kwantitas kalsium dalam darah, sehingga tingkat kalsium akan diperiksa dalam pemeriksaan darah Anda. Kalau Anda sudah

mempunyai tingkat kalsium yang tinggi, atau nanti mengalami tingkat yang tinggi, obat tambahan akan diperhentikan.

Kalau ingin atau tidak ingin mengikuti studi ini, Anda tetap akan menerima antibiotika standar yang biasanya diberi oleh puskesmas untuk mengobati penderita TB. Sebagai pengobatan biasa, Anda diminta klinik TB untuk memberi sampel air ludah, tinggi dan berat badan Anda diukur, dada diperiksa dengan sinar X, pemeriksaan darah dilaksanakan (termasuk pemeriksaan HIV), dan dipertanyakan kesehatan Anda.

Jika Anda menjawab 'ya' untuk mengikuti studi ini, pada waktu sampel darah sudah diambil, lalu kami mengambil 20 ml darah lagi (4 sendok teh). Kami juga meminta Anda mengikuti pemeriksaan pernafasan, berjalan kaki selama 6 menit, dan menjawab beberapa pertanyaan lagi tentang pernafasan Anda. Pada waktu hasil pemeriksaan darah sudah siap, Anda diberi obat-obat tambahan. Dalam pemeriksaan paru-paru, Anda diminta meniup ke dalam mesin yang bernama spirometer dan juga ke dalam mesin NIOX. Kedua mesin tersebut tidak menyakitkan atau membahayakan Anda, hanya menunjukkan bagaimana laku nafas paru-paru. Sebagai pengobatan biasa dari klinik TB, setiap minggu Anda mengikuti pemeriksaan biasa dan berat badan diperiksa. Jika Anda mengikuti studi ini, Anda juga diminta mengikuti pemeriksaan yang berikut: pemeriksaan air ludah setiap minggu; sinar X dada, pemeriksaan paru NIOX dan pemeriksaan darah sesudah 2, 4, 8 dan 24 minggu; pemeriksaan paru spirometer, pemeriksaan jalan kaki dan daftar pertanyaan dijawab sesudah 4, 8 dan 24 minggu.

Pengikutsertaan dalam studi ini dengan sukarela saja, dan tidak ada ongkos kepada Anda. Semua informasi yang dikumpulkan tetap menjadi rahasia. Hasil-hasil pemeriksaan akan diberi kepada dokter Anda hanya kalau membantu dokter mengobati penyakit Anda. Tidak ada informasi pribadi apapun yang dibuka kepada orang dari luar studi ini. Anda tidak harus mengikuti studi ini, kalau begitu pengobatan Anda tetap dilakukan seperti biasa. Jika Anda memutuskan mengikuti studi ini, Anda boleh menarik diri kapan saja dan tetap menerima pengobatan TB yang standar di puskesmas.

Kalau ada pertanyaan mengenai studi ini silahkan Anda menelepon Dr Paulus Sugiarto di RS Mitra Masyarakat dengan nomor 901 301 881. Kalau ada pengabuan tentang pelaksanaan studi, silahkan menghubungi Dr Sandjaja, Ketua Komite Etika NIHRD di Jakarta dengan nomor telepon 081319304040 or 6202104261088,, atau Sekretaris, *Human Research Ethics Committee of the NT Department of Health & Community Services and Menzies School of Health Research* di Australia dengan nomor 62 8 8922 7922.

Appendix 4: ADAPT Study Participant / Guardian consent form (English)

ADAPT Study Participant / Guardian Consent Form

Can extra medicine help make TB better more quickly?

This form means you can say No.

I have read the Patient Information Sheet and have had the details explained to me by the witness below. I understand that I will receive the usual treatment and tests for tuberculosis. I understand that I will also have extra tablets which may be active arginine and / or vitamin D, or placebo. I will also have extra lung tests, blood tests, sputum tests, repeat chest X-rays and questions that are not needed by my doctors to treat my tuberculosis. The extra tablets are being tested to find out if they will help people with TB to get better faster. They are very unlikely to be harmful, but could potentially cause stomach upset or high calcium level. The extra tests will provide information about how my lungs and blood are working. I may not receive any direct benefit from this study, but the results will help answer questions about TB treatment, and therefore may be of help to other people in the future.

I understand that I have tuberculosis and I will be asked to come to Timika TB Clinic / RSMM *(delete one)* for repeat breathing tests, blood tests, chest X-ray, walk test and questionnaire in 2, 4, 8 and 24 weeks. I understand that this will take longer than a usual clinic appointment. I also understand that my medical records at the health centre will be read by research staff and some information about my health will be collected.

I understand that all information collected is confidential and no information will be available to anyone outside the study. Results will be given to my doctor if they can help the doctor to treat my illness. I understand that I do not have to participate in this study. I can withdraw from this study at any time for any reason and still receive standard treatment for tuberculosis by the staff at Rumah Sakit Mitra Masyarakat or Timika TB Clinic.

PARTICIPANT

I (print name)

………………………………………………………………… agree to take part in this study.

Signed Date

………………………………………………………………… ______ / _______ / 20_____

PARTICIPANTS AGED <18 YEARS: PARENT / GUARDIAN TO SIGN

I (print name) agree for my child / person in my care to take part in this study.

…………………………………………………………………

Participant name

…………………………………………………………………

Signed Date

………………………………………………………………… ______ / _______ / 20_____

WITNESS

I (print name) have explained the study & information sheet

…………………………………………………………………

Signed Date

………………………………………………………………… ______ / _______ / 20_____

Appendix 4: ADAPT Study Participant / Guardian consent form (Indonesian)

Formulir Persetujuan ADAPT

Menyelidiki peran pengobatan tambahan dalam menyembuhkan TB secara lebih cepat.

Anda boleh mengatakan Tidak.

Saya sudah membaca Surat Informasi Peserta dan sudah menerima penjelasan perincian surat itu dari saksi yang namanya tertulis dibawah. Saya memahami saya akan menerima pengobatan dan pemeriksaan TB yang standar, dan juga diobati tablet tambahan yang mungkin berisi arginin dan/atau vitamin D, atau kosong (placebo). Saya juga mengerti saya akan mengikuti pemeriksaan paru-paru, darah dan air lubah yang tambahan, serta sinar X dada berulang-ulang dan pertanyaan-pertanyaan yang tidak diperlukan dokter saya untuk mengobati TB saya. Tablet tambahan tersebut diperiksa supaya mengetahui kalau obat itu membantu penderita TB menjadi sembuh secara lebih cepat. Kemungkinan tablet tersebut berbahaya sangat rendah, tetapi kadang mungkin menyebabkan sakit perut atau tingkat kalsium yang tinggi. Pemeriksaan tambahan akan memberi informasi mengenai bagaimana keadaan paru-paru dan darah saya. Mungkin studi ini tidak akan bermanfaat langsung bagi saya, tetapi hasilnya akan membantu menjawab pertanyaan mengenai pengobatan TB, sehingga mungkin dapat memanfaati orang-orang lain pada masa depan.

Saya mengetahui saya sudah terinfeksi TB dan akan diminta mendatangi klinik TB Timika / RS Mitra Masyarakat (hapuskan satu) untuk pemeriksaan pernafasan dan darah berulang-ulang, sinar X dada, pemeriksaan jalan kaki dan menjawab daftar pertanyaan sesudah 2, 4, 8 and 24 minggu. Saya memahami semua itu menghabiskan waktu lebih lama daripada waktu kunjungan klinik biasa. Saya juga mengerti catatan kedokteran saya di puskesmas akan dibaca oleh staf peyelidikan dan sebagian informasi mengenai kesehatan saya akan dikumpulkan.

Saya memahami semua informasi yang dikumpulkan tetap menjadi rahasia dan tidak ada informasi pribadi apapun yang dibuka kepada orang dari luar studi ini. Hasil-hasil hanya diberi kepada dokter saya kalau dapat membantu dokter mengobati penyakit saya. Saya mengerti saya tidak harus mengikuti studi ini, dan boleh menarik diri kapan saja dengan sebab apapun, dan tetap menerima pengobatan TB yang standar dari staf di klinik TB Timika atau RS Mitra Masyarakat.

PESERTA

Saya (tuliskan nama)

.... setuju dengan mengikuti studi ini.

Tanda tangan Tanggal

.... ______ / ______ / 20_____

PESERTA YANG BERUMUR <18 TAHUN: DITANDATANGANI ORANG TUA / WALI

Saya (tuliskan nama)

.... menyetujui anak saya/orang dibawah asuhan saya mengikuti studi ini.

Nama peserta

....

Tanda tangan Tanggal

.... ______ / ______ / 20_____

SAKSI

Saya (tuliskan nama) sudah menjelaskan studi dan

.... surat informasi.

Tanda tangan Tanggal

.... ______ / ______ / 20_____

Appendix 5: Recognition of Adverse Events

Management of Adverse Events

Definitions

Adverse Event (AE): any untoward medical occurrence in a clinical trial subject administered a pharmaceutical product, not necessarily related to the study drugs or TB drugs. FILL IN ADVERSE EVENTS REPOTING FORM and record on patient's weekly data collection form and medical file. Report to Dr Anna Ralph and Dr Dina Bisara Lolong as per Safety Reporting Flwoshart.

Serious adverse event (SAE): any untoward medical occurrence in a clinical trial subject administered a pharmaceutical product, which is life-threatening, results in death, requires hospitalisation or prolongation of existing hospitalisation, results in significant disability / incapacity or congenital anomaly / birth defect. FILL IN SERIOUS ADVERSE EVENTS REPOTING FORM and record on patient's weekly data collection form and medical file. Report to Dr Anna Ralph and Dr Dina Bisara Lolong as per Safety Reporting Flwoshart.

Potential study drug-related AE

Arginine
- Diarrhoea
- Bloating
- Nausea
- Vomiting
- Mild to moderate abdominal pain
- Urticarial skin rash

Vitamin D
- Asymptomatic hypercalcaemia defined as iCa^{2+} > 1.32 mmol/L
- Symptomatic hypercalcaemia i.e. iCa^{2+} > 1.32 mmol/L with one or more of the following symptoms or signs:
  - Gastrointestinal: Nausea, Vomiting, Abdominal pain, Constipation, Anorexia
  - Neurological / Psychiatric: Fatigue, Irritability, Confusion, somnolence, Coma
  - Renal: Polyuria, dehydration
  - Others: Bone pain, itch.

Syndromic management of adverse events adapted from (1)

Side effect	Study drug most likely to be responsible	TB drug most likely to be responsible	Perform liver function tests	Perform serum ionised calcium	Management		
					Hypercalcaemia	Abnormal LFTs identified	No biochem. abnormality
**Minor**							
Mild to moderate anorexia, nausea, abdominal pain, bloating	Either L-arginine or vitamin D	Rifampicin > isoniazid	Yes	Yes	Withdraw patient from study, manage hypercalcaemia in standard fashion[1]	Follow standard protocol (monitor closely; consider H and R cessation if LFT elevation >5x normal)	Advise taking medications with meals
Mild to moderate diarrhoea	arginine	Rifampicin > isoniazid	Yes	No		As above	No change required unless diarrhoea is severe and persistent
Constipation	vitamin D	none	No	Yes	Withdraw patient from study, manage hypercalcaemia in standard fashion[1]		Standard treatment
Joint pains	neither	pyrazinamide	No	No	Give aspirin or NSAID		
Burning sensation in feet	neither	isoniazid	No	No	Give pyridoxine 50-75mg daily		
Flu-like symptoms	neither	rifampicin	No	No	Aspirin, continuation of rifampicin		
Orange / red urine	neither	rifampicin	No	No	reassurance		
**Major**							
Severe Anorexia, nausea, abdominal pain	vitamin D > arginine	Rifampicin > isoniazid	Yes	Yes	Withdraw patient from study, manage hypercalcaemia in standard fashion[1]	Cease R &/or H and follow usual procedure	Cease arginine and follow steps below[2]
Severe diarrhoea	arginine	Rifampicin > isoniazid	Yes	No		Follow standard protocol (monitor closely; consider H and R cessation if LFT elevation >5x normal)	
Polyuria, dehydration	Vitamin D	none	No	Yes	Withdraw patient from study, manage hypercalcaemia in standard fashion[1]		
Non urticarial skin rash	neither	Most TB drugs	No	No	Stop all TB drugs. When rash resolved, restart in following order: H, R, Z, E, S (if S being used i.e. MDRTB)		
Urticarial skin rash	neither	streptomycin	No	No	Stop all TB drugs. When rash resolved, restart in following order: H, R, Z, E, S (if S being used i.e. MDRTB)		
Jaundice	neither	Most TB drugs	Yes	No		Stop TB drugs, reintroduce as per standard protocol	
Irritability, confusion, drowsiness, coma	Vitamin D	Most TB drugs	Yes	Yes	Withdraw patient from study, manage hypercalcaemia in standard fashion[1]	Stop TB drugs, reintroduce as per standard protocol	
Visual impairment	neither	ethambutol	No	No	Stop ethambutol and do not reintroduce		
Shock, purpura	neither	rifampicin	Yes	No	Stop rifampicin		

[1]**Treatment of hypercalcaemia** this will not be a cause for automatic unblinding. Management will be will comprise oral hydration or intravenous hydration with normal saline, and / or oral prednisolone 15-60mg steroids, depending on severity, as per Adverse Events docuiments (2).

[2]**Management of mild to moderate gastrointestinal upset attributed to arginine:** this will not be a cause for unblinding. Study subject should be advised to take medications with food.

[3]**Management of severe gastrointestinal upset attributed to arginine:** this will not be a cause for unblinding. Study subject should be advised to take medications with food and halve the dose of the study drug for 1 week. If symptoms resolved, resume standard study drug dose and reassess. If intolerable symptoms continue at half dose, cease study drugs and reassess.

References

1. Harries A, Maher D, Graham S. TB/HIV A clinical manual. Second Edition.: Stop TB Department, WHO Geneva; 2004.
2. Symptomatic hypercalcaemia. Therapeutic guidelines: Antibiotic eTG complete 2007 [cited 2007 April 20]; Available from: <http://proxy9.use.hcn.com.au/>

Appendix 6: Adverse Events Management

Safety Reporting Flowchart

Clinical Investigator : Determine if an Event is Serious

Yes

Serious Event Reporting

Research Assistant in Timika:

- Determine causality
- Notify Dr Anna Ralph and Dr Dina Bisara Lolong **WITHIN 24 HOURS.** If Anna not available, notify Dr Paul Kelly or Nick Anstey.
- Record on SAE form

Dr Anna Ralph and Dr Dina Lolong:

- Ensure that event is recorded in safety database
- Determine reportability:

Is the event*:

- Serious?
- Unexpected?
- Related to study drug?

Yes

Is the event:
Fatal or life-threatening?

Yes

Dr Anna Ralph and Dr Dina Lolong should notify and supply the SAE form **WITHIN 24 HOURS** to the Data Safety Monitoring Committee by fax or email, who will then decide whether BPOM and the HRECs need to be notified **WITHIN 7 DAYS.**

- Other Investigators should be notified as soon as possible

No

NonSerious Event Reporting

Research Assistant in Timika:

- Record on Non-serious Adverse Event Form *and* on Weekly Data collection form
- Notify Dr Anna Ralph and Dr Dina Bisara Lolong in the regular fortnightly report.

Dr Anna Ralph and Dr Dina Lolong:

- Notify BPOM in routine report
- Record event in safety database

Contacts
- Dr Anna Ralph, fax + 61 2 6125 0740, email anna.ralph@anu.edu.au
- Dr Dina Bisara Lolong, fax 62 21 424 5386, email dina_lolong@litbang.depkes.go.id
- Dr Paul Kelly, fax + 61 2 6125 0740, email paul.kelly@anu.edu.au
- Dr Nick Anstey. Nicholas.anstey@menzies.edu.au

Data Safety Monitoring Committee:
- Dr Paulus Sugiarto MD, Chair. RSMM Hospital.
- Dr Louise Maple-Brown, Louise.Maple-Brown@menzies.edu.au

No (not reportable) / No (not fatal or life-threatening)

Dr Anna Ralph and Dr Dina Lolong should notify and supply the SAE form **WITHIN 24 HOURS** to the Data Safety Monitoring Committee, who will then decide whether BPOM and the HREC need to be notified **WITHIN 15 DAYS**

- Other Investigators should also be notified within 15 calendar days

*Classification of Serious Adverse Events

Serious

- Death
- Life-threatening condition
- Hospitalisation

Unexpected

The following are NOT unexpected, they are recognized potential adverse effects:

- Diarrhoea
- Bloating
- Nausea
- Vomiting
- Mild to moderate abdominal pain
- Asymptomatic hypercalcaemia defined as iCa^{2+} > 1.32 mmol/L
- Symptomatic hypercalcaemia i.e. iCa^{2+} > 1.32 mmol/L with one or more of the following symptoms or signs:
    - Gastrointestinal: Nausea, Vomiting, Abdominal pain, Constipation, Anorexia
    - Neurological / Psychiatric: Fatigue, Irritability, Confusion, somnolence, Coma
    - Renal: Polyuria, dehydration
    - Others: Bone pain, itch.

Related to study drug

Unrelated
Adverse event is clearly due to extraneous causes (e.g., underlying disease, environment)
Unlikely (must have 2)
Does not have temporal relationship to intervention.
1. Could readily have been produced by the subject's clinical state.
2. Could have been due to environmental or other interventions.
3. Does not follow known pattern of response to intervention.
4. Does not reappear or worsen with reintroduction of intervention.

Possible (must have 2)
1. Has a reasonable temporal relationship to intervention.
2. Could not readily have been produced by the subject's clinical state.
3. Could not readily have been due to environmental or other interventions.
4. Follows a known pattern of response to intervention.

Probable (must have 3)
1. Has a reasonable temporal relationship to intervention.
2. Could not readily have been produced by the subject's clinical state or have been due to environmental or other interventions.
3. Follows a known pattern of response to intervention.
4. Disappears or decreases with reduction in dose or cessation of intervention.

Definite (must have all 4)
1. Has a reasonable temporal relationship to intervention.
2. Could not readily have been produced by the subject's clinical state or have been due to environmental or other interventions.
3. Follows a known pattern of response to intervention.
4. Disappears or decreases with reduction in dose or cessation of intervention and recurs with re-exposure.

Hypercalcaemia in ADAPT patients

- Any patient found to have ionized calcium > 1.32 for the first time should have the test repeated.
- If iCa > 1.32 mmol/L at baseline, patient not eligible for enrolment in the study.
- If iCa > 1.32 mmol/L at any reading during treatment, follow the flow chart below.
- Hospitalization should be avoided where possible (IV saline can be given in the TB clinic or outpatient clinic); requirement for hospitalisiation in severe, symptomatic people will be discussed on a case-by-case basis.

**Hypercalcaemia Grade**	**Ionised calcium**	**Equivalent total calcium level (=iCax2)**
Mild	1.32 – 1.39 mmol/L	2.60 – 2.79 mmol/L
Moderate	1.40 – 1.49 mmol/L	2.80 – 2.99 mmol/L
Severe, potentially symptomatic	1.50 – 2.25 mmol/L	3.00 – 4.49 mmol/L
Very severe	> 2.25 mmol/L	> 4.50 mmol/L

Symptomatic hypercalacaemia will be defined as one or more of the following symptoms or signs occurring in a person with ionised calcium > 1.5 mmol/L

1.1.1.1 Gastrointestinal: Nausea, Vomiting, Abdominal pain, Constipation, Anorexia

1.1.1.2 Neurological / Psychiatric: Fatigue, Irritability, Confusion, somnolence, Coma

1.1.1.3 Renal: Polyuria, dehydration

1.1.1.4 Others: Bone pain, itch.

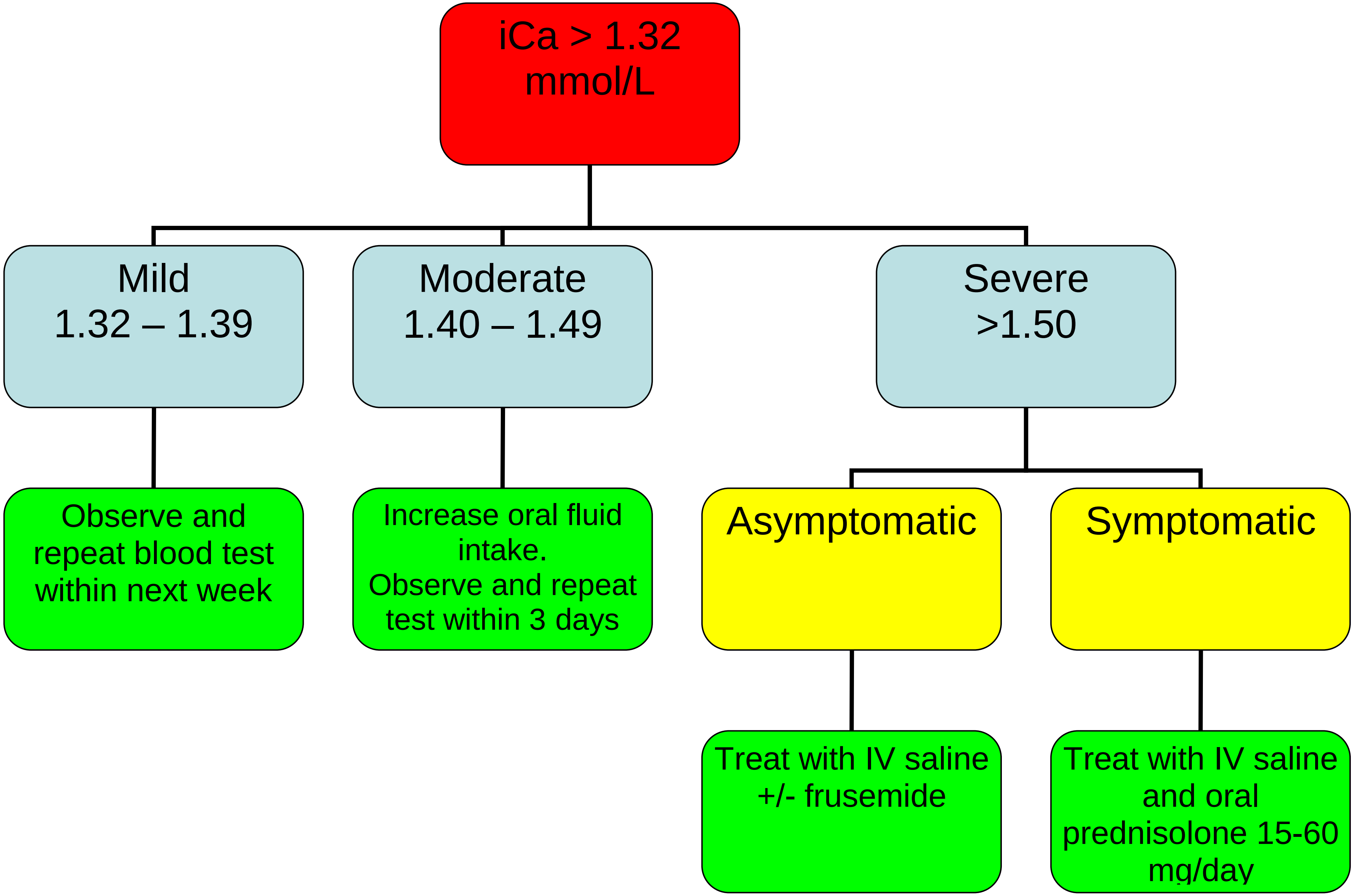
iCa > 1.32 mmol/L
Mild 1.32 – 1.39
Moderate 1.40 – 1.49
Severe >1.50
Observe and repeat blood test within next week
Increase oral fluid intake. Observe and repeat test within 3 days
Asymptomatic
Symptomatic
Treat with IV saline +/- frusemide
Treat with IV saline and oral prednisolone 15-60 mg/day

Adverse Events Reporting Form
(Non-Serious)

If any adverse experiences (including intercurrent illnesses) occurred between this visit and the previous visit, complete this page including your signature. If no adverse experiences occurred, check "None". Sign and date this page at lower right.

None ☐

**Description of Event Onset Date**	**Onset Date**	**End Date**	**Ongoing?**	**Duration**	**Type**	**Severity**	**Was the vent serious?**	**Relationship to Study Drug***	**Effect on Test Drug**	**Outcome of Event**	**Treatment Required**
If the event can be defined as a syndrome rather than a grouping of individual symptoms, please list as a syndrome.	dd/mm/yy	dd/mm/yy	0 = No<br>1 = Yes	Complete only if duration is less than 24 hours	1 = Single Occurrence<br>2 = Intermittent<br>3 = Continuous	1 = Mild<br>2 = Moderate<br>3 = Severe	0 = No<br>1 = Yes	0 = None (disease related)<br>1 = None (intercurrent illness)<br>2 = Unlikely (remote)<br>3 = Possible<br>4 = Probable<br>5 = Related	0 = None<br>1 = Reduced<br>2 = Interrupted<br>3 = Discontinued	0 = Recovered<br>1 = Improved with residual effects<br>2 = Remained the same<br>3 = Worsened<br>4 = Unknown	0 = None<br>1 = Medication<br>2 = Hospitalisation<br>3 = Other
	__|__|__	__|__|__									
	__|__|__	__|__|__									
	__|__|__	__|__|__									
	__|__|__	__|__|__									
	__|__|__	__|__|__									
	__|__|__	__|__|__									
	__|__|__	__|__|__									
	__|__|__	__|__|__									
	__|__|__	__|__|__									
	__|__|__	__|__|__									

1Complete the Serious Adverse Experience / Withdrawal Report Form and notify [Sponsor] immediately.
2Enter in the Concomitant Medications Form

______________________________ ___|___| 20___

Investigator's signature Date

Serious Adverse Events Reporting Form

Report Date:		Report Type: Initial ☐	Follow-up ☐
Study Drug(s):	UNKOWN: Blinded study	Investigator Name:	
Site:	RSMM ☐	TB Clinic ☐	Puskesmas ☐
Subject Information: Number:	TAD-|__|__|__|__|	Name: Date of Birth: |___|___| ________	Gender: M ☐ F ☐

Serious Adverse Event Description:

SAE Start Date:

Check applicable category(ies) for the SAE:

- ☐ Fatal
- ☐ Life-threatening
- ☐ Caused or prolonged in-patient hospitalization
- ☐ Significant or permanent disability
- ☐ Congenital anomaly/Birth defect
- ☐ Intervention to prevent any of the other
- ☐ Other - Specify:

Was the subject withdrawn from the study due to the SAE?	Yes ☐	No ☐		
Maximum Intensity:	Mild ☐	Moderate ☐	Severe ☐	
Outcome:	Resolved ☐	Ongoing ☐	Death ☐	Unknown ☐

Outcome Date: |___|___| 20____

Causal relationship to study drug (Check one)*:

☐ None	☐ Probable
☐ Unlikely (Remote)	☐ Highly Probable
☐ Possible	☐ Unknown

Study Drug Information:

1 Oral L-arginine / placebo Total Daily Dose:

Start Date: |___|___| 20______ Most Recent Stop Date: |___|___| 20_____ Treatment duration: weeks days

Drug Stopped Due to AE? Yes ☐ No ☐ N/A ☐

Dose Change Due to AE? None ☐ Reduced ☐ Suspended ☐

If Stopped or Suspended, was subject rechallenged? Yes ☐ No ☐ N/A ☐

If Yes, Date of Rechallenge: |___|___| 20_____

Did AE reappear after rechallenge? Yes ☐ No ☐ N/A ☐

2 Oral vitamin D / placebo Total Daily Dose:

Start Date: |___|___| 20______ Most Recent Stop Date: |___|___| 20_____ Treatment duration: weeks days

Drug Stopped Due to AE? Yes ☐ No ☐ N/A ☐

Dose Change Due to AE? None ☐ Reduced ☐ Suspended ☐

If Stopped or Suspended, was subject rechallenged? Yes ☐ No ☐ N/A ☐

If Yes, Date of Rechallenge: |___|___| 20_____

Did AE reappear after rechallenge? Yes ☐ No ☐ N/A ☐

Has Investigator unblinded treatment? Yes ☐ No ☐ N/A ☐

If Yes, Date of Unblinding: |___|___| 20_____

Subject Treatment arm: Active Drug ☐ Placebo ☐

Concomitant Medications:

Drug Name	Indication	Start date	Dose	Stop Date

Narrative:

Other Relevant History:

Investigator Signature Date

***Relatedness of Adverse Event to an Intervention**

Unrelated
Adverse event is clearly due to extraneous causes (e.g., underlying disease, environment)
Unlikely (must have 2)
Does not have temporal relationship to intervention.
1. Could readily have been produced by the subject's clinical state.
2. Could have been due to environmental or other interventions.
3. Does not follow known pattern of response to intervention.
4. Does not reappear or worsen with reintroduction of intervention.

Possible (must have 2)
1. Has a reasonable temporal relationship to intervention.
2. Could not readily have been produced by the subject's clinical state.
3. Could not readily have been due to environmental or other interventions.
4. Follows a known pattern of response to intervention.

Probable (must have 3)
1. Has a reasonable temporal relationship to intervention.
2. Could not readily have been produced by the subject's clinical state or have been due to environmental or other interventions.
3. Follows a known pattern of response to intervention.
4. Disappears or decreases with reduction in dose or cessation of intervention.

Definite (must have all 4)
1. Has a reasonable temporal relationship to intervention.
2. Could not readily have been produced by the subject's clinical state or have been due to environmental or other interventions.
3. Follows a known pattern of response to intervention.
4. Disappears or decreases with reduction in dose or cessation of intervention and recurs with re-exposure.

Source: http://www.niaid.nih.gov/ncn/sop/adverseevents.htm

Appendix 7: Healthy (Control) Subjects

Control subjects will be required in order to obtain normal Papuan and Non-Papuan population values for serum vitamin D, amino acids and T cell CD3 zeta expression, the 6 minute walk test and the Modified St George Respiratory Questionnaire.

Methods

- As per our previously approved malaria studies at the same study site, we will recruit 50 healthy adult Papuan and 50 healthy Non-Papuan people from among clinic and hospital staff or visitors. In each ethnic group, we will seek to enrol 25 men and 25 non-pregnant women who consent to participate.

Inclusion criteria

- Healthy adults >15 years of age
- Consent to having one-off blood test and 6 minute walk test
- Chronic stable conditions (such as hypertension) not impacting on ability to perform 6 minute walk

Exclusion criteria

- Pregnancy
- Hospitalisation currently or within the last month
- Current illness
    - Diagnosis by a doctor of any acute illness or active infection.
    - No fever or history of fever in the last week
    - Uncontrolled baseline hypertension: systolic blood pressure > 180mmHg, diastolic blood pressure > 100mmHg
    - Uncontrolled baseline tachycardia, pulse > 120 bpm
    - Unstable angina pectoris

Enrolment Procedure

- The Research assistant will seek informed consent from potential control subjects after providing verbal and written information (see Appendix) regarding the purpose of their participation and what is required of them. For participants who cannot read the information sheet and consent form in Bahasa Indonesia, the research assistants will carefully and fully explain the information using a translator if required.
- 20mL venous blood, a safe amount, will be collected in the manner described in Appendix 16 and transferred to lithium heparin tubes for separation and cryopreservation for plasma vitamin D, plasma amino acids (and metabolites), and white cell function and phenotype (including T cell CD3 zeta expression).
- Participants will be instructed on how to perform a 6 Minute Walk test (6MWT), and this will be undertaken as described in Appendix 13.

TAD- |__|__|__|__|

Appendix 8: Control Subject Information and Consent Sheet (English)

Participant Information Sheet: Normal Healthy People in Timika, Indonesia

Understanding how TB causes lung disease

<u>This is for you to keep</u>

We would like to ask you to take part in a study testing how far you can walk in 6 minutes and how TB causes lung damage. We have chosen you because you are healthy. This is because we want to compare your results with people with tuberculosis (TB), a lung illness which affects breathing. To find out how their lungs compare with normal, we need to test healthy people to see how far they can walk and how well their blood may fight infection.

If you want to take part, we will ask you a few questions about your health, check how much you weigh, then ask you to walk along the marked track for 6 minutes. We will also ask you for a small amount of blood: 20 ml (about 4 teaspoons): this will be used to examine the ability of healthy people to fight infection, so we can compare this to people with TB. These tests will not cost you anything.

All information collected is confidential. No personal identification will be revealed to persons outside the study.

<u>You do not have to participate if you don't want to, this is entirely voluntary.</u>

These tests will not benefit you directly. But by helping us better understand how TB causes disease, we may be better able to prevent and treat TB in the future.

If you have any questions about the study you can telephone Dr Paulus Sugiarto at RS Mitra Masyarakat Hospital on 901-301 881. <u>If you have any concerns or complaints</u> about the conduct of the study, please contact Dr Sandjaja, Chair of the NIHRD Ethics Committee in Jakarta on 081319304040 or 6202104261088, or The Secretary, Human Research Ethics Committee of the NT Department of Health & Community services & Menzies School of Health Research in Australia on 61-8-8922 7922

ADAPT Healthy Controls

Consent Form

Understanding how TB causes lung disease

This form means you can say No.

I have read the information sheet and have had the details explained to me by the witness below. I understand that I am not sick and will only need to answer questions, have my weight measured, a blood test (20 mls = 4 teaspoons), and walk for 6 minutes. I understand that all information collected is confidential and no information will be available to anyone outside the study. I understand that I do not have to participate in this study. I can withdraw from this study at any time for any reason.

PARTICIPANT

I (print name)

……………………………………………………………………… agree to take part in this study.

Signed Date

……………………………………………………………………… ______ / _______ / 20_____

PARTICIPANTS AGED <18 YEARS: PARENT / GUARDIAN TO SIGN

I (print name) agree for my child / person in my care to take part in this study.

………………………………………………………………………

Participant name

………………………………………………………………………

Signed Date

……………………………………………………………………… ______ / _______ / 20_____

WITNESS

I (print name) have explained the study & information sheet

………………………………………………………………………

Signed Date

……………………………………………………………………… ______ / _______ / 20_____

Appendix 8: Control Subject Information and Consent Sheet (Indonesian)

Surat Informasi Peserta:

Orang yang Sehat di Timika, Indonesia

Memahami bagaimana Tuberculosis (TB) menyebabkan penyakit paru-paru

Salinan ini bagi Anda

Kami ingin meminta Anda mengikuti studi yang meguji berapa jauh Anda bisa berjalan kaki dalam 6 menit dan bagaimana TB menyebabkan paru-paru menjadi sakit. Anda dipilih karena Anda sehat. Demikian, kami ingin membandingkan hasil Anda dengan hasil penderita TB, penyakit paru-paru yang memengaruhi pernafasan. Untuk mendapat bandingan antara paru-paru penderita TB dan orang lain, kami harus memeriksa orang-orang yang sehat untuk mengetahui berapa jauh bisa berjalan kaki dan bagaimana kemujaraban darah untuk melawan infeksi.

Jika Anda ingin mengikuti studi ini, Anda ditanyakan kesehatan Anda, berat badan diukur, lalu diminta berjalan kaki di jalan tertentu selama 6 menit. Kami juga meminta mengambil darah Anda sedikit saja, 20 ml (4 sendok teh), yang akan digunakan untuk memastikan kemampuan pelawanan infeksi orang sehat supaya dapat dibandingkan kemampuan penderita TB. Pemeriksaan tersebut tidak ada ongkos.

Semua informasi yang dikumpulkan tetap menjadi rahasia. Tidak ada informasi pribadi apapun yang dibuka kepada orang dari luar studi ini.

Anda tidak harus mengikuti studi ini, proses tersebut dengan sukarela sama sekali. Jika Anda mengikutinya, Anda boleh menarik diri kapan saja, dengan sebab apapun.

Pemeriksaan tersebut tidak akan bermanfaat langsung bagi Anda. Tetapi, dengan membantu kami lebih mengetahui bagaimana penyakit disebabkan TB, diharapkan pada masa depan TB dapat dicegah dan diobati secara lebih efektif.

Kalau ada pertanyaan mengenai studi ini silahkan Anda menelepon Dr Paulus Sugiarto di RS Mitra Masyarakat dengan nomor 901 301 881. Kalau ada pengabuan tentang pelaksanaan studi, silahkan menghubungi Dr Sandjaja, Ketua Komite Etika NIHRD di Jakarta dengan nomor telepon 081319304040 or 6202104261088, atau Sekretaris, *Human Research Ethics Committee of the NT Department of Health & Community Services and Menzies School of Health Research* di Australia dengan nomor 62 8 8922 7922.

Formulir Persetujuan Orang Sehat

Memahami bagaimana Tuberculosis (TB) menyebabkan penyakit paru-paru

Anda boleh mengatakan Tidak.

Saya sudah membaca Surat Informasi Peserta dan sudah menerima penjelasan perincian surat itu dari saksi yang namanya tertulis dibawah. Saya memahami saya tidak sakit dan hanya diminta menjawab beberapa pertanyaan, berat badan diukur, pemeriksaan darah (20 ml = 4 sendok teh) dilakukan, dan berjalan kaki selama 6 menit.

Saya memahami semua informasi yang dikumpulkan tetap menjadi rahasia dan tidak ada informasi pribadi apapun yang dibuka kepada orang dari luar studi ini. Saya memahami saya tidak harus mengikuti studi ini, dan boleh menarik diri kapan saja, dengan sebab apapun.

PESERTA

Saya (tuliskan nama) .... setuju dengan mengikuti studi ini.

Tanda tangan ....

Tanggal ______ / ______ / 20_____

PESERTA YANG BERUMUR <18 TAHUN: DITANDATANGANI ORANG TUA / WALI

Saya (tuliskan nama) .... menyetujui anak saya/orang dibawah asuhan saya mengikuti studi ini.

Nama peserta

....

Tanda tangan ....

Tanggal ______ / ______ / 20_____

SAKSI

Saya (tuliskan nama) .... sudah menjelaskan studi dan surat informasi.

Tanda tangan ....

Tanggal ______ / ______ / 20_____

Appendix 9: Control Subject Data Collection Form

FORMULIR PENGUMPULAN DATA ORANG SEHAT

Healthy people data collection form

Bagain A : Nama Peserta dan Pewawancara

Section A: Study Participant and data collector name

1. Tanggal hari: |__|__|/|__|__|/20__|__|

Date today

2. Nama [pertama]: ______________________________

First Name

3. Nama [keluarga]: ______________________________

Surname

4. Nama pengumpul data :

____

Data collector

Bagain B: Kriteria Inklusi dan Eksklusi

Section B: Inclusion and Exclusion Criteria

5. Umur > 15 th: Ya ☐1 Tidak ☐2

Age >15yrs

6. Resting pulse <120/min? Ya ☐1 Tidak ☐2

7. Resting BP<180/100? Ya ☐1 Tidak ☐2

8. Persetujuan Consent untuk berpartisipasi dalam penelitian ini: Ya ☐1 Tidak ☐2

Consent given

Bila menjawab “tidak” untuk pertanyaan no. 1-5, pasien tidak bisa dimasukkan dalam penelitian ini.

If ***‘No’*** *to any questions 1 to 5, PATIENT IS NOT ELIGIBLE*

9. Currently sick? (febrile illness ot diagnosed by doctor with illness during the last week)? Ya ☐1 Tidak ☐2

10.Unstable angina present? Ya ☐1 Tidak ☐2

Bila pasien menjawab "Ya" untuk pertanyaan no. 6-7, pasien tidak bisa diikutkan dalam penelitian ini.
*If **'Yes'** to question 6-7, PATIENT IS NOT ELIGIBLE*

Bagain C : Data Peserta

Section C: Study Participant data

11. Alamat: ____
Address

12. Telepon: |__|__|__|__|__|__|__|__|__|__|
Phone

13. Tanggal kelahiran: |__|__| / |__|__| / 19|__|__|
Date of birth: dd/mm/yyyy

14. Umur: **____tahun____bulan**
Age yr mo

15. Jenis Kelamin: Laki ☐1 Perempuan ☐2
Sex: M1,F2

16. Suku: Papuan ☐1 Bukan Papuan ☐2
Ethnicity

16a. Bila Papuan:
Amungme ☐1
Dani ☐2
Moni ☐3
Damal ☐4
Ekari ☐5
Kamoro ☐6
Nduga ☐7
Other ☐8

16b. Daerah asal:
Origin: h'land1 / l'land2
Daerah pegunungan ☐1
Daerah dataran ☐2

17. Riwayat merokok? Smoker?
Merokok ☐1
Pernah merokok tetapi sudah berhenti ☐2
Tidak pernah merokok ☐3

17a. Jika merokok, cigs/day?
Berapa banyak rokok yang diisap per hari ? ________ / hari

Bagain D :

Section D: Clinical and laboratory measurements

18. Jam berapa makan terakhir ? ______________ **Jam yang lalu**

How long ago did patient last eat? (hrs)

19. Pulse ______________ **ppb**

20. BP ______________ **mmHg**

21. Bllod collected Ya ☐1 Tidak ☐2

22. eNO ______________ **ppb**

23. Berat badan ______________ **kg**

weight

24. Height ______________ **m**

height

25. Tes jalan 6 menit ______________ **m**

6-min walk test

26. Kuisioner Respiratori St George Ya ☐1 Tidak ☐2

SGRQ done yes / no

Appendix 10: Extrapulmonary TB Survey and Data Collection Sheet

Only patients who have smear positive pulmonary TB (with or without additional extrapulmonary sites of infection) will be eligible for ADAPT. In order to gain an understanding of the context of these cases, intercurrent instances of extrapulmonary TB (EPTB) in children and adults in RSMM and Timika Puskesmus will be monitored. Patients with both smear positive pulmonary and extrapulmonary disease could be enrolled in both studies.

- **Study design:**
  - Descriptive study comprising a prospective audit

- **Background:**

EPTB is recognised internationally to be a difficult diagnostic entity. Preliminary work demonstrates differences in rates of extra-pulmonary TB in Papuans and Non-Papuans in Timika. However the details of EPTB type, diagnostic method, treatment and outcome have not been available for Timika to date. This proposed preliminary descriptive study of EPTB aims to answer these questions, and furthermore, to promote the use of existing protocols for diagnosing EPTB (e.g. Pedoman Nasional Penanggulangan TB Sistem Skoring for Children). Education in EPTB diagnosis and management will be provided to medical staff, and heightened efforts to retain patients in treatment for the full 6 months will be made, with treating doctors being requested to follow up patients and complete data collection forms at 2 and 6 months.

The potential change in HIV prevalence which might occur during the study period is also likely to have an impact on presentation of TB. Specific aspects of the presentation and management of TB-HIV co-infected patients will be evaluated, and education and support in HIV management will be provided as part of standard clinical care at RSMM and the TB clinic. This topic is well placed to be developed further by a future student into research which could form the basis of post-graduate studies.

- **Aims:**
  - To evaluate the burden and nature of extra-pulmonary TB in Papuan and Non-Papuan Timika residents

- **Methods:**
  - All adults and children presenting to RSMM or Timika TB Clinic with suspected EPTB will be eligible for enrolment in the study.
  - The treating doctor will be asked to follow standard NTP practice in diagnosing and treating EPTB, which includes offering HIV testing. The treating doctor will be asked to complete a form at baseline and again at 2 and 6 months to indicate demographic, clinical, laboratory and outcome characteristics (PLEASE SEE THE FOLLOWING 3 DATA COLLECTION FORMS: Baseline, 2-months, 6-months)
  - Given the planned study design (chart review), it will not be necessary for direct interaction between the study team and the patient except where this is in the course of standard clinical interaction.
  - If suitable biological specimens are collected during routine clinical care, portions of these will be saved for subsequent Rapid Diagnostic Protoeme™ antigen testing.

- **Timeline:**
  - No biological specimens additional to standard care are required for this study, therefore in anticipation of lack of signed consent requirement and rapid ethical clearance, this study could commence in early 2008.

Appendix 11: HIV-TB Co-infection

HIV rates are anticipated to be rising in Timika. To evaluate the burden of HIV-TB co-infection and uptake of antiretroviral treatment, the NTP policy of offering HIV testing to all people with TB will be supported. This is routinely offered as part of standard clinical practice in Timika. **Dr Dina Bisara Lolong** will be involved in the evaluation of HIV-TB co-infection.

- **Background**

Rising international rates of HIV-TB co-infection have created new challenges in the diagnosis and management of TB. Important questions remain unanswered, particularly in the Papuan context. Research priorities in TB-HIV co-infection identified by a WHO / Stop TB / UN working group(86) include:

1. Preventive therapy for TB (population versus individual levels); e.g: INH preventive therapy (primary and secondary prophylaxis), intensified case-finding to reduce community burden of contagious TB
2. Defining ways to implement UNAIDS/WHO policy of opt-out HIV testing
3. Co-trimoxazole: efficacy and optimal timing of commencement for opportunistic infection prophylaxis in different settings, delivery strategies.
4. Validating optimal time for commencement of antiretroviral therapy in TB-HIV co-infection
5. Development / evaluation of new diagnostic algorithms and other tools (e.g. rapid diagnostic tests) to improve diagnosis of smear negative TB in adults and children

To begin to address these issues in the Papuan context, a descriptive epidemiological study of current HIV-TB co-infection rates and ARV uptake and adherence is required. A comparative study of current HIV-TB rates versus 2003-4 figures (from previous work by members of the study team) can be undertaken.

This topic is well placed to be developed by a future student further to form the basis of potential post-graduate studies, for instance incorporating the ideas listed above.

- **Aims:**
  - To determine factors associated with HIV-TB – related morbidity
  - To evaluate the burden of HIV-TB co-infection, in particular, extra-pulmonary versus pulmonary manifestations of TB in HIV co-infected patients.
  - To evaluate uptake of and adherence to antiretroviral treatment and opportunistic infection prophylactic treatment in Papuan and Non-Papuan Timika residents with pulmonary and extra-pulmonary TB.

- **Methods:**
  - Patients enrolled in either ADAPT or the EPTB chart review will be eligible for inclusion
  - HIV testing is routine for all TB patients, and will performed on site in Timika using two tests (ELISA using Determine^{TM}, Abbott Diagnostics, Jakarta, Indonesia and a confirmatory ELISA or Western Blot)
  - Outcomes of TB treatment and contributing factors will be compared in patients with and without HIV
  - Antiretroviral treatment commencement, adherence and side effects will be recorded, with advice to treating doctors to adhere to WHO protocols relating to TB-HIV co-infection
  - Results will be compared with data previously collected by NIHRD-MSHR Researchers in Timika (2003-4)

Extrapulmonary TB Data Collection Form
BASELINE

STUDY: **ETB** ******WRITE ETB STUDY & CODE NUMBER IN PATIENT'S MEDICAL FILE***
CODE: ___________ (Range: 1-500)
SITE: Hospital Inpatient / Hospital Outpatient / Clinic
Data Collector: ____________
Date: ___ / ___ / 20___
Name: Family __________________ Given__________________
Address: ____
Telephone: ________________

DOB ___ / ___ / ______ Age: ____ yrs **Gender:** M / F
Place of origin: Highland / Lowland
Ethnicity: Papuan / Non Papuan If Papuan, state Suku ______________
Smoker: Current / Past / Never

Baseline Clinical Data

Weight___________kg
BCG scar present: Yes / No
Illness duration______________months
Prior treatments used? Yes / No
If yes, describe treatments received: Traditional / Western / Other (please specify)
____
History of past TB treatment? Yes / No
If yes, what year? ___________
Outcome if known: Cure / Completed / Defaulted
Known TB contact? Yes / No
If yes, Please specify: Household member / other__________________
Confirmed smear positive contact? Yes / No
HIV test: Positive / Negative
If positive: Diagnosis date: _____/_____/_____ On treatment? Yes / No
If on treatment, which medications? D4T / 3TC / Nevirapine / AZT / Efavirenz / Other___________

Extrapulmonary TB Diagnosis

TB Diagnosis date ______/_____/_____
How was extrapulmonary TB diagnosed?
Laboratory diagnosis:
Microscopy (BTA): Yes / No
Fluid analysis (e.g. lymphocyte-predominant exudate): Yes / No
Histopathology: Yes / No
Clinical diagnosis
ADULTS: Strong clinical evidence and decision made by doctor to treat: Yes / No
CHILDREN: Score >=6 using Pedoman Nasional Penanggulangan TB Sistem Skoring? Yes / No
For all children, complete table below:

Pedoman Nasional Penanggulangan TB Sistem Skoring for Children

Parameter	0	1	2	3
**TB contact**	Unknown		Reported by family	Confirmed AFB positive
**Tuberculin test**	Negative			Positive >=10mm (5mm if immuno-suppressed)
**Weight / nutritional status**		Weight loss <60%	Weight loss >60%	
**Unexplained fever**		>=2 weeks		
**Cough**		>=3 weeks		

**Lymph node enlargement**		>= 1 cm, more than 1 node affected, non painful		
**Bone or joint swelling**		Swelling present		
**Chest x ray**	Normal or unknown		Suggestive of TB	
**TOTAL**				

A. Clinical Diagnostic Information

TB Symptoms & signs: circle any present

Dry cough / Cough with clear phlegm / purulent phlegm / bloody phlegm /
Fever / Malaise / Weight loss / Anorexia / Dyspnoea / Jaundice /
Nausea / Vomiting / Diarrhoea / Headache / Reduced consciousness /
Abdominal swelling / Joint swelling (specify which)______________________
Bone pain or deformity (specify where)____________________________________
Other (please specify)____

On the basis of symptoms plus signs and any test results, indicate which sites affected by TB:

Lung / Pleura / Peritoneum / Pericardium / Lymph Node /
Bone / Peripheral Joint / Vertebral column / Brain /
Liver / Spleen / Kidney / Disseminated /
Gynaecological Other________________________________

B. Laboratory Diagnostic Information

Indicate in table which sites if any were positive by microscopy, histopathology, radiological findings or clinical evidence (tick boxes as required):

	Fluid cell count	Fluid bio-chemistry	BTA+	Histopath	Xray or ultrasound
**CSF**					
**Sputum** (for people with pulmonary and extrapulmonary TB)					
**Pleural Fluid**					
**Peritoneal Fluid**					
**Urine**					
**Joint Fluid**					
**Lymph Node**					
**Other__________**					

Extrapulmonary TB Data Collection Form
2-MONTH FOLLOW-UP

STUDY: **ETB**
CODE: ___________ (Range: 1-500)
Data Collector: ____________
Date: ___ / ___ / 20___
Name: Family ___________________ Given___________________

A. Diagnosis

Response to TB treatment? Yes / No
Extrapulmonary TB diagnosis: Definite / Likely / Not likely / Alternative diagnosis
If extrapulmonary TB definite or likely, please proceed to Section B.
If alternative diagnosis, answer questions i - iii only (Sections B-C not required):
i) Specify new diagnosis _______________________
ii) Indicate if diagnosis confirmed or suspected: Confirmed / Suspected
iii) How long did the person receive TB treatment for?__________weeks

B. Progress

Weight ___________kg
HIV test (if different from baseline): Positive / Negative
If positive: **Date of diagnosis______/______/______**
On treatment? Yes / No
Which medications? D4T / 3TC / Nevirapine / AZT / Efavirenz / Other___________

Treatment

Medication	Adverse reaction
**Rifampicin**	**Yes / No**
**Isoniazid**	**Yes / No**
**Ethambutol**	**Yes / No**
**Pyrazinamide**	**Yes / No**
**Streptomycin**	**Yes / No**
**Steroids**	**Yes / No**
**Other____________**	**Yes / No**

C. Residual Disability

After 2 months, does the patient have any disability related to TB? Yes / No
If yes, please tick appropriate column:

	Mild	Moderate	Severe
Cognitive / Psychiatric			
Hearing			
Vision			
Bone / Joint Deformity			
Problem Using Limb			
Pain: specify where__________________			
Breathlessness			
Cough			
Scarring			
Discharge (e.g. from scrofuloderma)			
Other___________________			
Other___________________			

Extrapulmonary TB Data Collection Form
6-MONTH FOLLOW-UP

STUDY: **ETB**
CODE: ___________ (Range: 1-500)
Data Collector: ____________
Date: ___ / ___ / 20___
Name: Family ___________________ Given___________________

A. Diagnosis

Response to TB treatment? Yes / No
Extrapulmonary TB diagnosis: Definite / Likely / Not likely / Alternative diagnosis
If extrapulmonary TB definite or likely, please proceed to Section B.
If alternative diagnosis, answer questions i - iii only (Sections B-D not required):
i) Specify new diagnosis ________________________
ii) Indicate if diagnosis confirmed or suspected: Confirmed / Suspected
iii) How long did the person receive TB treatment for?__________weeks

B. Progress
Weight ___________kg
HIV test (if different from baseline): Positive / Negative
If positive: **Date of diagnosis______/______/______**
On treatment? Yes / No
Which medications? D4T / 3TC / Nevirapine / AZT / Efavirenz / Other___________

Treatment

Medication	Duration (months)	Adverse reaction
**Rifampicin**		**Yes / No**
**Isoniazid**		**Yes / No**
**Ethambutol**		**Yes / No**
**Pyrazinamide**		**Yes / No**
**Streptomycin**		**Yes / No**
**Other_________________**		**Yes / No**

Duration of hospitalisation (0 if never hospitalised):_______days
TB sensitivity results: Available / not available
if available: Fully sensitive / Multi-drug resistant / Extremely drug resistant

C. Outcome
- **Treatment Completed:** Yes / No
- **Cured:** Yes / No
- **Treatment failure:** Yes / No
- **Defaulted:** Yes / No If yes, indicate date_____/_____/20____
- **Died:** Yes / No If yes, indicate date_____/_____/20____
- **Transferred out:** Yes / No If yes, indicate date_____/_____/20____
  If yes, indicate destination_____________________
  Outcome if known ____________________________

D. Residual Disability
After 6 months, does the patient have any disability related to TB? Yes / No
If yes, please tick appropriate column:

	**Mild**	**Moderate**	**Severe**
Cognitive / Psychiatric			
Hearing			
Vision			
Bone / Joint Deformity			
Problem Using Limb			
Pain: specify where__________________			
Breathlessness			
Cough			

Scarring			
Discharge e.g. from scrofuloderma)			
Other___________________			
Other___________________			

Appendix 11: Lung Function Testing

Spirometry will be performed as previously in Timika(87) using the ML3535C, MicroLoop (Micro Medical, Kent, UK), with single-use one-way valve mouthpieces according to American Thoracic Society (ATS) criteria. The equipment will be calibrated daily. Local staff have previously been trained in use of the device. Measurements will be performed in the purpose-built open-air clinic annex. The filters used with spirometry will prevent transmission of TB.

Each patient will perform at least three maximum effort expirations until volumes vary less than 200mL, with the highest values for FVC and FEV_1 used as measures of lung function. Dr Graeme Maguire will supervise the QA/QC of this test.

Appendix 12: Six minute walk test Methodology

The 6 minute walk test (6MWT) is test is a measure of submaximal functional exercise capacity which correlates with ability to perform activities of daily living. It determines the distance an individual can walk on a flat surface in a period of 6 minutes. The six minute weight.walk distance (6MWWD), calculated by multiplying this distance in kilometres by the subject's weight in kilograms, has been shown to give a more accurate correlation with assessments of lung function such as DLCO, FEV1 and peak oxygen uptake, and therefore will be the measure used in this study.

Method

6MWT will be performed in accordance with ATS guidelines.

- Weight will be measured without shoes to the nearest 0.1kg using an adult balance.
- The marked corridor at the back of the TB Puskesmas or in front of the ICU ward at RSMM will be the site for the 6 minute walk test.
- The researcher will keep time, count the number of laps, measure the stopping point, record the distance walked, and calculate the 6 minute weight.walk distance.
- Equipment available will include:
  - Chair in case the participant needs to rest
  - Oxygen
  - Sphygmomanometer
  - Telephone
- After participating in the 6MWT, the participant will be offered a refreshment and on opportunity to rest.

Appendix 13: Exhaled nitric Oxide measurement

Exhaled NO (eNO) will be used to measure pulmonary NO production at the TB Puskesmas and RSMM, using the online NIOX Mino® (Aerocrine, Sweden) device. The Mino is well validated, portable, easy to use, has inbuilt infection control and does not require calibration. Measurements will comply with ATS 2005 Guidelines(88), will be made prior to spirometry and 6 minute walk test, and subjects will be requested to refrain from smoking or eating for at least one hour before testing. Measurements will be performed in the purpose-built open-air clinic annex. The filters used with eNO will prevent transmission of TB. Consultant Cheryl Salome will supervise the QA/QC of eNO measurements.

Testing individual subjects

Exhaled NO will be measured at Week 0, 2, 4, 8 and 24 for each study participant. In a sub-set of up to 60 patients, eNO evaluation in Weeks 0 and 8 will be repeated up to 3 times over a 24 hour period to accompany blood collection for arginine pharmacokinetic /pharmacodynamic information. For each eNO measurement, the subject will be educated on use of the NIOX Mino® and mime what is required. The subject will then be instructed as follows:

1. Empty lungs
2. Inhale deeply through the disposable filter
3. Exhale through the filter at the required rate, indicated by a symbol of a smiling cloud on the NIOX Mino® screen.

The result will then be viewed on the NIOX Mino® display. Since correct technique including required exhalation rate are reported by the device, one test only will be obtained on each testing occasion if correct technique has been achieved.

Testing the device

The NIOX Mino® does not require calibration. Periodic measurements using a biological control and NO gas standard will be used to ensure comparability between sensor packs and exclude drift. This will be performed once per month or every 50 tests, whichever comes first, and at the start of each new sensor pack (every 300 tests). A subject whose eNO result is established will provide a measurement, and the manufacturer-supplied NO gas standard will be passed through the device via controlled compression of an air-tight bag at the same flow rate as is required for expired air. If drift is documented, appropriate correction of results using a conversion factor will be applied. Dr Cheryl Salome will supervise the QA/QC of eNO measurements.

Appendix 14: Sputum Specimen Handling and Processing

Table 6: Summary of Sputum testing to be performed for ADAPT

TEST	VOLUME REQUIRED	LOCATION OF TESTING	METHOD
**Sputum**			
Microscopy	1 mL	RSMM &/or TB Clinic, Timika<br>QA at WHO Reference Laboratory, Adelaide	Ziehl Nielsen stain
Proteome Systems' rapid diagnostic (RD) test	3 mL	RSMM &/or TB Clinic, Timika,	Antigen detection kit
QA Culture, Drug Sensitivity, Molecular typing	8 mL	WHO Supranational Reference laboratory, Adelaide	Mycobacterium Growth Indicator Tube (MGIT), Standard methods (see below). MIRU typing.

Sputum Collection and Handling

After collection in sterile specimen containers, sputum specimens will be kept in a domestic refrigerator at +4°C in the TB clinic or RSMM until ready for batched transport by air with a commercial courier via Darwin to the WHO Supranational Reference laboratory at the Institute of Medical and Veterinary Sciences (IMVS) in Adelaide, Australia. Preparation and packaging of the specimens will comply with International Air Transport Association (IATA) regulations. Specimens will not be decontaminated prior to storage, and will be sent at three to six week intervals during the study period. After leaving the TB clinic or RSMM Laboratory, specimens will be kept at room temperature until arrival at the reference laboratory. We have previously demonstrated success in culturing MTB in over 94% of smear positive sputum samples, despite considerable delays in transport to the Adelaide laboratory (mean 18.7, range 4-42 days).(89)

Location of testing

Wherever possible, specimens will be processed locally. Thus sputum microscopy and the novel antigen testing will be performed in Timika. There is no capacity for TB culture in Timika. It is our understanding that the nationally accredited TB culture laboratories in Indonesia (in Surabaya and Jakarta) are operating at maximum capacity and are fully committed to work related to the ongoing National TB drug resistance survey. Furthermore, it is mandatory for TB research to demonstrate participation in a quality assurance program. We therefore propose that sputum culture, sensitivity and typing should be performed at the WHO Supranational Reference Centre (IMVS, Adelaide) with whom the National TB Control Program is currently collaborating with a range of training, guideline development and quality assurance activities. Our previous experience of transferring samples from Timika to the WHO reference Laboratory has been successful. Additionally, processing at the WHO reference Laboratory will provide excellent training opportunities for NIHRD laboratory scientist (Ibu Palupi) to work on Indonesian samples in a supportive environment with world class training.

Methods

- ***Ziehl Nielsen (ZN) staining and light microscopy***

ZN staining is performed routinely at Timika Puskasmas and RSMM laboratories in accordance with NTP guidelines.

- ***Proteome Systems' rapid diagnostic (RD) test***

3 mL sputum will be reserved for antigen testing. Protease will be added and the sample will be frozen for subsequent batched antigen testing using Proteome Systems' rapid diagnostic (RD) test^{TM} at RSMM laboratory.

- ***Culture***

Upon arrival at IMVS, Sputum specimens will be decontaminated using 2% sodium hydroxide and 0.5% N-acetyl-cysteine for 25 min, then neutralised to pH 7, concentrated by centrifugation (3,000xg for 15 min) and inoculated into a single Mycobacterium Growth Indicator Tube 960 (MGIT; Becton Dickinson Microbiology Systems, Sparks, Md). Microscopy of sputum concentrates will be performed using fluorochrome and ZN stains and results recorded using standard IUATLD grading scales. Contaminated specimens will be re-decontaminated with 4% sulphuric acid.

Isolates of *M. tuberculosis* will be identified by ZN staining, hybridisation with commercial nucleic acid probes (Accuprobe, Gen-Probe, Inc., San Diego, Calif.), and biochemical investigations (eg. nitrate reduction)(90, 91). The IMVS successfully participates in external quality assessment programmes through the WHO Global Supranational Reference Laboratory Quality Control Network and the Centers for Disease Control and Prevention, Atlanta, USA.

- ***Susceptibility***

Susceptibility tests will be performed using the BACTEC MGIT 960 TB system using the recommended critical concentrations for the following antibiotics: INH (0.1 and 0.4 mg/ml), RIF (1.0 mg/ml), EMB (5.0 mg/ml), SM (1.0 mg/ml), amikacin (AMK: 1.0 mg/ml), capreomycin (CAP: 2.5 mg/ml), ofloxacin (OFL: 2.0 mg/ml), and ethionamide (ETH: 5.0 mg/ml). The Wayne method of detecting pyrazinamidase activity will be used to infer pyrazinamide susceptibility(90).

- ***Micro-satellite Interstitial Repetitive Unit typing (MIRU)***

MIRU is a DNA typing method using polymerase chain reaction (PCR) to detect differences in the number of repetitive units in specific regions of the MTB genome (known as MIRU loci), using genomic DNA from cultured MTB isolates. We will target 12 MIRU's in order to genotype MTB isolates is this study. PCR amplification of MIRU loci will be performed in a volume of 25 mL containing: 2.5 mL GeneAmp 10x PCR Buffer II (Perkin-Elmer Cetus), 2 mM MgCl2, 100nM each primter, 200 mM each of 4 dNTPs and 0.625 U AmpliTaq Gold DNA Polymerase (Perkin-Elmer Cetus). An initial denaturation will be followed by 35 cycles of detauration / annealing / extension. Negative and positive controls will be included with each PCR round. The presence and size of each PCR product will be determined by electrophoresis on an agarose gel followed by staining with ethidium bromide.

Instructions for specimen collection, storage & transport (SPUTUM)

A. On day specimen is collected:

1. Collect fresh sputum specimen from patient at enrolment (after 6 minute walk test)
2. Label specimen (including TB ID number)
3. Divide sputum specimen using sterile technique in biosafety cabinet for
   - ZN testing in Timika lab
   - Specimen for Antigen test (Proteome RD test): ADD PROTEASE prior to storage
   - Specimen for IMVS
4. Ensure lid tightly closed
5. Tape down lid
6. Place sputum pot in biohazard bag and pack with cotton wool
7. Complete request form for IMVS (1 per sample). Place request form in outside sheath of biohazard bag
8. Place in refrigerator in TB clinic/RSMM Laboratory

B. In week of specimen transport

9. Prepare the Saf-T-PAK
   - Attach labels
   - Fill in addresses (shipper and consignee)
   - Fill in other information
10. Fill in Commercial invoice form
11. Fill in dangerous goods declaration. Note that you need 4 originals per shipment (must have red colour on sides).
12. Contact shipping agent on Tuesday before Thursday flight, specimens to shipping agent by Wednesday midday.
13. With Shipping Agent, complete checklist for dangerous goods. Seal Saf-T-PAK and attach all paper work to top. DO NOT cover any labels with tape
14. Contact study PIs and receiving laboratory by email to inform that shipment is coming.

Appendix 15: Blood and Urine Handling and Processing

Table 7: Summary of Blood and Urine testing to be performed for ADAPT

Included in the table are all tests which will be performed for the research which are *additional* to routine standard of care testing already occurring (e.g. HIV serology).

TEST	VOLUME REQUIRED	LOCATION OF TESTING	METHOD
**Blood**			
Ionised calcium (*i*Ca^{2+}), haemoglobin and white cell differential	1 mL	RSMM &/or TB Clinic, Timika	portable i-STAT cartridges, and microscopy
Proteome Systems' rapid diagnostic (RD) test	3 mL	RSMM, Timika	Proteome Systems' rapid diagnostic (RD) test™
Amino acids including arginine, and their metabolites	4 mL	MSHR, Darwin with NIHRD Scientist	HPLC
PBMC type, function & phenotype (includingCD3 zeta expression)	10 mL	MSHR, Darwin with NIHRD Scientist	FACS, ELISpot and proliferation assays
Plasma 1,25(OH)$_2$D$_3$ and parathormone	1.5 ml	MSHR, Darwin (clinical laboratory)	RIA
Plasma 25(OH)D$_3$	0.5 mL	RMIT University, Melbourne	LC-MS assay
	Total for testing = 20mL. This is a safe volume.		
**Urine**			
Proteome Systems' rapid diagnostic (RD) test	4 mL	RSMM, Timika	Proteome Systems' rapid diagnostic (RD) test™

Twenty mL venous blood (a safe amount) will be collected from participants at enrolment and at follow up weeks 2, 4, 8 and 24. In a sub-set of up to 60 patients, the single 20mL collection will be split over 3 collections (1x 12 mL collection and 2x 4 mL) within a 24 hour period for arginine pharmacokinetic / pharmacodynamic information. Blood collection will be performed by a member of the research team with adequate training and experience in venesection in a careful manner using strict sterile technique to minimise discomfort or bruising for the study subject, or risk of biohazard injury to staff. Blood will be taken using a disposable 20mL syringe. The sample will be transferred to lithium heparin tubes. The blood samples will be labelled with the patient's name, Study ID number, date of collection and the specimen number (tubes 1 to 2). All waste will be disposed of in a way which will minimise the chance of contamination.

1 Collection and Handling

Immediately following enrolment in the study, participants will have blood collected by a health professional with adequate training and experience in venesection, in a careful manner using strict sterile technique to minimise discomfort or bruising for the study subject, or risk of biohazard injury to staff. Blood will be used for (1) specific tests required by the study, and (2) tests required by the local treating doctor as per standard TB practice (including HIV testing, and any additional tests deemed necessary by the treating doctor, which may include liver function or full blood count). Blood required for the study will comprise: samples for immediate local processing (ionised calcium and haemoglobin using iSTAT) and for storage and later transport for off-shore processing (plasma arginine, vitamin D, T cell studies).

A mid-stream urine sample will also be collected for storage for subsequent antigen testing using Proteome Systems' rapid diagnostic (RD) test™ at RSMM laboratory.

2 Location of testing

In all instances, tests which are readily able to be performed locally will be processed in Timika. Where assays are unable to be performed in the local laboratory, samples will require storage for subsequent batched transport to MSHR and thence in some instances, to commercial laboratories for standard testing. Tests to be performed in Timika include: Calcium, Haemoglobin, HIV serology, and Proteome Systems' rapid diagnostic (RD) test on blood, urine and sputum. Tests which will be performed in Australia include vitamin D and parathyroid hormone, amino acids (arginine, citrulline, ornithine), and white cell studies. Standard operating protocols are as detailed below.

3 Methods

3.1 Blood tests in Timika

- ***HIV antibody (as part of routine clinical protocols)***

Following appropriate counselling as per normal clinical procedures in Timika, same-day testing for HIV using a rapid test (Determine™, Abbott, Jakarta) and a confirmatory ELISA at the RSMM Laboratory, and any other tests deemed to be necessary by the treating doctor (not for the study), e.g. liver function, full blood count.

- ***Calcium, Haemoglobin***

Portable i-STAT cartridges already in routine use at the hospital or clinic will be used for immediate processing for ionised calcium (iCa^{2+}) and haemoglobin.

- ***Proteome Systems' rapid diagnostic (RD) test (see also Point 3.3)***

Plsama will be reserved for antigen testing using Proteome Systems' rapid diagnostic (RD) test™ at RSMM laboratory. The urine specimen will also be stored for this purpose. Protease will be added to the specimens prior to storage.

3.2 Blood tests in Australia (SEE ALSO APPENDIX B)

Heparinised blood samples will be separated within 3 hours of collection into plasma and peripheral blood mononuclear cells (PBMCs) [using Ficoll Hypaque as is routine in the Timika lab]), stored at -70 (plasma) or in liquid nitrogen (PBMCs in foetal calf serum/DMSO) in our Timika research laboratory, then shipped by dry shipper at -70°C to MSHR within one month of collection.

- ***Vitamin D assay***

25(OH)D_3 and 1,25(OH)$_2D_3$ levels will be measured using DiaSorin LIASION® 25OH Vitamin D assay at a NATA-accredited commercial laboratory. Plasma 1,25(OH)$_2D_3$ and parathormone will also be measured in order to evaluate the possibility of increases in systemic vitamin D metabolites that may relate to efficacy and any adverse effects.

- ***Arginine assay***

Plasma amino acids (including metabolites) will be measured by HPLC.

- ***White cell studies***

PBMC phenotype and CD3 zeta expression will be determined by FACS, measuring the amount of bound intracellular anti-CD3ζ PE fluorescent antibody (Immunotech, France), as is routine in our lab. The shift in expression of CD3ζ between activated and naïve CD4 and CD8 T cells will be compared among patients in different study arms. Phenotypic analyses will be run in conjunction with functional assays to measure cell proliferation and cytokine secretion. Cell proliferation will be detected by using carboxyfluorescein diacetate succinimidyl ester (CFSE). Interferon-γ production in response to stimulus with mitogens, bacterial LPS and PPD will be determined using ex-vivo ELISpot assays. All assays are routinely run in the MSHR lab.

3.3 Proteome Systems' rapid diagnostic (RD) test

The Proteome Systems' rapid diagnostic (RD) test™ is in late stages of development and should be available for field testing by 2008. As noted above, this test will be performed on sputum and blood. Further samples which have been routinely collected as part of standard clinical care (e.g. ascitic fluid in extrapulmonary TB) which may be available for evaluation with the Proteome Systems' rapid diagnostic (RD) test™, can also be tested. In the event of field evaluation of this diagnostic test commencing, it will be performed by laboratory scientist **Ms. Kristina Retnoningtyas Palupi** at the RSMM laboratory.

Instructions for collection, storage & transport of samples: BLOOD

A. on day of collection

1. Use a disposable 20mL syringe and 18 gauge wing needle
2. Collect 20 mL (baseline) or 15 mL (follow up) venous blood
3. Inject 100 uL whole blood into iSTAT cartridge (avoiding bubbles), for calcium and haemoglobin
4. Transfer remainder of sample to two 10ml lithium tubes.
5. Label the tubes with the patient's name, Study ID number, date of collection and the specimen number (tubes 1 to 4).
6. Dispose of the needle in the sharps disposal unit and any other blood-contaminated equipment in biohazard bags.
7. Wait for iSTAT results before giving the patient their medications
8. If hypercalcaemia is identified (iCa^{2+}>1.32mmol/L), remove the vitamin D / placebo from the packet containing the study medications.
9. Put aside the heparin tubes for transfer to the RSMM lab within 3 hours of collection. Do not refrigerate.
10. Lab staff will then process bloods according to the protocol for separation and freezing.

B. On day of transport (where applicable)

11. Separated samples will be shipped by dry shipper at -70°C to MSHR within one month of collection.

Appendix 16: St George Respiratory Questionnaire (English Version)

Name ______________________ DOB __ / __ / ____ TB study ID number______

PART 1

(Circle correct answers)

1) Over the last year, I have coughed:
Most days
Several
A few
Only ______ days
Not

2) Over the last year, I have brought up phlegm (sputum):

Most days
Several
A few
Only ______ days
Not

3) Over the last year, I have had shortness of breath:

Most days
Several
A few
Only ______ days
Not

4) Over the last year, I have had attacks of wheezing:
Most days
Several
A few
Only ______ days
Not

5) During the last year, how many severe or very bad unpleasant attacks of chest trouble have you had?
More than three
3 attacks
2 attacks
1 attack
None

6) How long did the worst attack of chest trouble last?
a week or more
3 or more days
1 or 2 days
less than a day

7) Over the last year, in an average week, how many good days (with little chest trouble) have you had?
None
1 or 2
3 or 4
nearly every day
every day

8) If you have a wheeze, is it worse in the morning?
No
Yes

PART 2

9) How would you describe your chest condition?

The most important problem I have

Causes me quite a lot of problems

Causes me a few problems

Causes no problem

10) If you have ever had paid employment?

My chest trouble made me stop work

My chest trouble interferes with my work or made me change my work

My chest trouble does not affect my work

11) Questions about what activities usually make you feel breathless.

Sitting or lying still

Getting washed or dressed

Walking around the home

Walking outside on the level

Walking up a flight of stairs

Walking up hills

Playing sports or games

12) More questions about your cough and breathlessness.

My cough hurts

My cough makes me tired

I get breathless when I talk

I get breathless when I bend over

My cough or breathing disturbs my sleep

I get exhausted easily

13) Questions about other effects your chest trouble may have on you.

My cough or breathing is embarrassing in public

My chest trouble is a nuisance to my family, friends or neighbours

I get afraid or panic when I cannot get my breath

I feel that I am not in control of my chest problem

I do not expect my chest to get any better

I have become frail or an invalid because of my chest

Exercise is not safe for me

Everything seems too much of an effort

14) Questions about your medication.

My medication does not help me very much

I get embarrassed using my medication in public

I have unpleasant side effects from my medication

My medication interferes with my life a lot

15) Questions about how activities may be affected by your breathing.

I take a long time to get washed or dressed

I cannot take a bath or shower, or I take a long time

I walk more slowly than other people, or I stop for rests

Jobs such as housework take a long time, or I have to stop for rests

If I walk up one flight of stairs, I have to go slowly or stop

If I hurry or walk fast, I have to stop or slow down

My breathing makes it difficult to do things such as walk up hills, carry things up stairs, light gardening such as weeding, dance, play bowls or play golf

My breathing makes it difficult to do things such as carry heavy loads, dig the garden or shovel snow, jog or walk at 5 miles per hour, play tennis or swim

My breathing makes it difficult to do things such as very heavy manual work, run, cycle, swim fast or play competitive sports

16) We would like to know how your chest trouble usually affects your daily life.

I cannot play sports or games

I cannot go out for entertainment or recreation

I cannot go out of the house to do the shopping

I cannot do housework

I cannot move far from my bed or chair

17) Tick the statement which you think best describes how your chest affects you.

It does not stop me doing anything I would like to do

0 It stops me doing one or two things I would like to do

It stops me doing most of the things I would like to do

It stops me doing everything I would like to do

Note: A modified version of this questionnaire, translated into Bahasa Indonesia, has previously been used successfully at the study site. There are no added questions, but some of the possible responses have been modified to reflect local conditions and cultural practices. For example, references to shoveling snow in this tropical setting have been omitted.

Appendix 17: Cost-effectiveness analysis

Cost Effectiveness Analysis of L-Arginine or Vitamin D supplementation to improve Pulmonary TB treatment

The study will examine the cost-effectiveness of L-arginine or vitamin D supplementation in the clinical trial setting, compared with the cost effectiveness of unsupplemented TB treatment. Comparison of the cost effectiveness between the intervention and the control groups will permit policy decisions to be made regarding funding of these potential adjuvant TB treatments, if they are shown to be beneficial.

Hypotheses :

HO : Supplementation with L-arginine or Vitamin D are not cost-effective interventions to improve pulmonary TB cure rates.

H1 : Supplementation with L-arginine or Vitamin D are cost-effective interventions to improve pulmonary TB cure rates.

Method

Data collected directly from ADAPT study participants, and additional sources described below, will be used to conduct a cost-effectiveness analysis. The Cost Effectiveness analysis is used to compare the cost and effectiveness of the intervention group (with L-arginine or vitamin D supplementation) with cost and effectiveness for standard pulmonary TB treatment (without supplementation).

Cost Measurement :

Cost information will be collected from NTP reports, government and other official documents and from study participants following previously validated methods. This information will be used to count the following costs.

1. Total Health System Costs: The annual expenditure for NTP (including capital and recurrent costs), to calculate the cost per patient cured. A calculation will also be made about the costs of poor compliance in terms of relapse rates for TB (and hence more cases of TB to be treated) and the potential for the development of drug resistance (and hence more expensive treatments required).
2. Total Patient Costs: the total cost born by the patient from diagnosis, treatment and follow-up during the treatment. This includes transport costs, examinations fees, loss of income and other out of pocket expenditures.
3. Total Opportunity Costs: The total cost forgone by community and Community Health Workers caring for TB patients.
4. Costs of the intervention: The costs associated with the intervention (L-arginine or vitamin D) will be calculated by including all capital, recurrent and salary costs associated with this study.

The cost will be calculated in US dollars conforming to the currency currently used in Indonesia. The costs are estimated according to the local market price. Annual value of capital items are estimated based on expected useful life of 10 years for furniture and 5 years for vehicle and equipment. The discount rate is assumed accordingly. The office buildings can be estimated to be

10% of the overhead costs.

Effectiveness :

The effectiveness will be measured through the following indicators :

- Cure rate,
- Time of sputum conversion rate
- Clinical improvement

Cost-Effectiveness Analysis (CEA)

Cost–effectiveness will calculated by dividing the total cost per activity by each different effectiveness measure using the formula:

$$\text{Cost Per Patient Cure} = \frac{\text{Total Health System Costs + Total Patient Cost + Total opportunity Costs}}{\text{Total Number of Patient Cure}}$$

The cost per patient cure between intervention and control group will be compared and will make an inference about the cost-effectiveness of L-arginine or vitamin D intervention.

Appendix 18: Investigator Team

See Investigator List, page ii, for Investigator / Consultant status, qualifications and affiliations.

Our team of investigators is fully familiar with the content areas and techniques required to successfully answer our research questions. **Dr Sandjaja** is an expert in the field of nutrition in clinical medicine and therefore well placed to lead the study as Indonesian PI. **Prof Nick Anstey** has a long track record in NO and arginine research, with NIH/Welcome Trust-NHMRC funding for his work on NO/arginine and malaria, including randomised clinical trials of arginine supplementation at the Timika site. **A/Prof Paul Kelly** has been working with global TB control issues and TB research, including randomised controlled trials, for 20 years and in Indonesia for 10 years.

Drg. Franciscus Thio is a Papuan dental graduate with many years experience in health administration at the district level and, more recently, as a research administrator and leader at our field site in Timika. He will be studying for his PhD as part of the capacity building aspects of this project. **Dr Dina Bisara Lolong** is a medical graduate with a masters in demography who has previously worked in Papua at Puskesmas, District and provincial administrative levels. In recent years she has been a principle investigator on a range of TB and HIV related research projects.

Dr Enny Kenangalem, Papuan medical doctor and experienced clinician, or her replacement, will provide research leadership at our field site in Timika. **Ms. Kristina Retnoningtyas Palupi** is a Laboratory Scientist who will continue to develop her research skills as part of the capacity building aspects of this project. **Dr Lamria** and Ibu **Luxi** are NIHRD researchers who will participate in the research. **Dr Emiliana Tjitra** Senior Research Clinician will provide a supervisory and advisory role in the study. **Dr Andri Wiguna** and **Dr Enny Malonda** (or her replacement) are the clinicians working at the hospital and the clinic where the patients will be enrolled and followed up. **Bapak Govert Waramori** is a Papuan nurse who has worked with the research team for the past 4 years and has developed many technical skills. He has a deep understanding of the equipment and procedures in the field site and will be working towards his MPH as part of the capacity-building aspects of this project. **Dr Anna Ralph** is an experienced infectious diseases physician and NHMRC postgraduate scholar who will be based in Timika and undertake these studies for her PhD.

Dr Ric Price has extensive research experience with the NIHRD-MSHR collaboration and will provide database and analytical support. **Dr Graeme Maguire** has completed studies of lung function in Timika (TB) and other remote settings, including RCTs to improve lung function. He will ensure training and QA in lung function testing. **Dr Peter Morris** has coordinated many successful RCTs, in northern Australia, Indonesia and East Timor in conjunction with Nick Anstey and Paul Kelly. **Dr Ivan Bastian** and **Mr Richard Lumb** have worked with microbiology partners in Indonesia and his laboratory is the WHO Supranational TB reference laboratory. **Dr Tonia Woodberry** is a MSHR laboratory scientist who provides expertise in PBMC work in partnership with NIHRD laboratory partners. **Dr Indri Rooslamiati, Prof Stephen Duffull** and **Dr Tsin Yeo** will provide expertise and advice for pharmacokinetic / pharmacodynamic studies as required. **Dr Pasi Penttinen** and **Dr Firdy Permana** are important local public health collaborators. **Prof John Eisman** is a world authority on Vitamin D replacement and monitoring; **Dr Cheryl Salome** on QA/QC of exhaled NO measurements; **Prof Niels Becker,** is an expert statistician in infectious diseases modelling; **Dr Carmelia Basri** is the head of the Indonesian National TB Control Program and will ensure that study results are transformed to policy where appropriate.

14 APPENDICES B: Materials Transfer

Rationale for location of specimen processing

The new focus by NIHRD on TB culture and molecular typing in 2008 will require external quality control and possible requirements for training in-country or abroad. Specimens collected for the ADAPT study will be ideally suited for use in a QA program for the developing NIHRD-University of Indonesia collaborative TB Laboratory. Options for the provision of training by ADAPT study investigators in sputum culture and typing in Jakarta or Australia are under investigation.

In all instances, tests which are readily able to be performed locally will be processed in Timika. Where assays are unable to be performed in the local laboratory, samples will require storage for subsequent batched transport to MSHR and thence in some instances, to commercial laboratories for standard testing. Tests to be performed in Timika include: sputum smear microscopy, Calcium, Haemoglobin, HIV serology, and Proteome^{TM} Systems' rapid diagnostic (RD) test on blood, urine and sputum (when available). Tests which will be performed in Australia include sputum culture, sensitivity testing and genotyping, vitamin D and parathyroid hormone, amino acids (arginine, citrulline, ornithine), and white cell studies. Standard operating protocols are as detailed below.

Sputum Culture

There is no capacity for TB culture in Papua. We therefore propose doing sputum culture at the WHO reference laboratory, Adelaide, Australia, for the following reasons:

1. The laboratory in Adelaide is recognised by WHO as one of 7 global Supranational reference laboratories for TB testing. It provides this service for Australia, New Zealand, the Pacific and South-East Asia. It is within the mandate and capacity of this laboratory to provide a service to Indonesia. The WHO Supranational Reference Centre (IMVS, Adelaide) continues to collaborate with the Indonesian National TB Control Program with a range of training, guideline development and quality assurance activities.
2. It is our understanding that the nationally accredited TB culture laboratories in Indonesia (in Surabaya and Jakarta) are operating at maximum capacity and are fully committed to work related to the ongoing National TB drug resistance survey.
3. While these Indonesian laboratories therefore cannot take on the added burden of processing all sputum specimens from the ADAPT study, a regular random sample of divided specimens can be dispatched to the developing NIHRD-University of Indonesia collaborative TB Laboratory, when it becomes operational, for QA purposes.
4. To maintain the strong reputation of NIHRD-MSHR Research collaboration in international research and permit ongoing publication of outcomes in leading international journals, ethical and scientific integrity principles mandating participation in a quality assurance program need to be upheld. This is standard international practice in the conduct of high-quality TB research.
5. Our previous experience of transferring samples from Timika to the WHO reference Laboratory has been successful.
6. Processing of samples at the WHO reference Laboratory will provide excellent training opportunities for NIHRD laboratory scientist (Ibu Palupi) to work on Indonesian samples in a supportive environment with world class training.

Vitamin D

1. A particular problem recognised among vitamin D assays is that of variance in results, even at the most reputable laboratories, due to unavoidable fluctuations in reagents between batches and over time. A variety of vitamin D testing methods exist, and this further increases the problem of inter-laboratory variance.(92, 93) It is recognised amongst vitamin D researchers that the variability accepted in clinical practice is too excessive according the stringent requirements of research where accurate determination within 10 nmol/L is critical. Use of standard (non-reference) laboratories for vitamin D research requires QA testing on a high proportion if not all samples, significantly elevating costs; even then, results can be difficult to interpret due to inter-test variability.

2. The newer **liquid chromatography tandem mass spectrometry (LC-MS) method for vitamin D determination** is increasingly recognised as the gold standard for vitamin D measurement. Laboratories using this method have demonstrated superior consistency of results compared with RIA or chemiluminescent assays. A laboratory offering this test has now become available in Australia (Division of Laboratory Medicine, Royal Melbourne Institute of Technology University, Victoria), and is in the process of publishing results showing superior results (lower coefficient of variance) compared with other standard vitamin D assay methodologies. This information will be forwarded to the NIHRD Ethics Committee as soon as it is available. Thus it would be in the interests of best quality research to utilise this laboratory for the measurement of vitamin D levels. The volume of blood required for LC-MS vitamin D testing is lower than that required by standard methods (100-300 microL).

3. Development of the LC-MS method is described by Maunsell Z et al.(94) In their discussion, they state:

   *"Recent publications have highlighted the interlaboratory variability of 25-OH D analysis on patient samples measured by RIA and chemiluminescence assays (24) and quality assurance material (22). In view of this, some authors have suggested that there should be international standardization of assays and have suggested that until that is achieved, RIA techniques should be used for clinical analyses (24). **However, the use of a routine LC-MS/MS method offers a real alternative and negates the use of radioactive tracers**."*

4. The LC-MS method has been chosen as the vitamin D assay method for the UK trial of vitamin D in tuberculosis which is currently recruiting patients. The same researchers have already published results of studies of vitamin D in latent TB using the same method (see Martineau AR et al, 2007). **For our study to be optimally comparable to this research, the same vitamin D assay methodology should be used.**

DRAFT MATERIALS TRANSFER AGREEMENT: MSHR

(Final versions in PDF format included in Regulatory Binder)

The National Institute of Health Research and Development (NIHRD), hereby referred to as the **First Party**, agrees to provide (**name, institution),** hereby referred to as the **Second Party**, with the biological specimens and / or data (Material) described in Appendix A for use in the study and for specific assays (Research Plan) described in Appendix B, under the direct oversight and responsibility of (**Scientist**). Note that both Appendix A and Appendix B are hereby considered as integral parts of this document and that the terms described herein apply to both the Institution and Scientist irrespective of their association with each other. The First Party and The Second Party agree to the following conditions:

1. The Material shall be used exclusively by the Second Party and be limited to the Scientist, and others as expressly stated in Appendix B, for executing the Research Plan, and for no other purpose, unless this agreement is amended in writing by expressed written consent of both parties.

2. The Second Party will assure that the Material is not distributed, released, or disclosed by any means, either intentional or accidental, to any person or entity other than those listed in the Research Plan. It is understood that all persons listed in the Research Plan are under the direct responsibility of the Second Party as well as the Scientist and the actions of such persons, and consequences thereof, with respect to the Material and Research Plan are thereby considered the full responsibility of the Second Party and the Scientist.

3. In accordance with the signed *Agreement on Research Collaboration Between The Menzies School of Health Research and NIHRD* (dated 12 June 2003), intellectual property rights arising from this Agreement shall be jointly owned by the First and Second Party. Both parties shall be allowed to use such property for non-commercial purposes free of royalty. Both parties are entitled to the royalties on the principle of equitable contribution if they are used for commercial purposes.

4. In accordance with the signed *Agreement on Research Collaboration Between The Menzies School of Health Research and NIHRD* (dated 12 June 2003), both parties have the same rights and access to specimens and data collected from approved studies. Upon a reasonable request made by the First Party, taking into consideration the logistics of transporting samples and whether time has elapsed since the completion of the Research Plan, the Second Party will return any and all unused Material to the first Party within thirty (30) days of written request. Costs of specimen transport will be negotiated between the First and Second Parties.

5. The second Party will transfer, in confidence, to the First Party any and all raw data, records, and results derived from the Material and Research Plan, including detailed records of direct use of Material. In addition, such information will be provided, where possible, within thirty (30) days of written request by the First Party.

6. In accordance with both parties' institutional agreements with the Wellcome Trust (signed by both NIHRD and MSHR in 2003), both parties will promote the publication of data arising from research funded by The Wellcome Trust, where possible in open-access journals. The use of any data, results, or concepts, hereby termed outputs, derived from use of the Material in presentations, abstracts, publications (both peer-reviewed and not peer-reviewed), grants, or other means of dissemination by the Second Party or Scientists will be determined by mutual agreement between the Parties, in accordance with the signed

Agreement on Research Collaboration Between The Menzies School of Health Research and NIHRD (dated 12 June 2003). Authorship of publications arising from the Research Plan shall be by mutual agreement, and shall comply with international guidelines outlined in the Vancouver Agreement.

7. It is fully acknowledged by the Second Party that the Material is experimental in nature and it is provided without warranty of fitness for the purposes described in the Research Plan. Moreover, the First Party makes no claims or warranty that the use of the material will not infringe on any unforeseen patents or proprietary rights and accepts no responsibility for such infringements. In no event shall the First Party be held liable in any way for any use, expenses, loss, claim, damage or liability of any kind which may arise from or in connection with this Agreement or the use, handling or storage of the Material.

8. The Second Party agrees to use the Material in compliance with all laws, governmental regulations and guidelines applicable to the Material, including any specially applicable to research with human DNA and with potentially hazardous materials.

This Agreement shall be governed by and interpreted in accordance with the laws of the Government of Indonesia.

_____________________________ _____________________________

First Party Representative Date
Dr Endang R. Sedyaningsih Mamahit, MPH, Dr.PH
Head, Biomedical and Pharmaceutical Division
Biomedical and Pharmaceutical Branch
The National Institute of Health Research and Development

_____________________________ _____________________________

Second Party Institutional Representative Date
Name: Mr Brendon Douglas
Title: Executive Officer, MSHR

_____________________________ _____________________________

Second Party Scientist: Date
Name: Professor Nicholas Anstey
Title: Head, International Health Division, MSHR

REFERENCES

1. Dondorp A, Nosten F, Stepniewska K, Day N, White N. Artesunate versus quinine for treatment of severe falciparum malaria: a randomised trial. Lancet. 2005 Aug 27-Sep 2;366(9487):717-25.
2. Ratcliff A, Siswantoro H, Kenangalem E, Maristela R, Wuwung RM, Laihad F, et al. Two fixed-dose artemisinin combinations for drug-resistant falciparum and vivax malaria in Papua, Indonesia: an open-label randomised comparison. Lancet. 2007 Mar 3;369(9563):757-65.
3. Ratcliff A, Siswantoro H, Kenangalem E, Wuwung M, Brockman A, Edstein MD, et al. Therapeutic response of multidrug-resistant Plasmodium falciparum and P. vivax to chloroquine and sulfadoxine-pyrimethamine in southern Papua, Indonesia. Trans R Soc Trop Med Hyg. 2007 Apr;101(4):351-9.
4. Hagusian RA, Purba LH, Kanangalem E, Wuwung M, Maristela R, Rumaseuw R, et al. Dihydroartemisinin-piperaquine versus artesunate-amodiaquine for multi-drug resistant P.flaciparum and P.vivax in Papua, Indonesia. Clin Infect Dis 2007;in press.
5. Dye C. Global epidemiology of tuberculosis. Lancet. 2006 Mar 18;367(9514):938-40.
6. WHO. Global tuberculosis control – surveillance, planning, financing WHO/HTM/TB/2006.362 ed: WHO, Geneva; 2006.
7. Gwatkin D. Health inequalities and the health of the poor: what do we know? What can we do? . Bull WHO 2000;78(1):3-18.
8. Russell S. The economic burden of illness for households in developing countries: a review of studies focusing on malaria, tuberculosis, and human immunodeficiency virus/acquired immunodeficiency syndrome. Am J Trop Med Hyg. 2004 Aug;71(2 Suppl):147-55.
9. Raviglione MC, Snider DE, Jr., Kochi A. Global epidemiology of tuberculosis. Morbidity and mortality of a worldwide epidemic. Jama. 1995 Jan 18;273(3):220-6.
10. Raviglione MC, Uplekar MW. WHO's new Stop TB Strategy. Lancet. 2006 Mar 18;367(9514):952-5.
11. Chaulk CP, Kazandjian VA. Directly observed therapy for treatment completion of pulmonary tuberculosis: Consensus Statement of the Public Health Tuberculosis Guidelines Panel. Jama. 1998 Mar 25;279(12):943-8.
12. Onyebujoh P, Rodriguez W, Mwaba P. Priorities in tuberculosis research. Lancet. 2006 Mar 18;367(9514):940-2.
13. Connolly L, Edelstein P, Ramakrishnan L. Why Is Long-Term Therapy Required to Cure Tuberculosis? PLoS Medicine. 2007;4(3):e120 (435-42).
14. North RJ, Jung YJ. Immunity to tuberculosis. Annu Rev Immunol. 2004;22:599-623.
15. Orme I. Adaptive immunity to mycobacteria. Curr Opin Microbiol. 2004 Feb;7(1):58-61.
16. Nathan C. Inducible nitric oxide synthase in the tuberculous human lung. Am J Respir Crit Care Med. 2002 Jul 15;166(2):130-1.
17. Chan J, Tanaka K, Carroll D, Flynn J, Bloom BR. Effects of nitric oxide synthase inhibitors on murine infection with Mycobacterium tuberculosis. Infect Immun. 1995 Feb;63(2):736-40.
18. MacMicking JD, North RJ, LaCourse R, Mudgett JS, Shah SK, Nathan CF. Identification of nitric oxide synthase as a protective locus against tuberculosis. Proc Natl Acad Sci U S A. 1997 May 13;94(10):5243-8.
19. Nicholson S, Bonecini-Almeida Mda G, Lapa e Silva JR, Nathan C, Xie QW, Mumford R, et al. Inducible nitric oxide synthase in pulmonary alveolar macrophages from patients with tuberculosis. J Exp Med. 1996 May 1;183(5):2293-302.
20. Anstey NM, Weinberg JB, Hassanali MY, Mwaikambo ED, Manyenga D, Misukonis MA, et al. Nitric oxide in Tanzanian children with malaria: inverse relationship between malaria severity and nitric oxide production/nitric oxide synthase type 2 expression. J Exp Med. 1996 Aug 1;184(2):557-67.

21. Choi HS, Rai PR, Chu HW, Cool C, Chan ED. Analysis of nitric oxide synthase and nitrotyrosine expression in human pulmonary tuberculosis. Am J Respir Crit Care Med. 2002 Jul 15;166(2):178-86.
22. Wang CH, Liu CY, Lin HC, Yu CT, Chung KF, Kuo HP. Increased exhaled nitric oxide in active pulmonary tuberculosis due to inducible NO synthase upregulation in alveolar macrophages. Eur Respir J. 1998 Apr;11(4):809-15.
23. Cooper AM, Adams LB, Dalton DK, Appelberg R, Ehlers S. IFN-gamma and NO in mycobacterial disease: new jobs for old hands. Trends Microbiol. 2002 May;10(5):221-6.
24. Cooper AM, Pearl JE, Brooks JV, Ehlers S, Orme IM. Expression of the nitric oxide synthase 2 gene is not essential for early control of Mycobacterium tuberculosis in the murine lung. Infect Immun. 2000 Dec;68(12):6879-82.
25. Florquin S, Amraoui Z, Dubois C, Decuyper J, Goldman M. The protective role of endogenously synthesized nitric oxide in staphylococcal enterotoxin B-induced shock in mice. J Exp Med. 1994 Sep 1;180(3):1153-8.
26. Schon T, Elias D, Moges F, Melese E, Tessema T, Stendahl O, et al. Arginine as an adjuvant to chemotherapy improves clinical outcome in active tuberculosis. Eur Respir J. 2003 Mar;21(3):483-8.
27. Boger RH, Bode-Boger SM. The clinical pharmacology of L-arginine. Annu Rev Pharmacol Toxicol. 2001;41:79-99.
28. Morris CR, Morris SM, Jr., Hagar W, Van Warmerdam J, Claster S, Kepka-Lenhart D, et al. Arginine therapy: a new treatment for pulmonary hypertension in sickle cell disease? Am J Respir Crit Care Med. 2003 Jul 1;168(1):63-9.
29. Zea AH, Culotta KS, Ali J, Mason C, Park HJ, Zabaleta J, et al. Decreased expression of CD3zeta and nuclear transcription factor kappa B in patients with pulmonary tuberculosis: potential mechanisms and reversibility with treatment. J Infect Dis. 2006 Nov 15;194(10):1385-93.
30. Seitzer U, Kayser K, Hohn H, Entzian P, Wacker HH, Ploetz S, et al. Reduced T-cell receptor CD3zeta-chain protein and sustained CD3epsilon expression at the site of mycobacterial infection. Immunology. 2001 Nov;104(3):269-77.
31. Rodriguez PC, Quiceno DG, Zabaleta J, Ortiz B, Zea AH, Piazuelo MB, et al. Arginase I production in the tumor microenvironment by mature myeloid cells inhibits T-cell receptor expression and antigen-specific T-cell responses. Cancer Res. 2004 Aug 15;64(16):5839-49.
32. Rodriguez PC, Zea AH, Culotta KS, Zabaleta J, Ochoa JB, Ochoa AC. Regulation of T cell receptor CD3zeta chain expression by L-arginine. J Biol Chem. 2002 Jun 14;277(24):21123-9.
33. Rodriguez PC, Zea AH, DeSalvo J, Culotta KS, Zabaleta J, Quiceno DG, et al. L-arginine consumption by macrophages modulates the expression of CD3 zeta chain in T lymphocytes. J Immunol. 2003 Aug 1;171(3):1232-9.
34. Taheri F, Ochoa JB, Faghiri Z, Culotta K, Park HJ, Lan MS, et al. L-Arginine regulates the expression of the T-cell receptor zeta chain (CD3zeta) in Jurkat cells. Clin Cancer Res. 2001 Mar;7(3 Suppl):958s-65s.
35. Grad R. Cod and the consumptive: a brief history of cod-liver oil in the treatment of pulmonary tuberculosis. Pharm Hist. 2004;46(3):106-20.
36. Davies PD. A possible link between vitamin D deficiency and impaired host defence to Mycobacterium tuberculosis. Tubercle. 1985 Dec;66(4):301-6.
37. Roelandts R. The history of phototherapy: something new under the sun? J Am Acad Dermatol. 2002 Jun;46(6):926-30.
38. Dowling G, Prosser-Thomas E. Treatment of lupus vulgaris with calciferol. Lancet 1946:919-22.
39. Norman AW, Mizwicki MT, Norman DP. Steroid-hormone rapid actions, membrane receptors and a conformational ensemble model. Nat Rev Drug Discov. 2004 Jan;3(1):27-41.
40. Rook GA, Steele J, Fraher L, Barker S, Karmali R, O'Riordan J, et al. Vitamin D3, gamma interferon, and control of proliferation of Mycobacterium tuberculosis by human monocytes. Immunology. 1986 Jan;57(1):159-63.

41. Sly LM, Lopez M, Nauseef WM, Reiner NE. 1alpha,25-Dihydroxyvitamin D3-induced monocyte antimycobacterial activity is regulated by phosphatidylinositol 3-kinase and mediated by the NADPH-dependent phagocyte oxidase. J Biol Chem. 2001 Sep 21;276(38):35482-93.
42. Crowle AJ, Ross EJ, May MH. Inhibition by 1,25(OH)2-vitamin D3 of the multiplication of virulent tubercle bacilli in cultured human macrophages. Infect Immun. 1987 Dec;55(12):2945-50.
43. Rockett KA, Brookes R, Udalova I, Vidal V, Hill AV, Kwiatkowski D. 1,25-Dihydroxyvitamin D3 induces nitric oxide synthase and suppresses growth of Mycobacterium tuberculosis in a human macrophage-like cell line. Infect Immun. 1998 Nov;66(11):5314-21.
44. Chang JM, Kuo MC, Kuo HT, Hwang SJ, Tsai JC, Chen HC, et al. 1-alpha,25-Dihydroxyvitamin D3 regulates inducible nitric oxide synthase messenger RNA expression and nitric oxide release in macrophage-like RAW 264.7 cells. J Lab Clin Med. 2004 Jan;143(1):14-22.
45. Malik ZA, Thompson CR, Hashimi S, Porter B, Iyer SS, Kusner DJ. Cutting edge: Mycobacterium tuberculosis blocks Ca2+ signaling and phagosome maturation in human macrophages via specific inhibition of sphingosine kinase. J Immunol. 2003 Mar 15;170(6):2811-5.
46. Hmama Z, Sendide K, Talal A, Garcia R, Dobos K, Reiner NE. Quantitative analysis of phagolysosome fusion in intact cells: inhibition by mycobacterial lipoarabinomannan and rescue by an 1alpha,25-dihydroxyvitamin D3-phosphoinositide 3-kinase pathway. J Cell Sci. 2004 Apr 15;117(Pt 10):2131-40.
47. Liu PT, Stenger S, Li H, Wenzel L, Tan BH, Krutzik SR, et al. Toll-like receptor triggering of a vitamin D-mediated human antimicrobial response. Science. 2006 Mar 24;311(5768):1770-3.
48. Cegielski JP, McMurray DN. The relationship between malnutrition and tuberculosis: evidence from studies in humans and experimental animals. Int J Tuberc Lung Dis. 2004 Mar;8(3):286-98.
49. Karyadi E, Schultink W, Nelwan RH, Gross R, Amin Z, Dolmans WM, et al. Poor micronutrient status of active pulmonary tuberculosis patients in Indonesia. J Nutr. 2000 Dec;130(12):2953-8.
50. van Lettow M, Fawzi WW, Semba RD. Triple trouble: the role of malnutrition in tuberculosis and human immunodeficiency virus co-infection. Nutr Rev. 2003 Mar;61(3):81-90.
51. Laurens H. The Physiologic Effects of Ultraviolet Irradiation. JAMA 1938;11(26).
52. Ustianowski A, Shaffer R, Collin S, Wilkinson RJ, Davidson RN. Prevalence and associations of vitamin D deficiency in foreign-born persons with tuberculosis in London. J Infect. 2005 Jun;50(5):432-7.
53. Tiong AC, Patel MS, Gardiner J, Ryan R, Linton KS, Walker KA, et al. Health issues in newly arrived African refugees attending general practice clinics in Melbourne. Med J Aust. 2006 Dec 4-18;185(11-12):602-6.
54. Bellamy R, Ruwende C, Corrah T, McAdam KP, Thursz M, Whittle HC, et al. Tuberculosis and chronic hepatitis B virus infection in Africans and variation in the vitamin D receptor gene. J Infect Dis. 1999 Mar;179(3):721-4.
55. Roy S, Frodsham A, Saha B, Hazra SK, Mascie-Taylor CG, Hill AV. Association of vitamin D receptor genotype with leprosy type. J Infect Dis. 1999 Jan;179(1):187-91.
56. Lewis SJ, Baker I, Davey Smith G. Meta-analysis of vitamin D receptor polymorphisms and pulmonary tuberculosis risk. Int J Tuberc Lung Dis. 2005 Oct;9(10):1174-7.
57. Martineau AR, Honecker FU, Wilkinson RJ, Griffiths CJ. Vitamin D in the treatment of pulmonary tuberculosis. J Steroid Biochem Mol Biol. 2007 Mar;103(3-5):793-8.
58. Armas LA, Hollis BW, Heaney RP. Vitamin D2 is much less effective than vitamin D3 in humans. J Clin Endocrinol Metab. 2004 Nov;89(11):5387-91.
59. Morcos MM, Gabr AA, Samuel S, Kamel M, el Baz M, el Beshry M, et al. Vitamin D administration to tuberculous children and its value. Boll Chim Farm. 1998 May;137(5):157-64.
60. Nursyam EW, Amin Z, Rumende CM. The effect of vitamin D as supplementary treatment in patients with moderately advanced pulmonary tuberculous lesion. Acta Med Indones. 2006 Jan-Mar;38(1):3-5.

61. Grange JM, Davies PD, Brown RC, Woodhead JS, Kardjito T. A study of vitamin D levels in Indonesian patients with untreated pulmonary tuberculosis. Tubercle. 1985 Sep;66(3):187-91.
62. Hollis BW, Wagner CL. Normal serum vitamin D levels. N Engl J Med. 2005 Feb 3;352(5):515-6; author reply -6.
63. Diamond TH, Eisman JA, and members of the Australian and New Zealand Bone and Mineral Society ESoA, and Osteoporosis Australia. . Vitamin D and adult bone health in Australia and New Zealand: a position statement. Med J Aust. 2005 Mar 21;182(6):281-5.
64. Nathan C. Role of iNOS in human host defense. Science. 2006 Jun 30;312(5782):1874-5.
65. Adams JS. Vitamin D as a defensin. J Musculoskelet Neuronal Interact. 2006 Oct-Dec;6(4):344-6.
66. Adams JS, Ren SY. Autoregulation of 1,25-dihydroxyvitamin D synthesis in macrophage mitochondria by nitric oxide. Endocrinology. 1996 Oct;137(10):4514-7.
67. Adams JS, Ren SY, Arbelle JE, Clemens TL, Shany S. A role for nitric oxide in the regulated expression of the 25-hydroxy-vitamin D-1-hydroxylation reaction in the chick myelomonocytic cell line HD-11. Endocrinology. 1994 Jan;134(1):499-502.
68. Martineau AR, Wilkinson KA, Newton SM, Floto RA, Norman AW, Skolimowska K, et al. IFN-{gamma}- and TNF-Independent Vitamin D-Inducible Human Suppression of Mycobacteria: The Role of Cathelicidin LL-37. J Immunol. 2007 Jun 1;178(11):7190-8.
69. Kelly PM, Ardian M, Waramori G, Anstey NM, Syahrial H, Tjitra E, et al. A community-based TB drug susceptibility study in Mimika District, Papua Province, Indonesia. Int J Tuberc Lung Dis. 2006 Feb;10(2):167-71.
70. Munns C, Zacharin MR, Rodda CP, Batch JA, Morley R, Cranswick NE, et al. Prevention and treatment of infant and childhood vitamin D deficiency in Australia and New Zealand: a consensus statement. Med J Aust. 2006 Sep 4;185(5):268-72.
71. Byth K, Gebski V. Factorial designs: a graphical aid for choosing study designs accounting for interaction. Clin Trials. 2004;1(3):315-25.
72. Telzak EE, Fazal BA, Pollard CL, Turett GS, Justman JE, Blum S. Factors influencing time to sputum conversion among patients with smear-positive pulmonary tuberculosis. Clin Infect Dis. 1997 Sep;25(3):666-70.
73. Guler M, Unsal E, Dursun B, Aydln O, Capan N. Factors influencing sputum smear and culture conversion time among patients with new case pulmonary tuberculosis. Int J Clin Pract. 2007 Feb;61(2):231-5.
74. Morris CR, Kuypers FA, Larkin S, Sweeters N, Simon J, Vichinsky EP, et al. Arginine therapy: a novel strategy to induce nitric oxide production in sickle cell disease. Br J Haematol. 2000 Nov;111(2):498-500.
75. Clarkson P, Adams MR, Powe AJ, Donald AE, McCredie R, Robinson J, et al. Oral L-arginine improves endothelium-dependent dilation in hypercholesterolemic young adults. J Clin Invest. 1996 Apr 15;97(8):1989-94.
76. Drug Facts and Comparisons. : St Louis, Mo: Lippincott; 1984.
77. Rizzato G. Clinical impact of bone and calcium metabolism changes in sarcoidosis. Thorax. 1998 May;53(5):425-9.
78. Abbasi AA, Chemplavil JK, Farah S, Muller BF, Arnstein AR. Hypercalcemia in active pulmonary tuberculosis. Ann Intern Med. 1979 Mar;90(3):324-8.
79. Fuss M, Karmali R, Pepersack T, Bergans A, Dierckx P, Prigogine T, et al. Are tuberculous patients at a great risk from hypercalcemia? Q J Med. 1988 Nov;69(259):869-78.
80. Liam CK, Lim KH, Srinivas P, Poi PJ. Hypercalcaemia in patients with newly diagnosed tuberculosis in Malaysia. Int J Tuberc Lung Dis. 1998 Oct;2(10):818-23.
81. Roussos A, Lagogianni I, Gonis A, Ilias I, Kazi D, Patsopoulos D, et al. Hypercalcaemia in Greek patients with tuberculosis before the initiation of anti-tuberculosis treatment. Respir Med. 2001 Mar;95(3):187-90.
82. Moher DS, KF. Altman, DG. for the CONSORT Group. Revised recommendations for improving the quality of reports of parallel group randomized trials 2001. 2001.

83. Editors. ICoMJ. International Committee of Medical Journal Editors. Uniform requirements for manuscripts submitted to biomedical journals. www.icmje.org (accessed June 2007) Updated May 2000. .
84. Harries A, Maher D, Graham S. TB/HIV A clinical manual. Second Edition.: Stop TB Department, WHO Geneva; 2004.
85. Symptomatic hypercalcaemia. Therapeutic guidelines: Antibiotic eTG complete 2007 [cited 2007 April 20]; Available from: <http://proxy9.use.hcn.com.au/>
86. WHO. TB/HIV Research Priorities in resource-limited settings: Report of an expert consultation. WHO, Geneva. 2005;WHO/HTM/TB/2005.355.
87. Handojo T, Anstey N, Kelly P, Pain M, Kenangalem E, Tjitra E, et al. Normal spirometry, gas transfer and lung volume values in Papua, Indonesia. Southeast Asian J Trop Med Public Health. 2006 May;37(3):571-7.
88. ATS/ERS recommendations for standardized procedures for the online and offline measurement of exhaled lower respiratory nitric oxide and nasal nitric oxide, 2005. Am J Respir Crit Care Med. 2005 Apr 15;171(8):912-30.
89. Lumb R, Ardian M, Waramori G, Syahrial H, Tjitra E, Maguire GP, et al. An alternative method for sputum storage and transport for mycobacterium tuberculosis drug resistance surveys. Int J Tuberc Lung Dis. 2006 Feb;10(2):172-7.
90. Kent PT, Kubica GPE. Public Health Mycobacteriology: a guide for the level III laboratory.: Atlanta, GA: US Department of Helath and Human Services; 1985.
91. Metchock B, Nolte FS, Wallace RJJ. Mycobacterium. In: Murray PR, Baron EJ, Pfaller MA, Tenover FC, Yolken RH, eds. Manual of clinical microbiology, 7th Ed: pp 399-437: Washington DC, ASM Press.; 1999.
92. Binkley N, Krueger D, Cowgill CS, Plum L, Lake E, Hansen KE, et al. Assay variation confounds the diagnosis of hypovitaminosis D: a call for standardization. J Clin Endocrinol Metab. 2004 Jul;89(7):3152-7.
93. Hollis BW. Editorial: The determination of circulating 25-hydroxyvitamin D: no easy task. J Clin Endocrinol Metab. 2004 Jul;89(7):3149-51.
94. Maunsell Z, Wright DJ, Rainbow SJ. Routine isotope-dilution liquid chromatography-tandem mass spectrometry assay for simultaneous measurement of the 25-hydroxy metabolites of vitamins D2 and D3. Clin Chem. 2005 Sep;51(9):1683-90.
