# Supplementary material for: L-arginine and Vitamin D Adjunctive Therapies in Pulmonary Tuberculosis: A Randomised, Double-Blind, Placebo-Controlled Trial
Source: PLoS One. 2013 Aug 14;8(8):e70032. doi: 10.1371/journal.pone.0070032 (PMC3743888; doi:10.1371/journal.pone.0070032)
Supplement: Checklist S1 — Supporting CONSORT checklist. (DOC) [file pone.0070032.s002.doc]

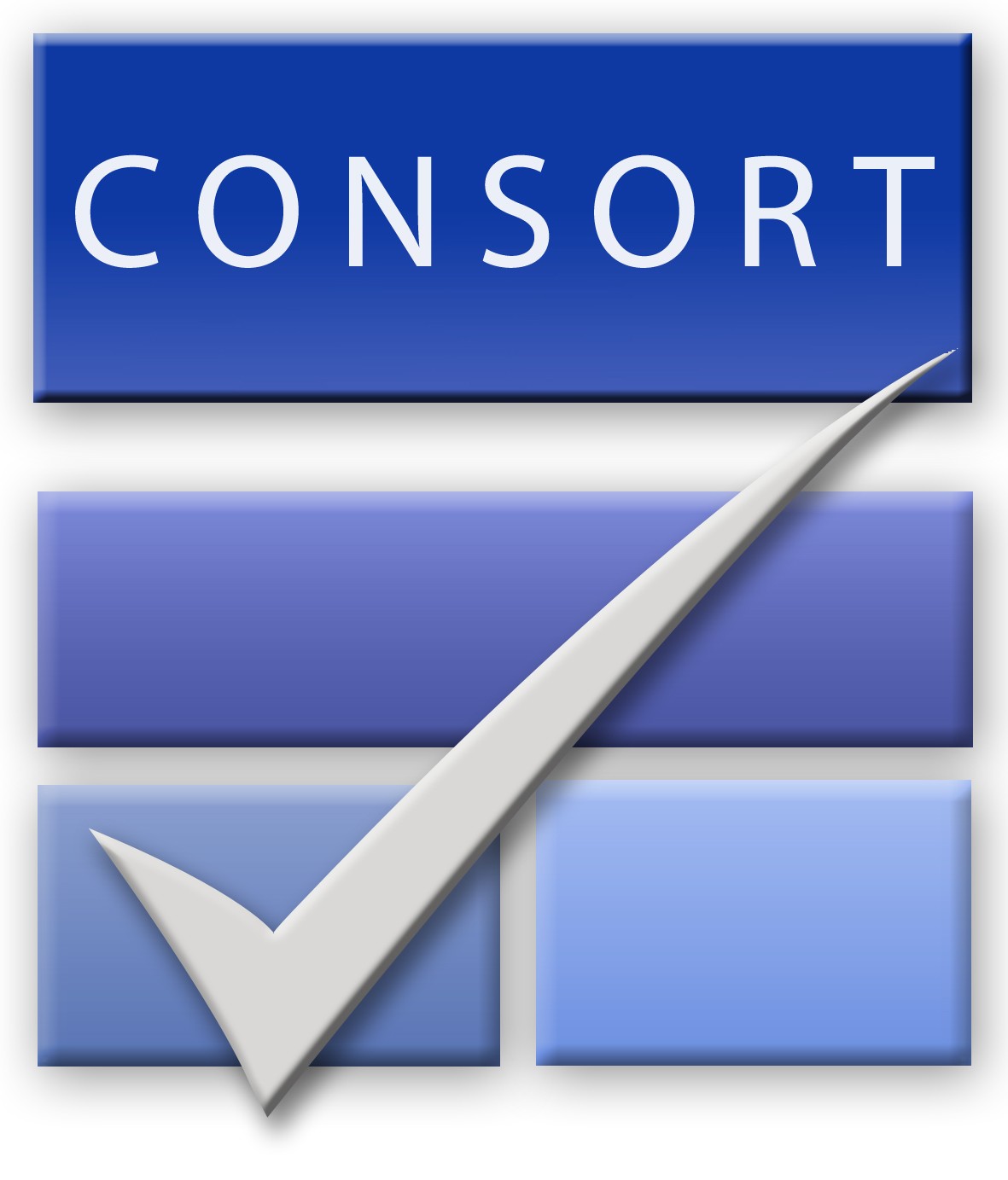
CONSORT 2010 checklist of information to include when reporting a randomised trial*

| Section/Topic | Item No | Checklist item | Reported on page No |
| --- | --- | --- | --- |
| Title and abstract | | | |
|  | 1a | Identification as a randomised trial in the title | YES in title |
|  | 1b | Structured summary of trial design, methods, results, and conclusions (for specific guidance see CONSORT for abstracts) | YES in abstract |
| Introduction | | | |
| Background and objectives | 2a | Scientific background and explanation of rationale | YES background |
|  | 2b | Specific objectives or hypotheses | YES background |
| Methods | | | |
| Trial design | 3a | Description of trial design (such as parallel, factorial) including allocation ratio | YES methods |
|  | 3b | Important changes to methods after trial commencement (such as eligibility criteria), with reasons | NO |
| Participants | 4a | Eligibility criteria for participants | YES methods |
|  | 4b | Settings and locations where the data were collected | YES methods |
| Interventions | 5 | The interventions for each group with sufficient details to allow replication, including how and when they were actually administered | YES methods |
| Outcomes | 6a | Completely defined pre-specified primary and secondary outcome measures, including how and when they were assessed | YES methods |
|  | 6b | Any changes to trial outcomes after the trial commenced, with reasons | NO |
| Sample size | 7a | How sample size was determined | YES methods |
|  | 7b | When applicable, explanation of any interim analyses and stopping guidelines | YES methods |
| Randomisation: |  |  |  |
| Sequence generation | 8a | Method used to generate the random allocation sequence | YES methods |
|  | 8b | Type of randomisation; details of any restriction (such as blocking and block size) | YES methods |
| Allocation concealment mechanism | 9 | Mechanism used to implement the random allocation sequence (such as sequentially numbered containers), describing any steps taken to conceal the sequence until interventions were assigned | YES methods |
| Implementation | 10 | Who generated the random allocation sequence, who enrolled participants, and who assigned participants to interventions | YES methods |
| Blinding | 11a | If done, who was blinded after assignment to interventions (for example, participants, care providers, those assessing outcomes) and how | YES methods |
|  | 11b | If relevant, description of the similarity of interventions | YES methods |
| Statistical methods | 12a | Statistical methods used to compare groups for primary and secondary outcomes | YES methods |
|  | 12b | Methods for additional analyses, such as subgroup analyses and adjusted analyses | YES methods |
| Results | | | |
| Participant flow (a diagram is strongly recommended) | 13a | For each group, the numbers of participants who were randomly assigned, received intended treatment, and were analysed for the primary outcome | YES abstract, results, Fig1 |
|  | 13b | For each group, losses and exclusions after randomisation, together with reasons | YES Fig1 & Tab5 |
| Recruitment | 14a | Dates defining the periods of recruitment and follow-up | YES results & fig1 |
|  | 14b | Why the trial ended or was stopped | YES results & discussion |
| Baseline data | 15 | A table showing baseline demographic and clinical characteristics for each group | YES Tab2 |
| Numbers analysed | 16 | For each group, number of participants (denominator) included in each analysis and whether the analysis was by original assigned groups | YES tab3&4, & results |
| Outcomes and estimation | 17a | For each primary and secondary outcome, results for each group, and the estimated effect size and its precision (such as 95% confidence interval) | YES results |
|  | 17b | For binary outcomes, presentation of both absolute and relative effect sizes is recommended | YES results |
| Ancillary analyses | 18 | Results of any other analyses performed, including subgroup analyses and adjusted analyses, distinguishing pre-specified from exploratory | YES results |
| Harms | 19 | All important harms or unintended effects in each group (for specific guidance see CONSORT for harms) | YES results &Tab4 |
| Discussion | | | |
| Limitations | 20 | Trial limitations, addressing sources of potential bias, imprecision, and, if relevant, multiplicity of analyses | YES discussion |
| Generalisability | 21 | Generalisability (external validity, applicability) of the trial findings | YES discussion |
| Interpretation | 22 | Interpretation consistent with results, balancing benefits and harms, and considering other relevant evidence | YES discussion |
| Other information | | |  |
| Registration | 23 | Registration number and name of trial registry | YES abstract and methods |
| Protocol | 24 | Where the full trial protocol can be accessed, if available | YES methods |
| Funding | 25 | Sources of funding and other support (such as supply of drugs), role of funders | YES |

*We strongly recommend reading this statement in conjunction with the CONSORT 2010 Explanation and Elaboration for important clarifications on all the items. If relevant, we also recommend reading CONSORT extensions for cluster randomised trials, non-inferiority and equivalence trials, non-pharmacological treatments, herbal interventions, and pragmatic trials. Additional extensions are forthcoming: for those and for up to date references relevant to this checklist, see [www.consort-statement.org](http://www.consort-statement.org/).
